# A Physiologically-Based Pharmacokinetic Model of Trimethoprim for MATE1, OCT1, OCT2, and CYP2C8 Drug–Drug–Gene Interaction Predictions

**DOI:** 10.3390/pharmaceutics12111074

**Published:** 2020-11-10

**Authors:** Denise Türk, Nina Hanke, Thorsten Lehr

**Affiliations:** Clinical Pharmacy, Saarland University, 66123 Saarbrücken, Germany; denise.tuerk@uni-saarland.de (D.T.); n.hanke@mx.uni-saarland.de (N.H.)

**Keywords:** physiologically-based pharmacokinetic (PBPK) modeling, trimethoprim, drug–drug interaction (DDI), multidrug and toxin extrusion protein (MATE), organic cation transporter (OCT), cytochrome P450 2C8 (CYP2C8)

## Abstract

Trimethoprim is a frequently-prescribed antibiotic and therefore likely to be co-administered with other medications, but it is also a potent inhibitor of multidrug and toxin extrusion protein (MATE) and a weak inhibitor of cytochrome P450 (CYP) 2C8. The aim of this work was to develop a physiologically-based pharmacokinetic (PBPK) model of trimethoprim to investigate and predict its drug–drug interactions (DDIs). The model was developed in PK-Sim^®^, using a large number of clinical studies (66 plasma concentration–time profiles with 36 corresponding fractions excreted in urine) to describe the trimethoprim pharmacokinetics over the entire published dosing range (40 to 960 mg). The key features of the model include intestinal efflux via P-glycoprotein (P-gp), metabolism by CYP3A4, an unspecific hepatic clearance process, and a renal clearance consisting of glomerular filtration and tubular secretion. The DDI performance of this new model was demonstrated by prediction of DDIs and drug–drug–gene interactions (DDGIs) of trimethoprim with metformin, repaglinide, pioglitazone, and rifampicin, with all predicted DDI and DDGI *AUC*_last_ and *C*_max_ ratios within 1.5-fold of the clinically-observed values. The model will be freely available in the Open Systems Pharmacology model repository, to support DDI studies during drug development.

## 1. Introduction

Trimethoprim is an inhibitor of bacterial folic acid metabolism used to treat bacterial infections. It is either applied as monotherapy or in combination with sulfonamides, e.g., sulfamethoxazole (“cotrimoxazole”). Trimethoprim is one of the most frequently-used antibiotics worldwide, ranking fifth after penicillins, cephalosporins, macrolides, and fluoroquinolones, with a global consumption of 5 × 10^9^ standard units in 2010 [1].

Due to the frequent prescription of trimethoprim, investigation of its drug–drug interaction (DDI) potential is clinically relevant. The antibiotic is a potent inhibitor of multidrug and toxin-extrusion protein (MATE) 1 and MATE2-K [2], and therefore recommended by the FDA as a clinical MATE inhibitor. Furthermore, trimethoprim less potently inhibits organic cation transporter (OCT) 1 and OCT2 [3,4]. This combined inhibition potential can be observed during clinical studies of trimethoprim with metformin, where co-administration of trimethoprim increases the area under the concentration–time curve (*AUC*) of metformin by 30% [4]. Metformin is listed by the FDA as the only recommended MATE1, MATE2-K, and OCT2 substrate for clinical DDI studies [2].

In addition to its inhibition of transporters, trimethoprim is a weak inhibitor of cytochrome P450 (CYP) 2C8 [2]. Co-administration of trimethoprim increases the *AUC* of repaglinide and pioglitazone by 61% and 42%, respectively [5,6]. Both victim drugs are mainly metabolized by CYP2C8 and listed as sensitive CYP2C8 index substrate (repaglinide) or moderately-sensitive CYP2C8 substrate (pioglitazone) for the use in clinical DDI studies [2].

Similar to DDIs, polymorphisms in transporters or metabolizing enzymes can affect the pharmacokinetics of a drug (drug–gene interactions, DGIs), leading to loss of efficacy or adverse drug reactions. Naturally, DDIs and DGIs may occur simultaneously (drug–drug–gene interactions, DDGIs), counteracting or adding their respective effects on drug exposure, which urgently needs to be considered in clinical practice as it can lead to very strong interaction effects. Two DDGIs with trimethoprim as the perpetrator, co-administered with metformin in *SLC22A2 808G>T* polymorphic subjects or with pioglitazone in CYP2C8*3 polymorphic volunteers, are reported in the literature [6,7]. The *SLC22A2 808G>T* allele frequency is between 10 and 14% in most populations [8]. This missense variant is associated with decreased metformin maximum plasma concentrations (*C*_max_) in vivo [7,9,10,11] and might be associated with cisplatin-induced ototoxicity [12]. The CYP2C8*3 allele frequency varies between populations and is reported at 13% in Caucasians and 2% in African Americans [13]. In vitro data suggest that the CYP2C8*3 allele is associated with decreased metabolism of e.g., paclitaxel [13]. However, clinical data showed controversial results with increased metabolism of repaglinide and pioglitazone [6,14].

In addition to its DDI liability as a perpetrator drug, trimethoprim can also be the victim drug in polypharmaceutical drug regimens. Co-administration of trimethoprim with rifampicin, an inducer of CYP enzymes and P-glycoprotein (P-gp) [2,15], has been shown to increase the urinary excretion of trimethoprim due to increased expression of P-gp [16].

The aims of this study were (1) to develop a whole-body physiologically-based pharmacokinetic (PBPK) model of trimethoprim that accurately describes the observed concentrations in plasma and urine over time, (2) to predict the DDIs of trimethoprim with the victim drugs metformin, repaglinide and pioglitazone, (3) to predict the clinically-significant DDGIs of trimethoprim with metformin in *SLC22A2 808G>T* carriers and with pioglitazone in CYP2C8*3 carriers, and (4) to describe the rifampicin–trimethoprim DDI with trimethoprim in the role of the victim drug. The newly-developed trimethoprim model will be freely available in the Open Systems Pharmacology model repository (www.open-systems-pharmacology.org) and the Supplementary Materials to this manuscript were compiled as one comprehensive reference manual with transparent documentation of the model performance to support DDI investigations during drug development, labeling, and submission for regulatory approval of new drugs.

## 2. Materials and Methods

### 2.1. Software

The PBPK model of trimethoprim was developed using PK-Sim^®^ modeling software (Open Systems Pharmacology Suite 8.0, www.open-systems-pharmacology.org, 2019). Clinical study data from literature were digitized with Engauge Digitizer 10.12 (© M. Mitchell [17], 2019) according to best practices [18]. Model parameter optimization (Levenberg–Marquardt algorithm) and sensitivity analysis were performed within PK-Sim^®^. Calculation of pharmacokinetic parameters, quantitative model performance analysis, and generation of plots were accomplished using R 3.6.2 (The R Foundation for Statistical Computing, Vienna, Austria, 2019) and RStudio 1.2.5033 (RStudio, Inc., Boston, MA, USA, 2019).

### 2.2. Trimethoprim Clinical Data

Plasma or whole blood concentration–time profiles and fraction excreted unchanged (*f*e) in urine data of single- and multiple-dose trimethoprim studies were collected from literature and digitized. The obtained profiles were divided into a training dataset and a test dataset, which were used for model building and model evaluation, respectively.

### 2.3. Trimethoprim PBPK Model Building

Model building was started with an extensive literature search to gain information about physicochemical parameters as well as absorption, distribution, metabolism, and excretion (ADME) processes of trimethoprim.

To simulate trimethoprim in the different organs of the body, virtual individuals were created according to the demographics of the respective clinical studies (ethnicity, sex, age, body weight, and height). If no information was provided, a European, male, 30-year-old individual was assumed, with body weight and height characteristics taken from the PK-Sim^®^ population database.

Transporters and enzymes involved in trimethoprim ADME were implemented according to current literature, using the PK-Sim^®^ expression database [19]. Details on their expression and localization in the different organs of the body are provided in the system-dependent parameter table in the Supplementary Materials (Table S19).

Model parameters that could not be informed from literature were optimized by fitting the model simultaneously to all plasma or whole blood concentration–time profiles and *f*e in urine data of the training dataset.

### 2.4. Trimethoprim PBPK Model Evaluation

Trimethoprim model performance was evaluated by comparison of (1) the predicted plasma or whole blood concentration-time and *f*e in urine profiles to the clinically-observed data of the respective clinical studies, (2) predicted plasma or whole blood concentration values of all studies to their corresponding observed values in goodness-of-fit plots, and (3) predicted to observed *f*e in urine, *AUC*, and *C*_max_ values, where *AUC* was calculated from the time of drug administration to the time of the last concentration measurement (*AUC*_last_) for both predicted and observed plasma or whole blood concentration–time profiles.

As quantitative measures of the model performance, the mean relative deviation (MRD) of all predicted plasma and whole blood concentrations and the geometric mean fold error (GMFE) of all predicted *f*e in urine, *AUC*_last_, and *C*_max_ values were calculated according to Equations (1) and (2), respectively. MRD and GMFE values ≤ 2 characterize an adequate model performance.

(1) $${\mathrm{MRD}\;=\;10}^{x};\;x\;=\;\sqrt{\frac{\sum_{i=1}^{k}{\;(\log}_{10}c_{\mathrm{predicted},i}\;-\;\log_{10}c_{\mathrm{observed},i}{)}^{2}}{k}}$$

where *c*_predicted,i_ = predicted plasma (or whole blood) concentration, *c*_observed,i_ = corresponding observed plasma (or whole blood) concentration, and *k* = number of observed values.

(2) $${\mathrm{GMFE}\;=\;10}^{x};\;x\;=\;\frac{\sum_{i=1}^{m}\;|\;\log_{10}\;\left(\frac{{\mathrm{predicted}\;\mathrm{PK}\;\mathrm{parameter}}_{i}}{{\mathrm{observed}\;\mathrm{PK}\;\mathrm{parameter}}_{i}}\right)|\;}{m}$$

where predicted PK parameter_i_ = predicted *f*e in urine, *AUC*_last_, or *C*_max_ value; observed PK parameter_i_ = corresponding observed *f*e in urine, *AUC*_last_, or *C*_max_ value; *m* = number of studies.

### 2.5. DDI and DDGI Modeling

In addition to the previously-described methods for PBPK model evaluation, the ability of the trimethoprim model to adequately predict DDIs was tested. Trimethoprim DD(G)I modeling was performed with three different victim drugs (metformin, repaglinide, and pioglitazone) and one perpetrator drug (rifampicin). The parameters of the previously-developed PBPK models of metformin [20], repaglinide, pioglitazone [21], and rifampicin [22] that were applied for DDI modeling are reproduced in Tables S8, S11, S14 and S17 and DDI model processes are illustrated in Figures S14, S18, S21 and S25 of the Supplementary Materials.

To predict the trimethoprim–metformin DDI, competitive inhibition of MATE1, OCT1, and OCT2 by trimethoprim was implemented, using *K*_i_ values of 4.45 µmol/L, 32.20 µmol/L, and 47.82 µmol/L, respectively [3,4,23,24,25,26,27]. The trimethoprim–repaglinide and trimethoprim–pioglitazone DDIs were predicted as competitive inhibition of CYP2C8 by trimethoprim with a *K*_i_ value of 4.85 µmol/L [28]. All trimethoprim *K*_i_ values and in vitro references are listed in the trimethoprim drug-dependent parameter table in Section 3.1.

To predict the published trimethoprim DDGI studies with metformin (*SLC22A2 808G>T*, increased metformin transport) and pioglitazone (CYP2C8*3, increased pioglitazone metabolism), the trimethoprim model was applied with previously-built and evaluated DGI models of metformin and pioglitazone [20,21]. For the competitive inhibition of the variant OCT2 or CYP2C8 isoforms by trimethoprim, the same *K*_i_ values as for the wildtype transporter or enzyme were applied.

The rifampicin–trimethoprim DDI was modeled as induction of P-gp trimethoprim transport and CYP3A4 trimethoprim metabolism by rifampicin, with simultaneous competitive inhibition of P-gp and CYP3A4. The parameter values to model these interactions were taken from literature (values and references are listed in the rifampicin drug-dependent parameter Table S17 in the Supplementary Materials) and have been evaluated in previous DDI analyses [21,22]. Due to the lack of in vitro information regarding the metabolism of trimethoprim, the clinical data of the rifampicin–trimethoprim DDI were included into the training dataset, and the inducible fraction of trimethoprim metabolism was attributed to metabolism by CYP3A4.

The mathematical implementation of competitive inhibition and rifampicin-dependent induction is shown in Section 1.5 of the Supplementary Materials.

### 2.6. DDI and DDGI Model Performance Evaluation

The DDI and DDGI modeling performance was evaluated by comparison of predicted to observed plasma concentration–time profiles of the respective victim drugs metformin, repaglinide, pioglitazone, or trimethoprim, administered alone and during perpetrator drug co-treatment (trimethoprim or rifampicin). Furthermore, predicted DDI or DDGI *AUC*_last_ ratios (Equation (3)) and DDI or DDGI *C*_max_ ratios (Equation (4)) were calculated, and compared to the observed ratios.

(3) $$\mathrm{DDI}\;\mathrm{or}\;\mathrm{DDGI}\;{AUC}_{\mathrm{last}}\;\mathrm{ratio}\;=\;\frac{{AUC}_{\mathrm{last}}\;\mathrm{victim}\;\mathrm{drug}\;\mathrm{during}\;\mathrm{co}-\mathrm{administration}}{{AUC}_{\mathrm{last}}\;\mathrm{victim}\;\mathrm{drug}\;\mathrm{control}}$$

(4) $$\mathrm{DDI}\;\mathrm{or}\;\mathrm{DDGI}\;C_{\max}\;\mathrm{ratio}\;=\;\frac{C_{\max}\;\mathrm{victim}\;\mathrm{drug}\;\mathrm{during}\;\mathrm{co}-\mathrm{administration}}{C_{\max}\;\mathrm{victim}\;\mathrm{drug}\;\mathrm{control}}$$

As a quantitative measure of the DDI and DDGI model performance, GMFE values of the predicted *AUC*_last_ ratios and *C*_max_ ratios were calculated according to Equation (2).

### 2.7. Sensitivity Analysis

Local sensitivity analysis was performed on the trimethoprim model to investigate the impact of single-model parameters on the predicted *AUC*, *C*_max_, and *t*_max_ at steady state. Parameters were included in the analysis if they have been optimized, if they were associated with optimized parameters or if they might had a strong impact on the model predictions due to their use in the calculation of permeabilities or partition coefficients. A list of the analyzed parameters is provided in Table S6 of the Supplementary Materials.

Sensitivity was calculated as the ratio of the relative change of the simulated *AUC*, *C*_max_, or *t*_max_ to the relative variation of the tested parameter around the parameter value used in the model, according to Equation (5):

(5) $$S\;=\;\frac{\Delta PK}{PK}\cdot \frac{p}{\Delta p}$$

where *S* = sensitivity of the *AUC*, *C*_max_, or *t*_max_ to the tested model parameter; Δ*PK* = change of the *AUC*, *C*_max_, or *t*_max_; *PK* = *AUC*, *C*_max_, or *t*_max_ predicted with the original model parameter value; *p* = original model parameter value; Δ*p* = change of the tested model parameter value.

Sensitivity analysis was performed using the highest recommended dose and a relative parameter perturbation of 1000%. The threshold value for sensitivity was set to 0.5; this value signifies that a 100% change of the investigated parameter value causes a 50% change of the predicted *AUC*, *C*_max_ or *t*_max_.

## 3. Results

### 3.1. Trimethoprim PBPK Model Building and Evaluation

The trimethoprim whole-body PBPK model was built and evaluated using a total number of 66 trimethoprim plasma or whole blood concentration–time profiles and 36 *f*e in urine profiles (intravenous and oral, single-, and multiple-dose administration), covering a broad dosing range from of 40 to 960 mg. In 47 of the 66 clinical studies, trimethoprim was administered as “cotrimoxazole”, i.e., in combination with sulfamethoxazole. According to literature [29,30] and our own analyses, trimethoprim pharmacokinetic profiles are not altered by simultaneous administration of sulfamethoxazole (see Figure S2 in the Supplementary Materials). Consequently, studies with co-administration of trimethoprim and sulfamethoxazole were included for model development. A table listing all utilized clinical studies is provided in the Supplementary Materials (Table S1).

**Figure 1:**
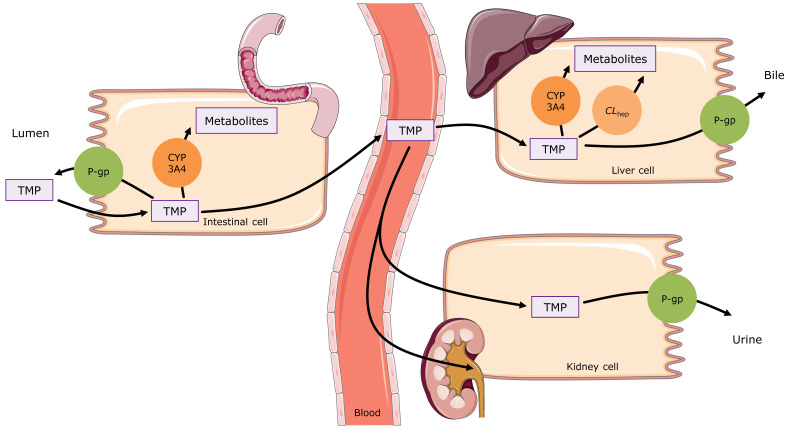
Schematic illustration of the trimethoprim absorption, distribution, metabolism, and excretion (ADME) processes in the model. Trimethoprim is absorbed in the intestine with counteractive efflux via P-gp. About 20% of a trimethoprim dose is metabolized [33] (modeled via CYP3A4 and an additional *CL*_hep_). The main route of trimethoprim elimination is urinary excretion (46–67% of an oral dose [30,31,32]) via glomerular filtration and active tubular secretion via P-gp. Drawings by Servier, licensed under CC BY 3.0 [34]. *CL*_hep_: hepatic metabolic clearance, CYP: cytochrome P450, P-gp: P-glycoprotein, TMP: trimethoprim.

The final trimethoprim PBPK model applies active efflux via P-gp (most strongly expressed in the intestine and kidney), metabolism by CYP3A4 (mainly in the liver with lower expression in the intestine), an unspecific hepatic clearance, and passive glomerular filtration. Trimethoprim is primarily excreted unchanged in the urine (46–67% of an oral dose [30,31,32]). The implemented ADME processes are visualized in Figure 1 and in Figure S3 of the Supplementary Materials. The drug-dependent parameters of the final model are given in Table 1 and in Table S2 of the Supplementary Materials. The model-specific, system-dependent parameters, with the expression profiles of the incorporated transporter and metabolizing enzymes, are summarized in the system-dependent parameter table in the Supplementary Materials (Table S19).

**Figure 2:**
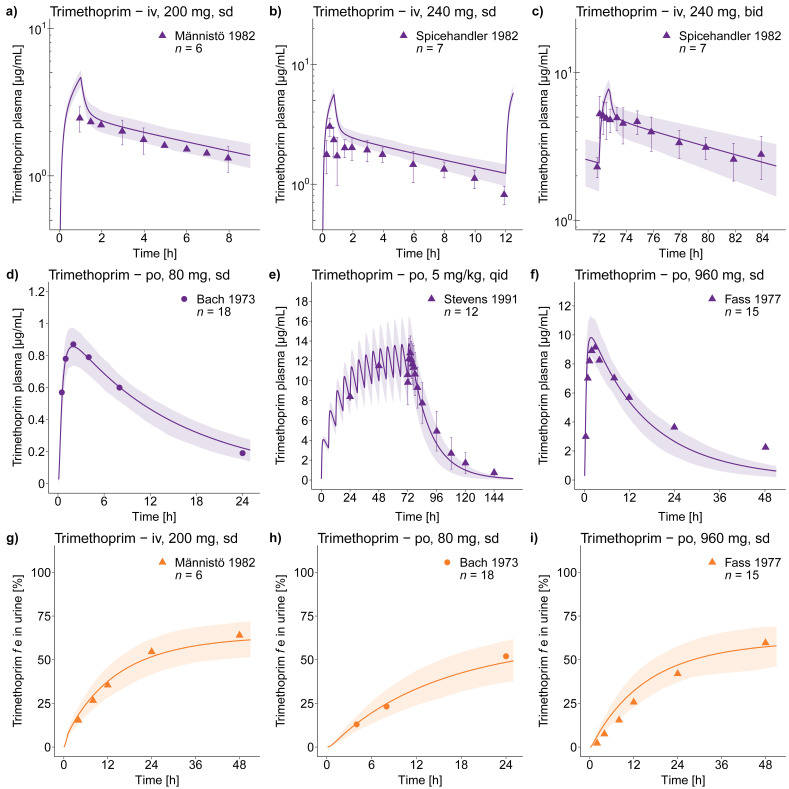
Trimethoprim in plasma and urine. Population predictions of trimethoprim (**a**–**f**) plasma concentration–time profiles and (**g**–**i**) fraction excreted unchanged in urine profiles compared to observed data [43,54,55,57,58] of representative intravenous and oral studies. Population prediction arithmetic means are shown as lines; the shaded areas illustrate the 68% population prediction intervals. Observed data are shown as triangles (training dataset) or dots (test dataset) ± standard deviation. Details on the study protocols and model simulations of all 66 clinical studies used for model building and evaluation are provided in the Supplementary Materials. bid, twice daily; *f*e in urine, fraction excreted unchanged in urine; iv, intravenous; po, oral; qid, four times daily; sd, single dose.

**Figure 3:**
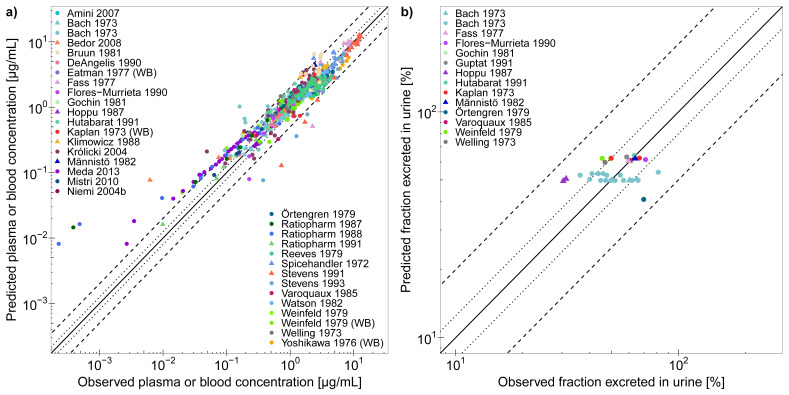
Trimethoprim physiologically-based pharmacokinetic (PBPK) model performance. The goodness-of-fit plots show predicted compared to observed (**a**) plasma or whole blood concentrations and (**b**) fractions excreted unchanged in urine of all studies used for model building and evaluation. The solid line marks the line of identity and dotted lines indicate 1.25-fold and dashed lines indicate 2-fold deviation. Data are shown as triangles (training dataset) or dots (test dataset) [30,31,33,37,41,43,46,51,52,53,54,55,56,57,58,59,60,61,62,63,64,65,66,67,68,69,70,71,72,73]. Details on the predicted clinical studies and the individual fraction excreted unchanged in urine values are provided in the Supplementary Materials. WB, whole blood.

The good descriptive (training dataset, 13 studies) and predictive (test dataset, 53 studies) performance of the trimethoprim model is demonstrated in Figure 2, showing representative population predictions of plasma concentration–time profiles and *f*e in urine, compared to observed data. The population predictions of all 66 analyzed clinical studies, compared to their respective observed data, are shown in Figures S4–S9 of the Supplementary Materials (semilogarithmic as well as linear plots). Furthermore, goodness-of-fit plots with predicted versus observed (a) plasma or whole blood concentrations and (b) *f*e in urine values, are presented in Figure 3 and in Figures S10 and S11 of the Supplementary Materials, where 93% of all predicted plasma or whole blood concentrations and 100% of all predicted *f*e in urine values are within 2-fold of the observed data. MRD values for all predicted plasma or whole blood concentration–time profiles (58/66 with MRD ≤ 2) and GMFE values for predicted *f*e in urine (overall GMFE of 1.19) are documented in the Supplementary Materials (Tables S3 and S4).

**Figure 4:**
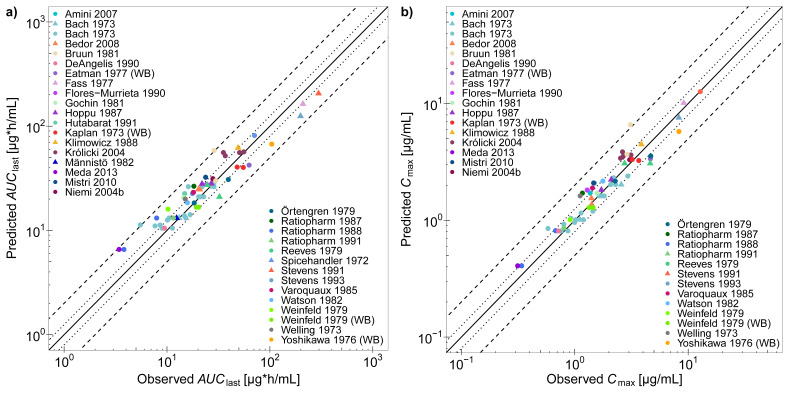
Trimethoprim PBPK model performance. The goodness-of-fit plots show predicted compared to observed (**a**) *AUC*_last_ values and (**b**) *C*_max_ values of all studies used for model building and evaluation. The solid line marks the line of identity and dotted lines indicate 1.25-fold and dashed lines indicate 2-fold deviation. Data are shown as triangles (training dataset) or dots (test dataset) [30,31,33,37,41,43,46,51,52,53,54,55,56,57,58,59,60,61,62,63,64,65,66,67,68,69,70,71,72,73]. Details on the predicted clinical studies and the individual *AUC*_last_ and *C*_max_ values are provided in the Supplementary Materials. WB, whole blood.

Correlations of predicted with observed *AUC*_last_ (97% within 2-fold) and *C*_max_ values (98% within 2-fold) are presented in Figure 4 and in Figure S12 of the Supplementary Materials. The plotted values for all studies are provided in the Supplementary Materials (Table S5), including calculated GMFE values, with overall GMFEs of 1.29 and 1.20 for *AUC*_last_ and *C*_max_, respectively.

Sensitivity analysis of a simulation of 160 mg trimethoprim twice daily, using a parameter perturbation of 1000% and a sensitivity threshold of 0.5, showed that the only parameter value the model predictions are sensitive to is the trimethoprim fraction unbound in plasma, for which a literature value is used in the model (56% [44]). The full quantitative results of the sensitivity analysis are shown in Section 2.5 (Figure S13) of the Supplementary Materials.

### 3.2. Trimethoprim DDI and DDGI Modeling

**Figure 5:**
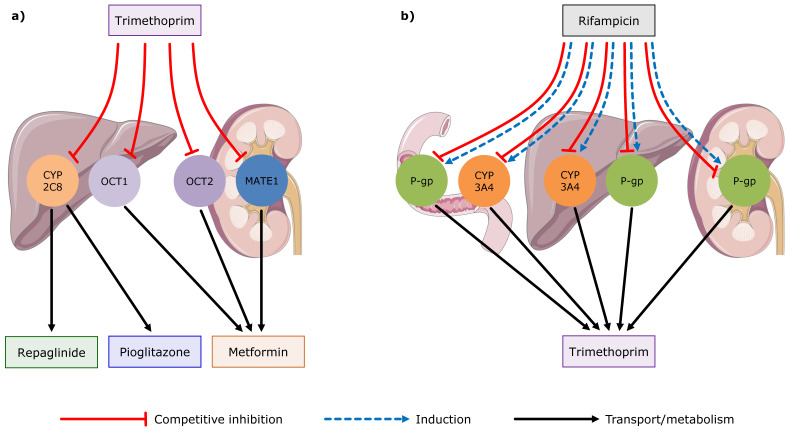
Trimethoprim drug–drug interaction (DDI) network. (**a**) Trimethoprim is a MATE1, OCT1, OCT2 and CYP2C8 inhibitor that impacts the pharmacokinetics of metformin, repaglinide, and pioglitazone. (**b**) On the other hand, trimethoprim is a victim drug in the DDI with rifampicin. Rifampicin inhibits and in the long term induces P-gp and CYP3A4, and thereby impacts the pharmacokinetics of trimethoprim. Drawings by Servier, licensed under CC BY 3.0 [34]. CYP: cytochrome P450, MATE: multidrug and toxin extrusion protein, OCT: organic cation transporter, P-gp: P-glycoprotein.

Trimethoprim DD(G)I modeling was performed with three different victim drugs (metformin, repaglinide, and pioglitazone) and one perpetrator drug (rifampicin). Tables listing all utilized clinical DDI studies are provided in the Supplementary Materials (Tables S7, S10, S13 and S16). The resulting trimethoprim DDI network with the affected transporters and enzymes is illustrated in Figure 5 and in Figure S1 of the Supplementary Materials.

**Figure 6:**
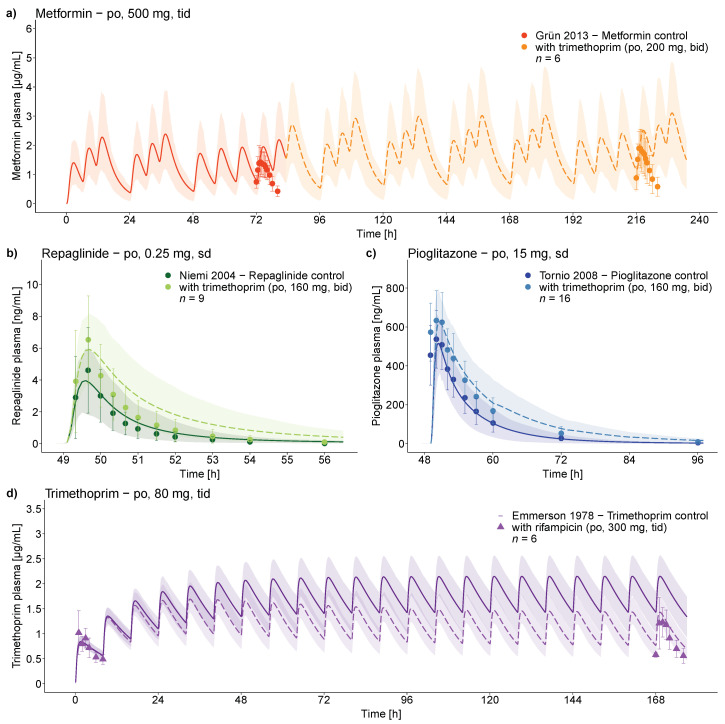
Trimethoprim DDI victim drug plasma profiles. Population predictions of victim drug plasma concentration–time profiles of the (**a**) trimethoprim–metformin, (**b**) trimethoprim–repaglinide, (**c**) trimethoprim–pioglitazone, and (**d**) rifampicin–trimethoprim DDIs, compared to observed data [5,6,7,16]. Population prediction arithmetic means are shown as lines (solid, victim drug alone and dashed, victim drug during perpetrator co-administration); the shaded areas illustrate the respective 68% population prediction intervals. Observed data are shown as triangles (training dataset) or dots (test dataset) ± standard deviation. Perpetrator application starts at (**a**) 83 h or (**b**–**d**) 0 h. Details on the study protocols and model simulations of all clinical DDI and DDGI studies used to evaluate the DDI performance of the trimethoprim model are provided in the Supplementary Materials. bid, twice daily; po, oral; sd, single dose; tid, three times daily.

The good DDI model performance is demonstrated in Figure 6, showing representative population predictions of victim drug plasma concentration–time profiles before and during the four different DDIs, compared to observed data. For the rifampicin–trimethoprim DDI study, no plasma concentrations of trimethoprim without rifampicin co-administration were reported. Instead, day 1 and day 8 of the rifampicin–trimethoprim co-administration were shown, and therefore modeled and evaluated. Semilogarithmic as well as linear plots of population predicted compared to observed victim drug plasma concentration–time profiles of all DDI and DDGI studies are shown in Figures S15, S16, S19, S22, S23, S26, and S27 of the Supplementary Materials.

**Figure 7:**
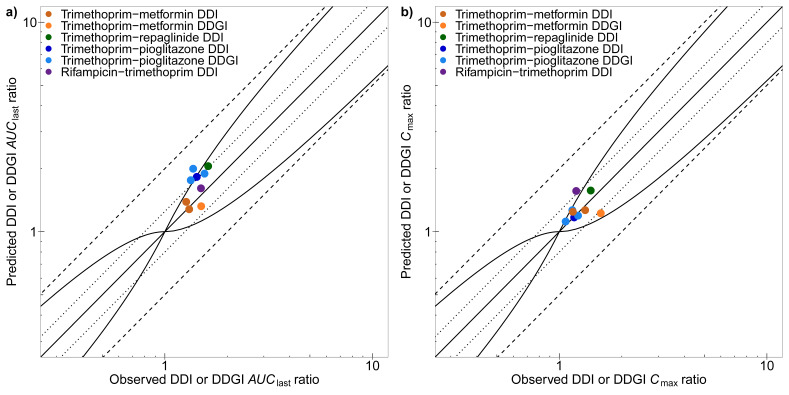
Trimethoprim DDI model performance. Predicted compared to observed DDI and DDGI (**a**) *AUC*_last_ ratios and (**b**) *C*_max_ ratios of all clinical studies used to evaluate the DDI performance of the trimethoprim model. The straight solid line marks the line of identity and the curved solid lines show the DDI prediction acceptance limits proposed by Guest et al. [74]. Dotted lines indicate 1.25-fold and dashed lines indicate 2-fold deviation. Data are shown as dots [4,5,6,7,16]. Details on the predicted clinical studies and the individual DDI and DDGI *AUC*_last_ and *C*_max_ ratios are provided in the Supplementary Materials.

For a quantitative evaluation of the DDI performance, predicted and observed DDI and DDGI *AUC*_last_, and *C*_max_ ratios are compared in Figure 7 and listed in the Supplementary Materials, showing overall GMFEs of 1.08, 1.27, 1.32, and 1.08 (*AUC*_last_ ratios) and of 1.14, 1.11, 1.04, and 1.30 (*C*_max_ ratios) for the four modeled DDIs (trimethoprim–metformin, trimethoprim–repaglinide, trimethoprim–pioglitazone, and rifampicin–trimethoprim), respectively. All predicted DDI and DDGI *AUC*_last_ and *C*_max_ ratios are within 1.5-fold of the observed values. The full quantitative evaluation showing all ratios and GMFE values with ranges is presented in the Supplementary Materials (Tables S9, S12, S15 and S18 and Figures S17, S20, S24 and S28).

## 4. Discussion

A whole-body PBPK model of trimethoprim for the investigation and prediction of DDIs has been successfully built and evaluated. The model reliably captures the trimethoprim plasma and urine concentration–time profiles over a broad dosing range, for intravenous and oral administration as well as for single- and multiple-dose regimens. Good model performance has been demonstrated by (1) comparison of population predicted plasma or whole blood concentration and *f*e in urine profiles to observed data, (2) a goodness-of-fit plot and MRD values of the predicted plasma concentrations, (3) goodness-of-fit plots and GMFE values of the predicted *f*e in urine, *AUC*_last_, and *C*_max_ values, and (4) the good DDI and DDGI performance.

The processes involved in the absorption, distribution, metabolism, and excretion of trimethoprim are not completely characterized or understood. It is known that trimethoprim is mainly excreted unchanged in urine (46–67% of an oral dose [30,31,32]), via glomerular filtration and tubular secretion. In vitro, trimethoprim is a substrate of P-gp [75], MATE1, and MATE2-K [76], but MATE2-K expression in the human kidney is extremely low [77]. The active tubular secretion of trimethoprim via MATE1 also seems unlikely, because the renal clearance of trimethoprim increased after eight days of rifampicin co-administration [16], and induction of MATE1 by rifampicin has not been demonstrated, yet. Furthermore, about 20% of a trimethoprim dose is reported to be metabolized [33], but there is no information available, as to which enzymes are involved in vivo. Implementation of P-gp and CYP3A4 into the trimethoprim model resulted in a good description of the trimethoprim concentration–time profiles observed in plasma and urine. In addition, the trimethoprim plasma concentrations measured during the first and eighth day of rifampicin co-administration and the observed increase in trimethoprim renal clearance on the eighth day of this DDI [16] are well captured by the model after implementation of P-gp and CYP3A4. Another candidate enzyme for trimethoprim metabolism in vivo is CYP2C9 [78], but co-administration of high doses of the CYP2C9 inhibitor, sulfamethoxazole (*K*i = 271 µM [79]) showed no effect on trimethoprim plasma concentrations (see [29,30] and Figure S2 in the Supplementary Materials). In addition, the induction of CYP2C9 by rifampicin is not as strong as that of CYP3A4 and using CYP2C9 as the main enzyme for trimethoprim metabolism in the model resulted in an underprediction of the rifampicin DDI effect.

One shortcoming of the presented model might be that according to literature, about 20% of a trimethoprim dose is metabolized [33], but fitting the model to this low value (CYP3A4 metabolism assumed) led to an overprediction of the urinary excretion and to an underprediction of the rifampicin-trimethoprim DDI. By implementation of CYP3A4 metabolism and addition of an unspecific hepatic clearance process, both urinary excretion and rifampicin–trimethoprim DDI could be well described, accepting a higher total fraction metabolized of 30–40%. Summed up, these 30–40% match well with the observed 46–67% of trimethoprim excreted unchanged in urine [30,31,32] and the reported fraction excreted in feces of 4% [80]. Unfortunately, the in vivo trimethoprim metabolism is not completely understood, which led us to include an unspecific clearance into the model. It might be speculated that trimethoprim undergoes tubular reabsorption, which was not implemented in our model but could reduce the slight overprediction of trimethoprim urinary excretion that we see without the unspecific hepatic clearance. However, no transporters involved in tubular reabsorption of trimethoprim are described in the literature, so far. Therefore, the extent of trimethoprim metabolism and the involved enzymes, as well as possible tubular reabsorption mechanisms need to be further investigated experimentally, to confirm or reject our model assumptions.

The presented trimethoprim model is able to adequately predict the MATE1, OCT1, and OCT2 DDI (metformin) as well as the CYP2C8 DDIs (repaglinide and pioglitazone), shown by comparison of predicted to observed plasma concentration–time profiles and predicted compared to observed DDI *AUC* and *C*_max_ ratios, with all predicted ratios within 1.5-fold of the observed ratios. Metformin, the only recommended MATE1, MATE2-K, and OCT2 substrate for clinical DDI studies [2], is frequently prescribed (almost 80 million prescriptions in the USA in 2017 [81]) to treat type 2 diabetes mellitus. Also, as trimethoprim is regularly prescribed, co-administration with metformin, leading to increased metformin exposure, can frequently occur. The resulting increased risk of adverse drug events, e.g., in patients treated with high metformin doses or patients with impaired renal function, could be mitigated by applying the model to calculate metformin dose adaptations for the duration of this co-administration.

In addition, the model was successfully applied to predict plasma concentration–time profiles of metformin and pioglitazone in carriers of the *SLC22A2 808G>T* and CYP2C8*3 alleles, respectively, during co-administration with trimethoprim. The *SLC22A2 808G>T* allele investigated in this study occurs with a global frequency of 10–14% [8]. Therefore, investigation of its related DDGIs is clinically relevant. Plasma concentration time–profiles are well predicted using an OCT2 *K*_i_ value from in vitro literature (same value assumed for wildtype and polymorphic transporter), resulting in predicted DDGI *AUC* and *C*_max_ ratios within 1.5-fold of the observed values. The second variant allele investigated is the CYP2C8*3 allele, occurring with a frequency of 13% in Caucasians [13]. The model was applied to predict the trimethoprim–pioglitazone DDGI using a CYP2C8 *K*_i_ value taken from in vitro literature. For the DDGI, no plasma concentration–time profiles were reported and therefore, only predicted and observed DDGI *AUC* and *C*_max_ ratios were compared, resulting in predicted DDGI *AUC* and *C*_max_ ratios within 1.5-fold and 1.25-fold of observed values, respectively.

Regarding previously-published PBPK models of trimethoprim, there are four earlier models of trimethoprim described in the literature [82,83,84,85]. These models have been built to predict the CYP2C8 DDI and DDGI with rosiglitazone (whole-body PBPK model) [82], to investigate the basolateral and apical kidney transporter DDI with creatinine (two semi-PBPK models) [83,84], or for pediatric scaling (whole-body PBPK model) [85]. The trimethoprim–rosiglitazone DDGI model [82] well describes the rosiglitazone plasma concentration–time profiles in CYP2C8 wildtype and carriers of the CYP2C8*3 allele. Also, the two minimal PBPK models built to describe the creatinine plasma concentration–time profiles during trimethoprim co-administration show a good DDI performance [83,84], without taking *SLC22A2* polymorphism into account. Our model was built and evaluated to assess DDIs mediated via CYP2C8, MATE1, OCT1, and OCT2, as well as DDGIs caused by CYP2C8*3 and *SLC22A2808G>T* polymorphisms, applying one and the same whole-body PBPK model. Our model differs further from the previously-published models, as (1) none of these models was developed using such a large number of clinical studies (66 blood and 36 urine profiles) and (2) this is the first model which attempts to mechanistically describe the tubular secretion of trimethoprim. The good ability of the presented model to describe these different DDIs and DDGIs increases the confidence regarding the modeled trimethoprim concentrations at different sites of action (liver and kidney) and its general applicability for future investigations.

## 5. Conclusions

In this study, a carefully-developed mechanistic whole-body PBPK model of trimethoprim is presented. The model adequately predicts the trimethoprim pharmacokinetics following intravenous and oral administration over a broad range of dosing regimens. In addition, the model was qualified by prediction of DDI studies with the victim drugs metformin, repaglinide, and pioglitazone and by prediction of DDGI studies with metformin and pioglitazone. The model evaluation is transparently documented in the Supplementary Materials, showing the model performance for all 66 analyzed trimethoprim studies as well as for all DDI and DDGI studies utilized for model evaluation. The model will be shared with the research and drug development community via the Open Systems Pharmacology repository (www.open-systems-pharmacology.org) [86], for the investigation of new DDI scenarios with MATE1, OCT1, OCT2, and CYP2C8 victim drugs.

## Figures and Tables

**Table 1:** Trimethoprim drug-dependent parameters.

Parameter	Value	Unit	Source	Literature	Reference	Description
MW	290.32	g/mol	Literature	290.32	[35]	Molecular weight
p*K*_a_ (base)	7.12	-	Literature	6.60, 7.12, 7.30	[35,36,37]	Acid dissociation constant
Solubility (pH 7.0)	0.40	g/L	Literature	0.40	[35]	Solubility
log*P*	1.01	-	Optimized	0.60, 0.73, 0.91, 1.43	[35,38,39,40]	Lipophilicity
*f*u	56	%	Literature	42–65	[41,42,43,44,45,46,47]	Fraction unbound plasma
P-gp *K*_M_	195.75	µmol/L	Optimized	-	-	Michaelis –Menten constant
P-gp *k*_cat_	1.44	1/min	Optimized	-	-	Transport rate constant
CYP3A4 *K*_M_	375.57	µmol/L	Optimized	-	-	Michaelis–Menten constant
CYP3A4 *k*_cat_	0.56	1/min	Optimized	-	-	Catalytic rate constant
*CL*_hep_	1.61 × 10^−2^	1/min	Optimized	-	-	Hepatic metabolic clearance
GFR fraction	1	-	Assumed	-	-	Fraction of filtered drug in the urine
EHC continuous fraction	1	-	Assumed	-	-	Fraction of bile continually released
MATE1 *K*_i_	4.45	µmol/L	Literature	0.51, 2.64, 3.29, 3.94, 4.06, 4.58, 6.30, 6.73, 7.99 *	[3,4,23,24,25]	Conc. for 50% inhibition (competitive)
OCT1 *K*_i_	32.20	µmol/L	Literature	27.70, 36.70 *	[3,4]	Conc. for 50% inhibition (competitive)
OCT2 *K*_i_	47.82	µmol/L	Literature	13.20, 19.80, 27.20, 32.30, 57.40, 137.00 *	[3,4,23,26,27]	Conc. for 50% inhibition (competitive)
CYP2C8 *K*_i_	4.85	µmol/L	Literature	2.25, 3.80, 8.50 *	[28]	Conc. for 50% inhibition (competitive)
Partition coefficients	Diverse	-	Calculated	Berezhkovskiy	[48]	Cell to plasma partition coefficients
Cellular permeability	4.96 × 10^−4^	cm/min	Calculated	CDS	[49]	Permeability into the cellular space
Intestinal permeability	1.24 × 10^−2^	cm/min	Optimized	1.36 × 10^−6^	Calculated	Transcellular intestinal permeability
Formulation	Weibull °	-	Optimized	-	-	Formulation used in predictions

* if half maximal inhibitory concentrations (*IC*_50_) were reported, *K*_i_ values were calculated using the Cheng–Prusoff equation [50], and then the mean *K*_i_ was used in the model; ° Weibull function with a dissolution time of 53.47, 94.86, 71.83, or 52.59 min (50% dissolved) and a dissolution shape of 0.91, 0.91, 0.89, or 1.00 (all optimized) for oral suspension fasted [51,52], oral suspension fed [53], for capsule fasted [51], and tablet fasted [33,54,55,56,57], respectively. Berezhkovskiy, Berezhkovskiy calculation method; CDS, charge-dependent Schmitt calculation method; *CL*_hep_, hepatic metabolic clearance; conc., concentration; CYP, cytochrome P450; EHC, enterohepatic circulation; GFR, glomerular filtration rate; MATE, multidrug and toxin extrusion protein; OCT, organic cation transporter; P-gp, P-glycoprotein.

## Supplementary Materials

The following are available online at https://www.mdpi.com/1999-4923/12/11/1074/s1, Supplementary Materials: Comprehensive reference manual, providing documentation of the complete model performance assessment. Table S1: Clinical studies of trimethoprim; Table S2: Drug-dependent parameters of the final trimethoprim PBPK model; Table S3: MRD values of trimethoprim plasma (or whole blood) concentration predictions; Table S4: Predicted and observed trimethoprim fractions excreted unchanged in urine; Table S5: Predicted and observed trimethoprim *AUC*_last_ and *C*_max_ values; Table S6: Parameters evaluated during trimethoprim sensitivity analysis; Table S7: Clinical studies investigating the trimethoprim-metformin DDI and DDGI; Table S8: Drug-dependent parameters of the metformin PBPK model; Table S9: Predicted and observed trimethoprim-metformin DDI and DDGI *AUC*_last_ and *C*_max_ ratios; Table S10: Clinical studies investigating the trimethoprim-repaglinide DDI; Table S11: Drug-dependent parameters of the repaglinide PBPK model; Table S12: Predicted and observed trimethoprim-repaglinide DDI *AUC*_last_ and *C*_max_ ratios; Table S13: Clinical studies investigating the trimethoprim-pioglitazone DDI and DDGI; Table S14: Drug-dependent parameters of the pioglitazone PBPK model; Table S15: Predicted and observed trimethoprim-pioglitazone DDI and DDGI *AUC*_last_ and *C*_max_ ratios; Table S16: Clinical studies investigating the rifampicin-trimethoprim DDI; Table S17: Drug-dependent parameters of the rifampicin PBPK model; Table S18: Predicted and observed rifampicin-trimethoprim DDI *AUC*_last_ and *C*_max_ ratios; Table S19: System-dependent parameters; Figure S1: Trimethoprim DDI network; Figure S2: Comparison of trimethoprim administered alone or together with sulfamethoxazole as “cotrimoxazole”; Figure S3: Schematic illustration of the trimethoprim ADME processes in the model; Figure S4: Trimethoprim plasma concentration-time profiles (semilogarithmic); Figure S5: Trimethoprim plasma concentration-time profiles after “cotrimoxazole” administration (semilogarithmic); Figure S6: Trimethoprim plasma concentration-time profiles (linear); Figure S7: Trimethoprim plasma concentration-time profiles after “cotrimoxazole” administration (linear); Figure S8: Trimethoprim fraction excreted unchanged in urine profiles; Figure S9: Trimethoprim fraction excreted unchanged in urine profiles after “cotrimoxazole” administration; Figure S10: Trimethoprim predicted compared to observed plasma concentration values; Figure S11: Trimethoprim predicted compared to observed fractions excreted unchanged in urine; Figure S12: Trimethoprim predicted compared to observed *AUC*_last_ and *C*_max_ values; Figure S13: Trimethoprim sensitivity analysis; Figure S14: Trimethoprim-metformin DDI model processes; Figure S15: Metformin plasma concentration-time profiles before and during trimethoprim DDI and DDGI (semilogarithmic); Figure S16: Metformin plasma concentration-time profiles before and during trimethoprim DDI and DDGI (linear); Figure S17: Metformin predicted compared to observed DDI and DDGI *AUC*_last_ and *C*_max_ ratios; Figure S18: Trimethoprim-repaglinide DDI model processes; Figure S19: Repaglinide plasma concentration-time profiles before and during trimethoprim DDI; Figure S20: Repaglinide predicted compared to observed DDI *AUC*_last_ and *C*_max_ ratios; Figure S21: Trimethoprim-pioglitazone DDI model processes; Figure S22: Pioglitazone plasma concentration-time profiles before and during trimethoprim DDI and DDGI (semilogarithmic); Figure S23: Pioglitazone plasma concentration-time profiles before and during trimethoprim DDI and DDGI (linear); Figure S24: Pioglitazone predicted compared to observed DDI and DDGI *AUC*_last_ and *C*_max_ ratios; Figure S25: Rifampicin-trimethoprim DDI model processes; Figure S26: Trimethoprim and rifampicin plasma concentration-time profiles of the rifampicin DDI (semilogarithmic); Figure S27: Trimethoprim and rifampicin plasma concentration-time profiles of the rifampicin DDI (linear); Figure S28: Trimethoprim predicted compared to observed DDI *AUC*_last_ and *C*_max_ ratios.
