# Supplementary material for: A Physiologically-Based Pharmacokinetic Model of Trimethoprim for MATE1, OCT1, OCT2, and CYP2C8 Drug–Drug–Gene Interaction Predictions
Source: Pharmaceutics. 2020 Nov 10;12(11):1074. doi: 10.3390/pharmaceutics12111074 (PMC7696733; doi:10.3390/pharmaceutics12111074)
Supplement: Supplementary file 1 [file pharmaceutics-12-01074-s001.pdf]

pharmaceutics

A Physiologically-Based Pharmacokinetic Model of Trimethoprim for MATE1, OCT1, OCT2 and CYP2C8 Drug-Drug-Gene Interaction Predictions

Supplementary Materials

Denise Türk [1], Nina Hanke [1] and Thorsten Lehr [1]

[1] Clinical Pharmacy, Saarland University, Saarbrücken, Germany

Funding:
This project was partly funded by the German Federal Ministry of Education and Research (BMBF), grant number 031L0161C (“OSMOSES”). The APC was funded by the German Research Foundation (DFG) and Saarland University within the funding program “Open Access Publishing”.

Conflict of Interest:
Thorsten Lehr has received funding from the German Federal Ministry of Education and Research (grant 031L0161C). Denise Türk and Nina Hanke declare no conflict of interest. The funders had no role in the design of the study; in the collection, analyses, or interpretation of data; in the writing of the manuscript, or in the decision to publish the results.

Corresponding Author:
Prof. Dr. Thorsten Lehr
Clinical Pharmacy, Saarland University, Campus C2 2, 66123 Saarbrücken
ORCID: 0000 0002 8372 1465
Phone: +49 681 302 70255
Email: thorsten.lehr@mx.uni-saarland.de

Contents

1 Physiologically-based pharmacokinetic (PBPK) modeling 4

1.1 PBPK model building . . . 4
1.2 Drug-gene interaction (DGI) modeling . . . 4
1.3 Virtual individuals and populations . . . 5
1.3.1 Virtual individuals . . . 5
1.3.2 Virtual populations . . . 5
1.4 PBPK model evaluation . . . 6
1.4.1 Quantitative PBPK model evaluation . . . 6
1.4.2 PBPK model sensitivity analysis . . . 6
1.5 Drug-drug(-gene) interaction (DD(G)I) modeling . . . 8
1.5.1 Mathematical implementation of drug-drug interactions (DDIs) . . . 8
1.5.2 DDI modeling . . . 8
1.5.3 Drug-drug-gene interaction (DDGI) modeling . . . 9
1.5.4 DD(G)I model performance evaluation . . . 10

2 PBPK modeling of trimethoprim 11

2.1 Trimethoprim PBPK modeling . . . 11
2.2 Clinical studies . . . 14
2.3 Trimethoprim drug-dependent parameters . . . 16
2.4 Profiles . . . 17
2.4.1 Semilogarithmic plots - Plasma and whole blood . . . 17
2.4.2 Linear plots - Plasma and whole blood . . . 26
2.4.3 Linear plots - Fraction excreted unchanged in urine . . . 35
2.5 Trimethoprim PBPK model evaluation . . . 40
2.5.1 Plasma and whole blood goodness-of-fit plot . . . 40
2.5.2 MRD of plasma and whole blood predictions . . . 41
2.5.3 Fraction excreted unchanged in urine goodness-of-fit plot . . . 43
2.5.4 Predicted and observed fractions excreted unchanged in urine with mean GMFE values and ranges . . . 44
2.5.5 AUC_{last} and C_{max} goodness-of-fit plots . . . 45
2.5.6 Predicted and observed AUC_{last} and C_{max} values with mean GMFE values and ranges . . . 46
2.5.7 Sensitivity analysis . . . 48

3 Trimethoprim-metformin DDI and DDGI 50

3.1 DDI and DDGI modeling . . . 50
3.2 Clinical studies . . . 51
3.3 Metformin drug-dependent parameters . . . 52
3.4 Profiles . . . 53
3.4.1 Semilogarithmic plots - Plasma . . . 53
3.4.2 Linear plots - Plasma . . . 54
3.5 DD(G)I model performance evaluation . . . 55
3.5.1 DDI and DDGI AUC_{last} and C_{max} ratio goodness-of-fit plots . . . 55
3.5.2 Predicted and observed DDI and DDGI AUC_{last} and C_{max} ratios with mean GMFE values and ranges . . . 56

4 Trimethoprim-repaglinide DDI **57**
4.1 DDI modeling . . . 57
4.2 Clinical studies . . . 58
4.3 Repaglinide drug-dependent parameters . . . 59
4.4 Profiles . . . 60
4.4.1 Semilogarithmic and linear plots - Plasma . . . 60
4.5 DDI model performance evaluation . . . 61
4.5.1 DDI AUC_{last} and C_{max} ratio goodness-of-fit plots . . . 61
4.5.2 Predicted and observed DDI AUC_{last} and C_{max} ratios with GMFE values . . . 62

5 Trimethoprim-pioglitazone DDI and DDGI **63**
5.1 DDI and DDGI modeling . . . 63
5.2 Clinical studies . . . 64
5.3 Pioglitazone drug-dependent parameters . . . 65
5.4 Profiles . . . 66
5.4.1 Semilogarithmic plots - Plasma . . . 66
5.4.2 Linear plots - Plasma . . . 67
5.5 DD(G)I model performance evaluation . . . 68
5.5.1 DDI and DDGI AUC_{last} and C_{max} ratio goodness-of-fit plots . . . 68
5.5.2 Predicted and observed DDI and DDGI AUC_{last} and C_{max} ratios with mean GMFE values and ranges . . . 69

6 Rifampicin-trimethoprim DDI **70**
6.1 DDI modeling . . . 70
6.2 Clinical studies . . . 71
6.3 Rifampicin drug-dependent parameters . . . 72
6.4 Profiles . . . 73
6.4.1 Semilogarithmic plots - Plasma . . . 73
6.4.2 Linear plots - Plasma . . . 74
6.5 DDI model performance evaluation . . . 75
6.5.1 DDI AUC_{last} and C_{max} ratio goodness-of-fit plots . . . 75
6.5.2 Predicted and observed DDI AUC_{last} and C_{max} ratios with GMFE values . . . 76

7 System-dependent parameters **77**

List of Tables **78**

List of Figures **79**

Abbreviations **80**

References **83**

1 Physiologically-based pharmacokinetic (PBPK) modeling

1.1 PBPK model building

In this study, a PBPK model of trimethoprim was developed. PBPK model building was started with an extensive literature search to collect physicochemical parameters, information on absorption, distribution, metabolism and excretion (ADME), as well as clinical studies of intravenous and oral administration in single- and multiple-dose regimens. In addition to drug plasma or whole blood concentration-time profiles, further clinical data on fration excreted unchanged (fe) in urine were integrated. The concentration-time profiles and other collected data of the clinical studies were digitized and susbequently divided into a training dataset for model building and a test dataset for model evaluation. The studies for the training dataset were selected to include intravenous and oral administration, covering the whole published dosing range, single and multiple administration, as well as information on fe in urine. Studies were preferred for the training dataset if they were conducted with many participants, modern bioanalytical methods and frequent as well as late sampling. Model input parameters that could not be informed from literature were optimized by fitting the model simulations of all studies assigned to the training dataset simultaneously to their respective observed data.

All clinical studies used for model development are listed in Table S1, including information about the assignment of each study to the training or test dataset. Parameters of the final model are given in Table S2.

1.2 Drug-gene interaction (DGI) modeling

Solute carrier family member (SLC) 22A2 polymorphism

The *SLC22A2* gene encodes for the organic cation transporter (OCT) 2, which is mainly located at the basolateral membrane of renal cells. The *SLC22A2 808G>T* allele leads to substitution of an amino acid (Ser270Ala) caused by a single nucleotide polymorphism (SNP) (G808T, rs316019) in exon 4 [1]. Several studies show decreased maximum plasma concentrations (C_{max}) of about 13–20% in hetero- and homocygous *SLC22A2 808T* allele compared to wildtype carriers, suggesting an increased activity of the polymorphic transporter [2–5].

In the metformin DGI model, polymorphic OCT2 was implemented as two transporters with halved reference concentration each (see Table S19) and optimized transport rate constant (k_{cat}) values for both alleles (2.67-fold higher k_{cat} value in *SLC22A2 808T* allele carries [6], see Table S8).

Cytochrome P450 (CYP) 2C8 polymorphism

The *CYP2C8*3* allele is characterized by substitution of two amino acids (Arg139Lys, Lys399Arg) caused by two SNPs (G416A, A1196G) in exon 3 and exon 8, respectively [7, 8]. Carriers of the CYP2C8*3 allele (either heterozygous or homozygous) show lower plasma concentrations and area under the concentration-time curve (AUC) values of pioglitazone than *CYP2C8*1*1* (wildtype) carriers [9, 10], consistent with increased drug metabolism by CYP2C8 in vivo.

In the pioglitazone DGI model, polymorphic CYP2C8 was implemented as two enzymes with halved reference concentration each (see Table S19). Different Michaelis-Menten constant (K_M) values for CYP2C8*1*1 and CYP2C8*3*3 (measured in human liver microsomes [11]) are reported in the literature and thus used in the model, with optimized catalytic rate constant (k_{cat}) values for both alleles (see Table S14).

1.3 Virtual individuals and populations

1.3.1 Virtual individuals

Virtual mean individuals were generated for each study according to the published demographic information, with corresponding ethnicity, sex, age, body weight, height and glomerular filtration rate (GFR), if available. If no information was provided, a European, male, 30-year-old individual with mean body weight, height and GFR characteristics from the PK-Sim® population database was used. Transporters and metabolizing enzymes relevant to the pharmacokinetics of the modeled drugs were implemented in agreement with current literature, utilizing the PK-Sim® expression database [12] to define their relative expression in the different organs of the body. The system-dependent parameters for all models, including reference concentrations, tissue expression profiles as well as protein half-lives in liver and intestine of all implemented transporters and enzymes, are given in Table S19. In all virtual individuals, enterohepatic circulation (EHC) was enabled (EHC continuous fraction set to 1) by assuming a continuous flow of the bile to the duodenum.

1.3.2 Virtual populations

To cover the variability in a population, virtual populations containing 100 individuals each were created, with ethnicity, sex composition and age range adapted to each respective study protocol. If no information on ethnicity or sex was available, a European population, 100% male and 20–50 years of age was assumed. In the generated virtual populations, system-dependent parameters such as weight, height, organ volumes, blood flow rates, tissue compositions, etc. were varied by an implemented algorithm in PK-Sim® within the limits of the ICRP, NHANES or Tanaka databases [13–15]. The reference concentrations of the implemented transporters and enzymes were log-normally distributed according to the variability reported in the PK-Sim® ontogeny database [12] or in the literature. If no information could be found, reference concentrations were distributed with a moderate variability of 35% CV (geometric standard deviation of 1.4), see Table S19.

1.4 PBPK model evaluation

Model performance was evaluated with multiple methods. Predicted population plasma (or whole blood) concentration-time profiles and fe in urine profiles were compared with the data observed in the respective clinical studies. As the clinical data from literature is mostly reported as arithmetic means ± SD, population prediction arithmetic means and 68% prediction intervals were plotted, that correspond to the range of ± 1 SD around the mean if normal distribution is assumed.

Plots showing predicted plasma (or whole blood) concentration-time profiles of virtual populations compared to observed data are presented in semilogarithmic (Figures S4 and S5) and linear plots (Figures S6 and S7). Furthermore, plots showing predicted fe in urine profiles of virtual populations compared to observed data are presented in Figures S8 and S9 (linear). A goodness-of-fit plot to compare all predicted to their respective observed plasma (or whole blood) concentrations is shown in Figure S10. Additionally, the trimethoprim model performance was evaluated using goodness-of-fit plots of predicted to observed fe in urine, AUC_{last} and C_{max} values (see Figures S11 and S12).

1.4.1 Quantitative PBPK model evaluation

As quantitative performance measures, the mean relative deviation (MRD) of the predicted plasma (or whole blood) concentrations was calculated according to Equation S1 and the geometric mean fold errors (GMFEs) of fe in urine, AUC_{last} and C_{max} values were calculated according to Equation S2.

$$MRD = 10^x; \qquad x = \sqrt{\frac{1}{k}\sum_{i=1}^{k}(\log_{10} c_{predicted,i} - \log_{10} c_{observed,i})^2} \tag{S1}$$

with $c_{predicted,i}$ = predicted plasma (or whole blood) concentration, $c_{observed,i}$ = corresponding observed plasma (or whole blood) concentration, k = number of observed values. Overall MRD values ≤ 2 were considered reasonable predictions. MRD values for all studies of the trimethoprim model are given in Table S3.

$$GMFE = 10^x; \qquad x = \frac{1}{m}\sum_{i=1}^{m}|\log_{10}(\frac{predicted\ PK\ parameter_i}{observed\ PK\ parameter_i})| \tag{S2}$$

with $predicted\ PK\ parameter_i$ = predicted fe in urine, AUC_{last} or C_{max} value, $observed\ PK\ parameter_i$ = corresponding observed fe in urine, AUC_{last} or C_{max} value, m = number of studies. Overall GMFEs of ≤ 2 were considered reasonable predictions. Tables S4 and S5 list the predicted and observed fe in urine, AUC_{last} and C_{max} values of all studies as well as GMFEs for all studies of the trimethoprim model.

1.4.2 PBPK model sensitivity analysis

Sensitivity of the final model to single parameters (local sensitivity analysis) was calculated, measured as relative change of AUC_{0-12}, C_{max} or time to maximum plasma concentration (t_{max}) at steady state using the highest recommended dose of 160 mg twice daily. Sensitivity analysis was carried out with a relative perturbation of 1000% (variation range 10.0, maximum number of 9 steps). Parameters were included into the analysis if they have been optimized, if they are associated with optimized parameters, or if they could have a strong impact due to their use in the calculation of permeabilities or partition coefficients.

Sensitivity is calculated as the ratio of the relative change of the simulated AUC, C_{max} or t_{max} to the relative variation of the tested parameter around the parameter value used in the model, according to Equation S3.

$$S = \frac{\Delta PK}{PK} \cdot \frac{p}{\Delta p} \tag{S3}$$

with S = sensitivity of the AUC, C_{max} or t_{max} to the examined model parameter, ΔPK = change of the AUC, C_{max} or t_{max}, PK = simulated AUC, C_{max} or t_{max} with the original parameter value, Δp = change of the examined parameter value, p = original parameter value. The threshold value for sensitivity was set to 0.5; this value signifies that a 100% change of the investigated parameter causes a 50% change of the predicted AUC, C_{max} or t_{max}. The results of the sensitivity analysis are presented in Figure S13.

1.5 Drug-drug(-gene) interaction (DD(G)I) modeling

1.5.1 Mathematical implementation of drug-drug interactions (DDIs)

DDI modeling - competitive inhibition

Competitive inhibition describes the reversible binding of an inhibitor to the active site of a transporter or enzyme and, as a consequence, the competition of substrate and inhibitor for binding. Competitive inhibition can be overcome by high substrate concentrations (concentration-dependency). In the case of competitive inhibition, the maximum reaction velocity (v_{max}) remains unaffected, while the K_M is increased by the inhibition ($K_{M,app}$, Equation S4). The reaction velocity (v) during co-administration of substrate and competitive inhibitor is described by Equation S5 [16]:

$$K_{M,app} = K_M \cdot \left(1 + \frac{[I]}{K_i}\right) \tag{S4}$$

$$v = \frac{v_{max} \cdot [S]}{K_{M,app} + [S]} \tag{S5}$$

with $K_{M,app}$ = Michaelis-Menten constant in the presence of inhibitor, K_M = Michaelis-Menten constant, $[I]$ = free inhibitor concentration, K_i = dissociation constant of the inhibitor-transporter/ -enzyme complex, v = reaction velocity, v_{max} = maximum reaction velocity, $[S]$ = free substrate concentration.

DDI modeling - induction

Induction of a transporter or enzyme is often mediated by activation of the transcription factor pregnane X receptor (PXR). The return to baseline activity requires the clearance of the inducer and degradation of the induced protein (time-dependency). In the case of induction, the rate of transporter or enzyme synthesis (R_{syn}) is increased ($R_{syn,app}$, Equation S6), while the degradation rate constant (k_{deg}) remains unaffected. The transporter or enzyme turnover during administration of inducer is described by Equation S7. The reaction velocity during co-administration of substrate and inducer is described by Equation S8 [16]:

$$R_{syn,app} = R_{syn} \cdot \left(1 + \frac{E_{max} \cdot [Ind]}{EC_{50} + [Ind]}\right) \tag{S6}$$

$$\frac{dE(t)}{dt} = R_{syn,app} - k_{deg} \cdot E(t) \tag{S7}$$

$$v = \frac{v_{max} \cdot [S]}{K_M + [S]} = \frac{k_{cat} \cdot E(t) \cdot [S]}{K_M + [S]} \tag{S8}$$

with $R_{syn,app}$ = rate of transporter or enzyme synthesis in the presence of inducer, R_{syn} = rate of transporter or enyzme synthesis, E_{max} = maximal induction effect in vivo, $[Ind]$ = free inducer concentration, EC_{50} = concentration for half-maximal induction in vivo, $E(t)$ = transporter or enyzme concentration, k_{deg} = transporter or enzyme degradation rate constant, v = reaction velocity, v_{max} = maximum reaction velocity, $[S]$ = free substrate concentration, K_M = Michaelis-Menten constant, k_{cat} = transport or catalytic rate constant.

1.5.2 DDI modeling

The correct prediction of the impact of a perpetrator drug on the pharmacokinetics of a victim drug indicates (1) that the perpetrator model adequately describes the drug concentrations at the site(s) of interaction and (2) that the victim drug model simulates the right amount of drug eliminated via

the affected pathway. Therefore, DDI prediction is considered a valuable means to evaluate both models (provided that the clinical DDI data was not used during model optimization).

Previously developed PBPK models of metformin, repaglinide, pioglitazone and rifampicin were taken from literature without changes [6, 17, 18], and the DDI performance of the newly established trimethoprim model was evaluated by prediction of clinical results from studies of trimethoprim administered together with these different victim and perpetrator drug models (see Figure S1).

The simulations of the trimethoprim-metformin, trimethoprim-repaglinide and trimethoprim-pioglitazone DDIs in this study are predictions, as the interaction constants were taken from in vitro literature and the studies were not used as training data for model building. Only the rifampicin-trimethoprim DDI was used for model optimization, to inform the trimethoprim model processes affected by rifampicin. Details on the modeled clinical DDI studies are provided in Tables S7, S10, S13 and S16.

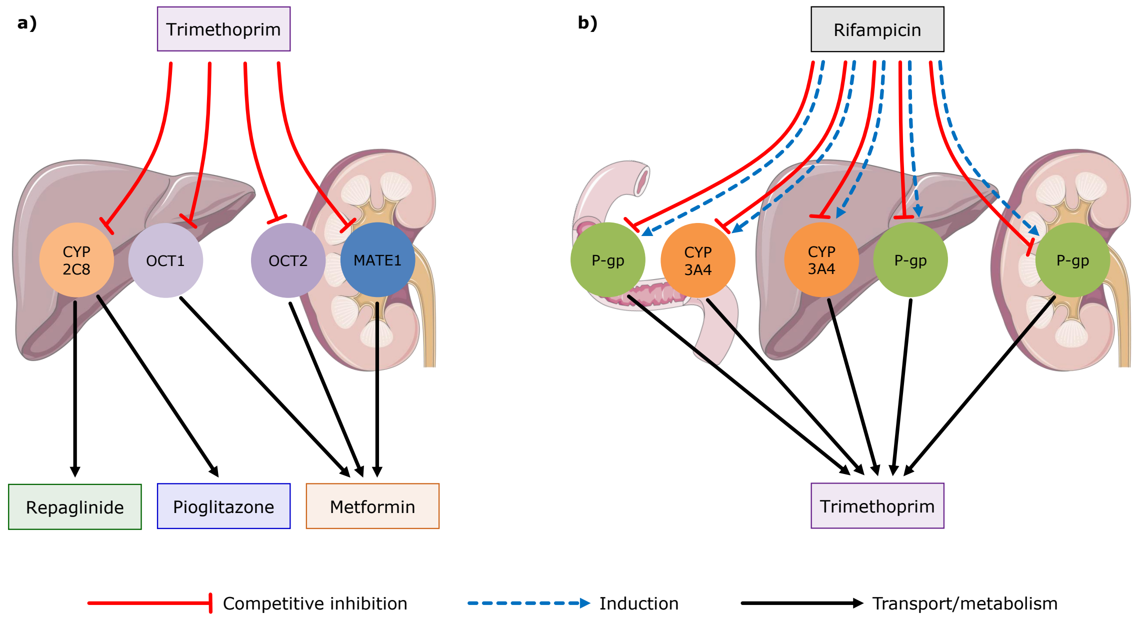

Figure S1: Trimethoprim DDI network. (**a**) Trimethoprim is a multidrug and toxin extrusion protein (MATE)1, OCT1, OCT2 and CYP2C8 inhibitor that impacts the pharmacokinetics of metformin, repaglinide and pioglitazone. (**b**) On the other hand, trimethoprim is a victim drug in the DDI with rifampicin. Rifampicin inhibits and in the long term induces P-glycoprotein (P-gp) and CYP3A4 and thereby impacts the pharmacokinetics of trimethoprim. Drawings by Servier, licensed under CC BY 3.0. *CYP* cytochrome P450, *MATE* multidrug and toxin extrusion protein, *OCT* organic cation transporter, *P-gp* P-glycoprotein.

1.5.3 Drug-drug-gene interaction (DDGI) modeling

For metfomin and pioglitazone, clinical DDGI data is available [2, 10], to test the trimethoprim DDGI model performance. The simulations of the trimethoprim-metformin and trimethoprim-pioglitazone DDGIs in this study are predictions, as the interaction constants were taken from literature and the studies were not used in the training dataset for model building. Details on the clinical studies describing the trimethoprim-metformin and trimethoprim-pioglitazone DDGIs are given in Tables S7 and S13.

1.5.4 DD(G)I model performance evaluation

Plots of population predicted plasma concentration-time profiles of the victim drugs before and during co-administration, compared to observed data, are presented in semilogarithmic (Figures S15, S19a, S22 and S26) and linear plots (Figures S16, S19b, S23 and S27). Graphical comparisons of predicted to observed DDI or DDGI AUC_{last} ratios (Equation S9) and DDI or DDGI C_{max} ratios (Equation S10) are shown in Figures S17, S20, S24 and S28.

$$\text{DDI or DDGI AUC}_{\text{last}}\text{ ratio} = \frac{\text{AUC}_{\text{last}}\text{ victim drug during perpetrator co-administration}}{\text{AUC}_{\text{last}}\text{ victim drug control}} \quad \text{(S9)}$$

$$\text{DDI or DDGI C}_{\text{max}}\text{ ratio} = \frac{\text{C}_{\text{max}}\text{ victim drug during perpetrator co-administration}}{\text{C}_{\text{max}}\text{ victim drug control}} \quad \text{(S10)}$$

As quantitative performance measures, GMFE values for all predicted DDI and DDGI AUC_{last} and C_{max} ratios were calculated according to Equation S2. The predicted and observed DDI and DDGI AUC_{last} and C_{max} ratios with GMFE values for the different interactions are listed in Tables S9, S12, S15 and S18.

2 PBPK modeling of trimethoprim

2.1 Trimethoprim PBPK modeling

Trimethoprim is an inhibitor of bacterial folic acid metabolism used to treat bacterial infections. It is either applied as a monotherapy or in combination with sulfonamides, e.g. sulfamethoxazole ("cotrimoxazole"). Trimethoprim is one of the most frequently used antibiotics worldwide, ranking fifth after penicillins, cephalosporins, macrolides and fluoroquinolones, with a global consumption of $5*10^9$ standard units in 2010 [19].

Due to the frequent prescription of trimethoprim, investigation of its DDI potential is clinically relevant. The antibiotic is a potent inhibitor of MATE1 and MATE2-K [20], and therefore recommended by the FDA as clinical MATE inhibitor. Furthermore, trimethoprim less potently inhibits OCT1 and OCT2 [21, 22]. In addition to its inhibition of transporters, trimethoprim is a weak inhibitor of CYP2C8 [20].

The trimethoprim whole-body PBPK model was built and evaluated using a total number of 66 trimethoprim plasma or whole blood concentration-time profiles and 36 fe in urine profiles (intravenous and oral, single- and multiple-dose administration), covering a broad dosing range from of 40 to 960 mg. In 47 of the 66 clinical studies, trimethoprim was administered as "cotrimoxazole", i.e. in combination with sulfamethoxazole. According to literature [23, 24] and our own analyses, trimethoprim pharmacokinetic profiles are not altered by simultaneous administration of sulfamethoxazole (see Figure S2). Consequently, studies with co-administration of trimethoprim and sulfamethoxazole were included for model development. All utilized clinical studies are listed in Table S1.

The final trimethoprim PBPK model applies active efflux via P-gp (most strongly expressed in intestine and kidney), metabolism by CYP3A4 (mainly in the liver with lower expression in the intestine), an unspecific hepatic clearance and passive glomerular filtration. Trimethoprim is primarily excreted unchanged in the urine (46–67% of an oral dose [24–26]). The implemented ADME processes are visualized in Figure S3. The drug-dependent parameters of the final model are given in Table S2. The model specific system-dependent parameters, with the expression profiles of the incorporated transporter and metabolizing enzymes, are summarized in Table S19.

The good descriptive (training dataset, 13 studies) and predictive (test dataset, 53 studies) performance of the trimethoprim model is demonstrated in semilogarithmic (Figures S4 and S5) and linear plots (Figures S6 and S7), showing population predictions of plasma or whole blood concentration-time profiles of all 66 analyzed clinical studies compared to their respective observed data. Population predictions of fe in urine values are shown in Figures S8 and S9. Furthermore, a goodness-of-fit plot with predicted versus observed plasma or whole blood concentrations is presented in Figure S10, where 93% of all predicted plasma or whole blood concentrations are within 2-fold of the observed data. A goodness-of-fit plot with fe in urine values is presented in Figure S11, where 100% of all predicted fe in urine values are within 2-fold of the observed data. MRD values for all predicted plasma or whole blood concentration-time profiles (58/66 studies with MRD ≤ 2), as well as GMFE values for predicted fe in urine values (overall GMFE of 1.19), are documented in Tables S3 and S4, respectively.

Correlation of predicted with observed AUC_{last} (97% within 2-fold) and C_{max} values (98% within 2-fold) is presented in Figure S12. The plotted values for all studies are provided in Table S5, including calculated GMFE values, with overall GMFEs of 1.29 and 1.20 for AUC_{last} and C_{max}, respectively.

Sensitivity analysis of a simulation of 160 mg trimethoprim twice daily, using a parameter perturbation of 1000% and a sensitivity threshold of 0.5, showed that the only parameter value the model predictions are sensitive to is the trimethoprim fraction unbound in plasma, for which a literature value is used in the model (56% [27]). The full quantitative results of the sensitivity analysis are shown in Section 2.5.7.

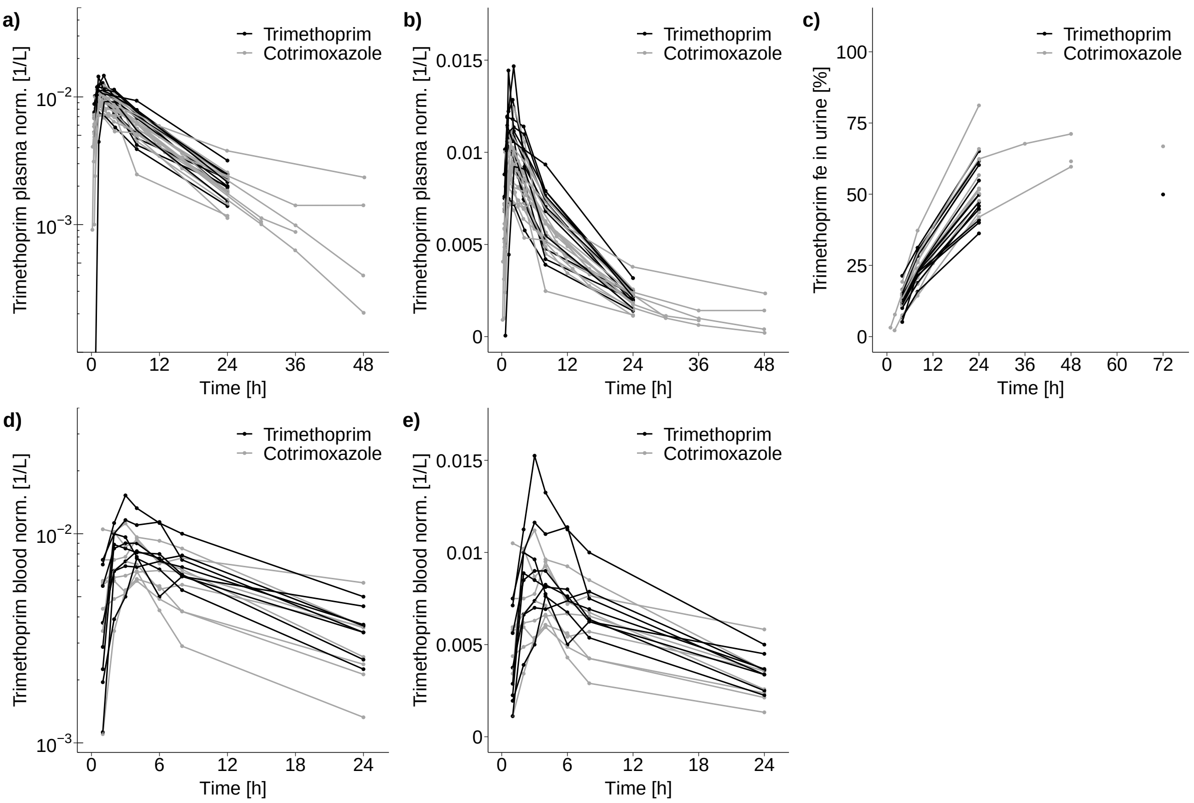

Figure S2: Plasma or whole blood concentration-time profiles of trimethoprim administered alone or together with sulfamethoxazole as "cotrimoxazole". Comparison of trimethoprim (**a-b**) dose-normalized plasma concentration-time and (**c**) fraction excreted unchanged in urine profiles of all studies, administered as a single dose tablet of either trimethoprim only or trimethoprim together with sulfamethoxazole as "cotrimoxazole". (**d-e**) Comparison of trimethoprim dose-normalized whole blood concentration-time profiles of one study by Kaplan et al. [24], where trimethoprim was administered alone or as "cotrimoxazole" in a cross-over design in the same eight individuals. *fe in urine* fraction excreted unchanged in urine.

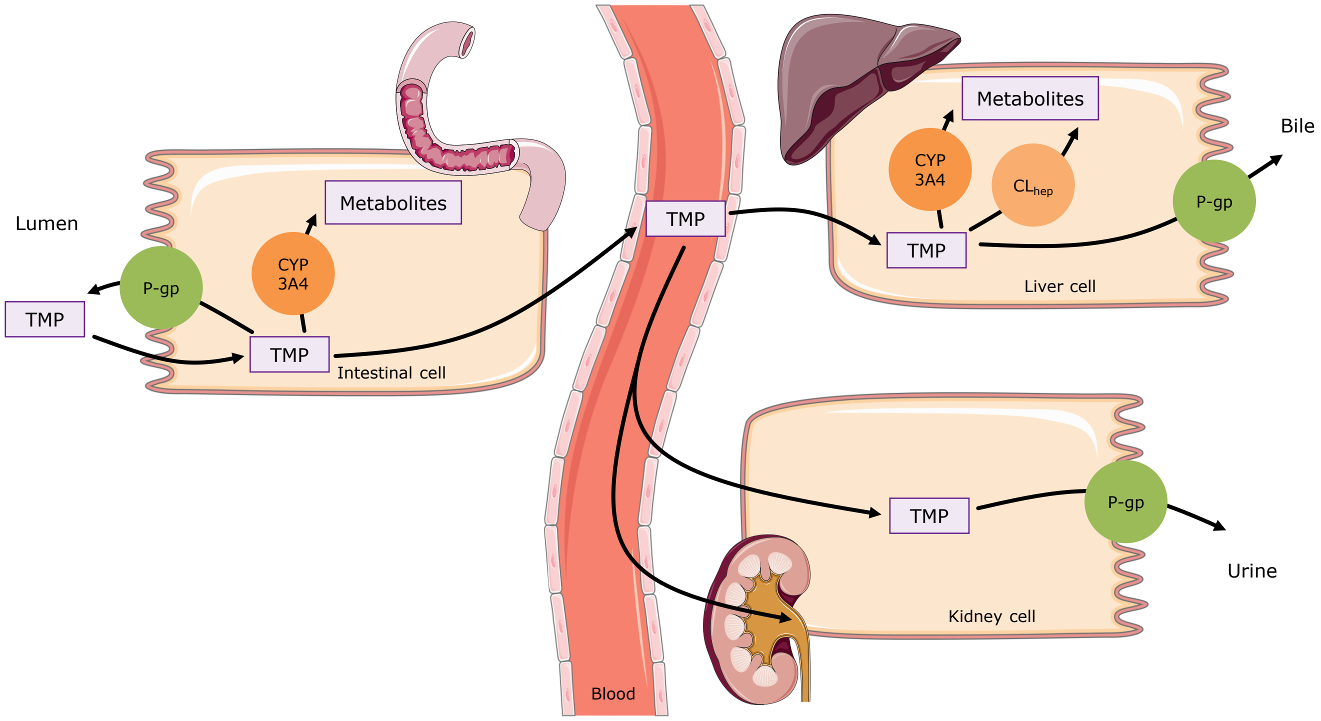

Figure S3: Schematic illustration of the trimethoprim ADME processes in the model. Trimethoprim is absorbed in the intestine with counteractive efflux via P-gp. About 20% of a trimethoprim dose are metabolized [28] (modeled via CYP3A4 and an additional hepatic metabolic clearance (CL_{hep})). The main route of trimethoprim elimination is urinary excretion (46–67% of an oral dose [24–26]) via glomerular filtration and active tubular secretion via P-gp. Drawings by Servier, licensed under CC BY 3.0. CL_{hep} hepatic metabolic clearance, *CYP* cytochrome P450, *P-gp* P-glycoprotein, *TMP* trimethoprim.

2.2 Clinical studies

The clinical studies used for trimethoprim PBPK model development are summarized in Table S1.

Table S1: Clinical studies of trimethoprim

Route	Dose [mg]	n	Females [%]	Age [years]	Weight [kg]	Height [cm]	Dataset	Reference
Trimethoprim								
po (tab, sd)	100	18	33	-	(73)	-	training	Bach 1973 [a] [29]
po (tab, sd)	100	18	33	-	(73)	-	test	Bach 1973 [b] [29]
po (tab, sd)	100	18	33	-	(73)	-	test	Bach 1973 [c] [29]
po (tab, sd)	100	1	0	-	64.5	-	test	Bach 1973 [29]
po (tab, sd)	100	1	100	-	61.4	-	test	Bach 1973 [29]
po (tab, sd)	100	1	0	-	76.4	-	test	Bach 1973 [29]
po (-, sd)	100	1	-	-	-	-	test	Weinfeld 1979 [25]
po (-, sd)	160	6	0	24-27	47-65	-	test	Guptat 1991 [26]
po (tab, sd)	200	6	-	-	-	-	test	Bach 1973 [a] [29]
po (tab, sd)	200	6	-	-	-	-	test	Bach 1973 [b] [29]
po (tab, sd)	200	6	-	-	-	-	training	Bach 1973 [c] [29]
po (tab, sd)	200	1	0	-	64.5	-	test	Bach 1973 [29]
po (tab, sd)	200	1	100	-	61.4	-	test	Bach 1973 [29]
po (tab, sd)	200	1	0	-	76.4	-	test	Bach 1973 [29]
po (susp, sd)	3 /kg	12	25	27-45	-	-	training	Hoppu 1987 [30]
po (susp, sd, fed)	3 /kg	12	25	27-45	-	-	training	Hoppu 1987 [30]
po (tab, sd)	400	8	0	-	(78.7)	-	test	Kaplan 1973 [24]
po (-, sd)	400	10	45	18-48	45-94	-	training	Klimowicz 1988 [31]
po (tab, bid)	160	10	30	20-24 (22)	52-88 (69)	-	test	Niemi 2004b [32]
po (-, bid)	400/200	10	45	18-48	45-94	-	test	Klimowicz 1988 [31]
Cotrimoxazole								
iv (1 h, sd)	2 /kg	8	13	22-27 (23.8)	(77.2)	-	test	Hutabarat 1991 [33]
iv (1 h, sd)	200	6	33	22-31 (25)	57-77 (69)	168-183 (178)	training	Männistö 1982 [34]
iv (0.75 h, sd)	240	7	-	21-40	-	-	training	Spicehandler 1982 [35]
iv (0.75 h, bid)	240	7	-	21-40	-	-	training	Spicehandler 1982 [35]
po (susp, sd)	40	12	-	-	-	-	test	Ratiopharm 1988 [36]
po (susp, sd)	40	16	0	-	-	-	test	Meda 2013 [37]
po (susp, sd)	80	12	-	-	-	-	test	Ratiopharm 1988 [36]
po (tab, sd)	80	18	33	-	(73)	-	test	Bach 1973 [a] [29]

Values for age, weight and height are reported as range (mean). [a] Burroughs Wellcome Co., Inc., Research Triangle Park, NC, [b] Channing Laboratory, Boston City Hospital, MA, [c] Hoffmann-La Roche Inc., Nutley, NJ. - not given, *bid* twice daily, *caps* capsule, *iv* intravenous, *n* number of individuals studied, *po* oral, *qid* four times daily, *sd* single dose, *susp* oral suspension, *tab* tablet, *test* test dataset (model evaluation), *training* training dataset (model building).

Table S1: Clinical studies of trimethoprim *(continued)*

Route	Dose [mg]	n	Females [%]	Age [years]	Weight [kg]	Height [cm]	Dataset	Reference
po (tab, sd)	80	18	33	-	(73)	-	test	Bach 1973 [b] [29]
po (tab, sd)	80	18	33	-	(73)	-	test	Bach 1973 [c] [29]
po (tab, sd)	80	1	0	-	64.5	-	test	Bach 1973 [29]
po (tab, sd)	80	1	100	-	61.4	-	test	Bach 1973 [29]
po (tab, sd)	80	1	0	-	76.4	-	test	Bach 1973 [29]
po (tab, sd)	80	12	-	-	-	-	training	Ratiopharm 1991 [28]
po (-, sd)	80	5	-	-	-	-	test	DeAngelis 1990 [38]
po (susp, sd)	160	26	-	18-45	-	-	training	Bedor 2008 [39]
po (caps, sd)	160	26	-	18-45	-	-	training	Bedor 2008 [39]
po (tab, sd)	160	12	-	-	-	-	test	Amini 2007 [40]
po (tab, sd)	160	6	-	-	-	-	test	Bach 1973 [a] [29]
po (tab, sd)	160	6	-	-	-	-	test	Bach 1973 [b] [29]
po (tab, sd)	160	6	-	-	-	-	test	Bach 1973 [c] [29]
po (tab, sd)	160	36	-	-	-	-	test	Ratiopharm 1987 [28]
po (tab, sd)	160	10	0	20-34 (24.2)	58-87 (66.5)	166-176 (171)	test	Flores-Murrieta 1990 [41]
po (tab, sd)	160	1	-	-	-	-	test	Gochin 1981 [42]
po (tab, sd)	160	12	0	18-54	-	-	test	Mistri 2010 [43]
po (tab, sd)	160	8	100	-	52-74 (60)	-	test	Örtengren 1979 [44]
po (tab, sd)	160	6	33	26-35 (29.3)	50-75 (65)	-	test	Varoquaux 1985 [45]
po (tab, sd)	160	10	50	18-25	58-80	-	test	Watson 1982 [46]
po (-, sd)	160	1	-	-	-	-	test	Weinfeld 1979 [25]
po (-, sd)	160	1	-	-	-	-	test	Weinfeld 1979 [25]
po (-, sd)	160	1	-	-	-	-	test	Welling 1973 [47]
po (tab, sd)	320	5	40	22-27	56-78	-	test	Bruun 1981 [48]
po (-, sd)	320	1	0	50	64	170	test	Królicki 2004 [49]
po (-, sd)	320	1	0	42	68	172	test	Królicki 2004 [49]
po (-, sd)	320	1	0	52	80	170	test	Królicki 2004 [49]
po (-, sd)	320	1	0	19	70	180	test	Królicki 2004 [49]
po (tab, sd)	400	12	-	-	56-108 (74.1)	-	test	Eatman 1977 [50]
po (tab, sd)	400	24	0	-	(75.9)	-	test	Kaplan 1973 [24]
po (tab, sd)	720	7	-	24-34	-	-	test	Yoshikawa 1976 [51]
po (tab, sd)	960	15	0	18-38 (26.8)	64-98 (77.1)	-	training	Fass 1977 [52]
po (tab, bid)	160	8	100	-	52-74 (60)	-	test	Örtengren 1979 [44]
po (tab, bid)	160	10	50	18-25	58-80	-	test	Watson 1982 [46]
po (-, bid)	160	1	-	-	-	-	test	Reeves 1979 [53]
po (-, bid)	160	1	-	-	-	-	test	Reeves 1979 [53]
po (-, qid)	3 /kg	6	0	(26.7)	(73.7)	-	test	Stevens 1993 [54]
po (tab, bid)	320	5	40	22-27	56-78	-	test	Bruun 1981 [48]
po (-, qid)	5 /kg	12	0	22-32 (28.2)	61-89 (75.8)	-	training	Stevens 1991 [55]

Values for age, weight and height are reported as range (mean). [a] Burroughs Wellcome Co., Inc., Research Triangle Park, NC, [b] Channing Laboratory, Boston City Hospital, MA, [c] Hoffmann-La Roche Inc., Nutley, NJ. - not given, *bid* twice daily, *caps* capsule, *iv* intravenous, *n* number of individuals studied, *po* oral, *qid* four times daily, *sd* single dose, *susp* oral suspension, *tab* tablet, *test* test dataset (model evaluation), *training* training dataset (model building).

2.3 Trimethoprim drug-dependent parameters

Table S2: Drug-dependent parameters of the final trimethoprim PBPK model

Parameter	Value	Unit	Source	Literature	Reference	Description
Trimethoprim						
MW	290.32	g/mol	Literature	290.32	[57]	Molecular weight
pKa (base)	7.12		Literature	6.60, 7.12, 7.30	[53, 57, 58]	Acid dissociation constant
Solubility (pH 7.0)	0.40	g/L	Literature	0.40	[57]	Solubility
logP	1.01		Optimized	0.60, 0.73, 0.91, 1.43	[57, 59–61]	Lipophilicity
fu	56	%	Literature	42 – 65	[27, 33, 34, 45, 53, 62–64]	Fraction unbound plasma
P-gp K_M	195.75	µmol/L	Optimized	-	-	Michaelis-Menten constant
P-gp k_{cat}	1.44	1/min	Optimized	-	-	Transport rate constant
CYP3A4 K_M	375.57	µmol/L	Optimized	-	-	Michaelis-Menten constant
CYP3A4 k_{cat}	0.56	1/min	Optimized	-	-	Catalytic rate constant
CL_{hep}	1.61E-02	1/min	Optimized	-	-	Hepatic metabolic clearance
GFR fraction	1		Assumed	-	-	Fraction of filtered drug in the urine
EHC continuous fraction	1		Assumed	-	-	Fraction of bile continually released
MATE1 K_i	4.45	µmol/L	Literature	0.51, 2.64, 3.29, 3.94, 4.06, 4.58, 6.30, 6.73, 7.99 [a]	[21, 22, 65–67]	Conc. for 50% inhibition (competitive)
OCT1 K_i	32.20	µmol/L	Literature	27.70, 36.70 [a]	[21, 22]	Conc. for 50% inhibition (competitive)
OCT2 K_i	47.82	µmol/L	Literature	13.20, 19.80, 27.20, 32.30, 57.40, 137.00 [a]	[21, 22, 65, 68, 69]	Conc. for 50% inhibition (competitive)
CYP2C8 K_i	4.85	µmol/L	Literature	2.25, 3.80, 8.50 [a]	[70]	Conc. for 50% inhibition (competitive)
Partition coefficients	Diverse		Calculated	Berezhkovskiy	[71]	Cell to plasma partition coefficients
Cellular permeability	4.96E-04	cm/min	Calculated	CDS	[16]	Permeability into the cellular space
Intestinal permeability	1.24E-02	cm/min	Optimized	1.36E-06	Calculated	Transcellular intestinal permeability
Formulation	Weibull [b]		Optimized	-	-	Formulation used in predictions

[a] if half maximal inhibitory concentrations (IC_{50}) were reported, K_i values were calculated using the Cheng-Prusoff equation [56], and then the mean K_i was used in the model, [b] Weibull function with a dissolution time of 53.47, 94.86, 71.83 or 52.59 minutes (50% dissolved) and a dissolution shape of 0.91, 0.91, 0.89 or 1.00 (all optimized) for oral suspension fasted [36, 39], oral suspension fed [30], capsule fasted [39] and tablet fasted [28, 29, 31, 52, 55], respectively. *Berezhkovskiy* Berezhkovskiy calculation method, *CDS* charge-dependent Schmitt calculation method, CL_{hep} hepatic metabolic clearance, *conc.* concentration, *CYP* cytochrome P450, *EHC*: enterohepatic circulation, *GFR* glomerular filtration rate, *MATE* multidrug and toxin extrusion protein, *OCT* organic cation transporter, *P-gp* P-glycoprotein.

2.4 Profiles

2.4.1 Semilogarithmic plots - Plasma and whole blood

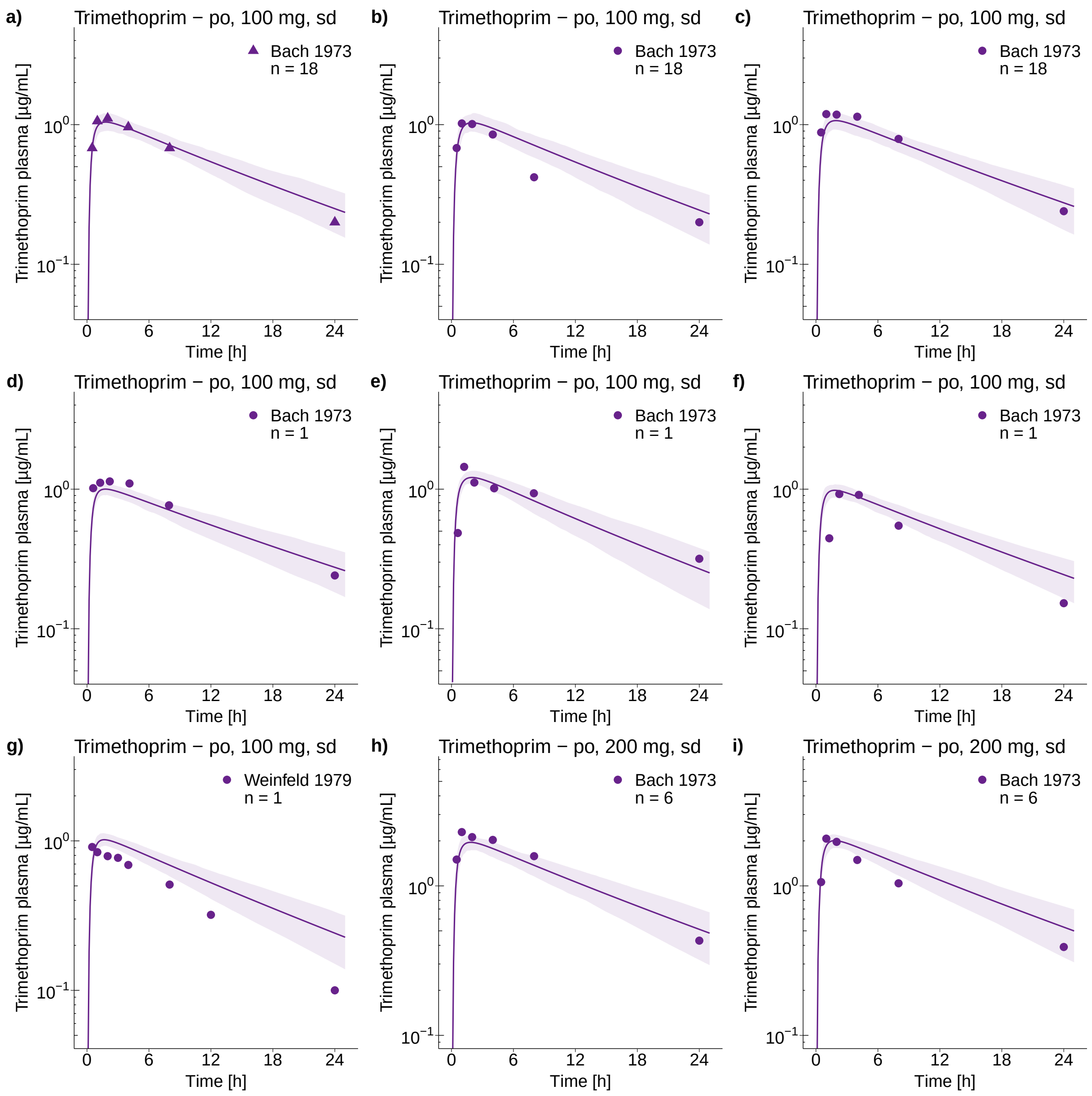

Figure S4: Trimethoprim plasma (or whole blood) concentration-time profiles (semilogarithmic). Observed data are shown as triangles (training dataset) or circles (test dataset) ± standard deviation. Population simulation arithmetic means are shown as lines; the shaded areas represent the 68% population prediction intervals. Details on dosing regimens, study populations and literature references are listed in Table S1. Predicted and observed AUC_{last} and C_{max} values are summarized in Table S5. *n* number of individuals studied, *po* oral, *sd* single dose.

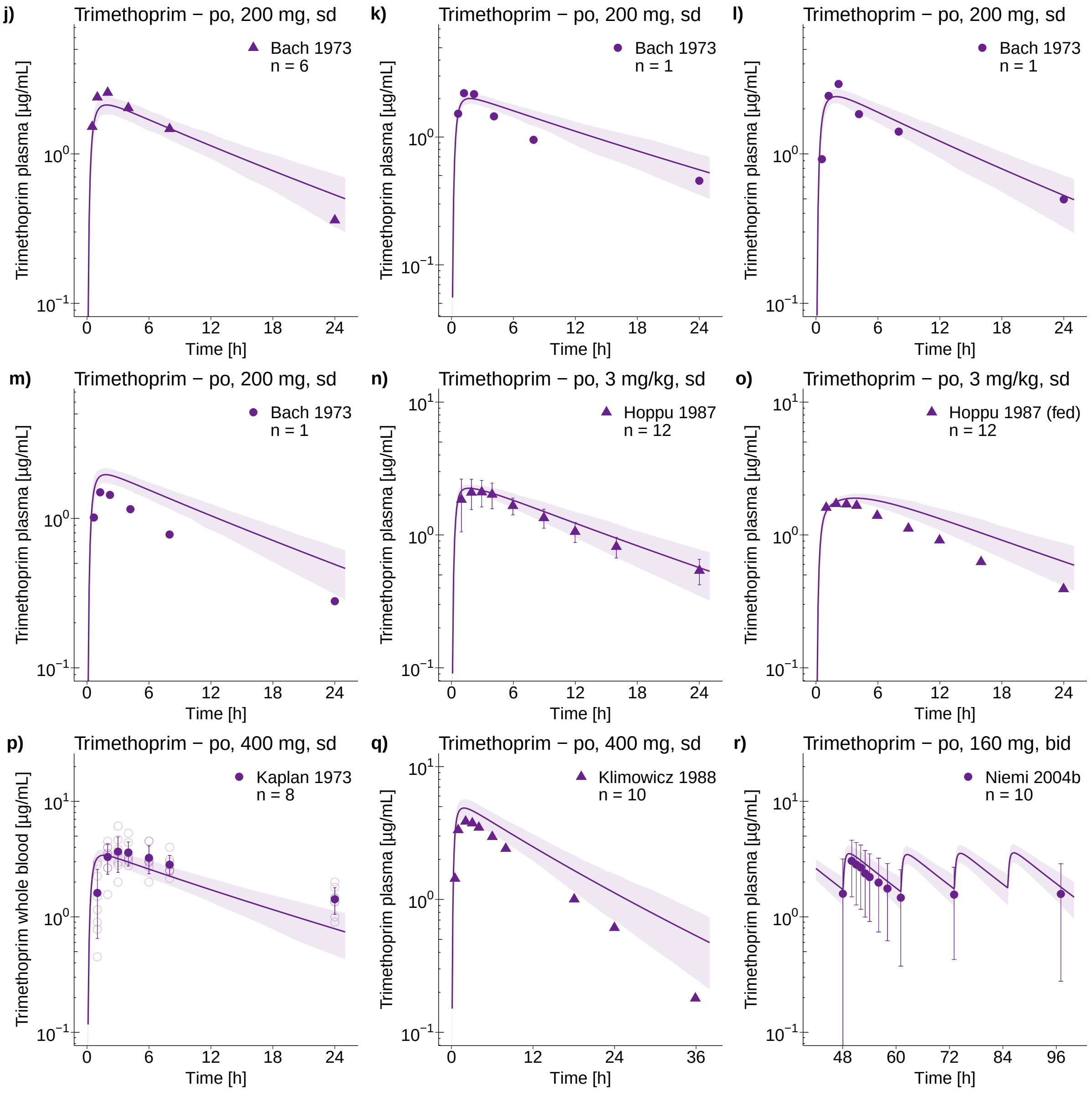

Figure S4: Trimethoprim plasma (or whole blood) concentration-time profiles (semilogarithmic). Observed data are shown as triangles (training dataset) or circles (test dataset) ± standard deviation. Population simulation arithmetic means are shown as lines; the shaded areas represent the 68% population prediction intervals. Details on dosing regimens, study populations and literature references are listed in Table S1. Predicted and observed AUC_{last} and C_{max} values are summarized in Table S5. *bid* twice daily, *n* number of individuals studied, *po* oral, *sd* single dose. *(continued)*

s)

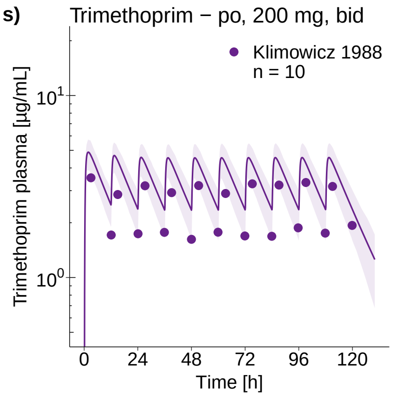

Figure S4: Trimethoprim plasma (or whole blood) concentration-time profiles (semilogarithmic). Observed data are shown as triangles (training dataset) or circles (test dataset) ± standard deviation. Population simulation arithmetic means are shown as lines; the shaded areas represent the 68% population prediction intervals. Details on dosing regimens, study populations and literature references are listed in Table S1. Predicted and observed AUC_{last} and C_{max} values are summarized in Table S5. *bid* twice daily, *n* number of individuals studied, *po* oral. *(continued)*

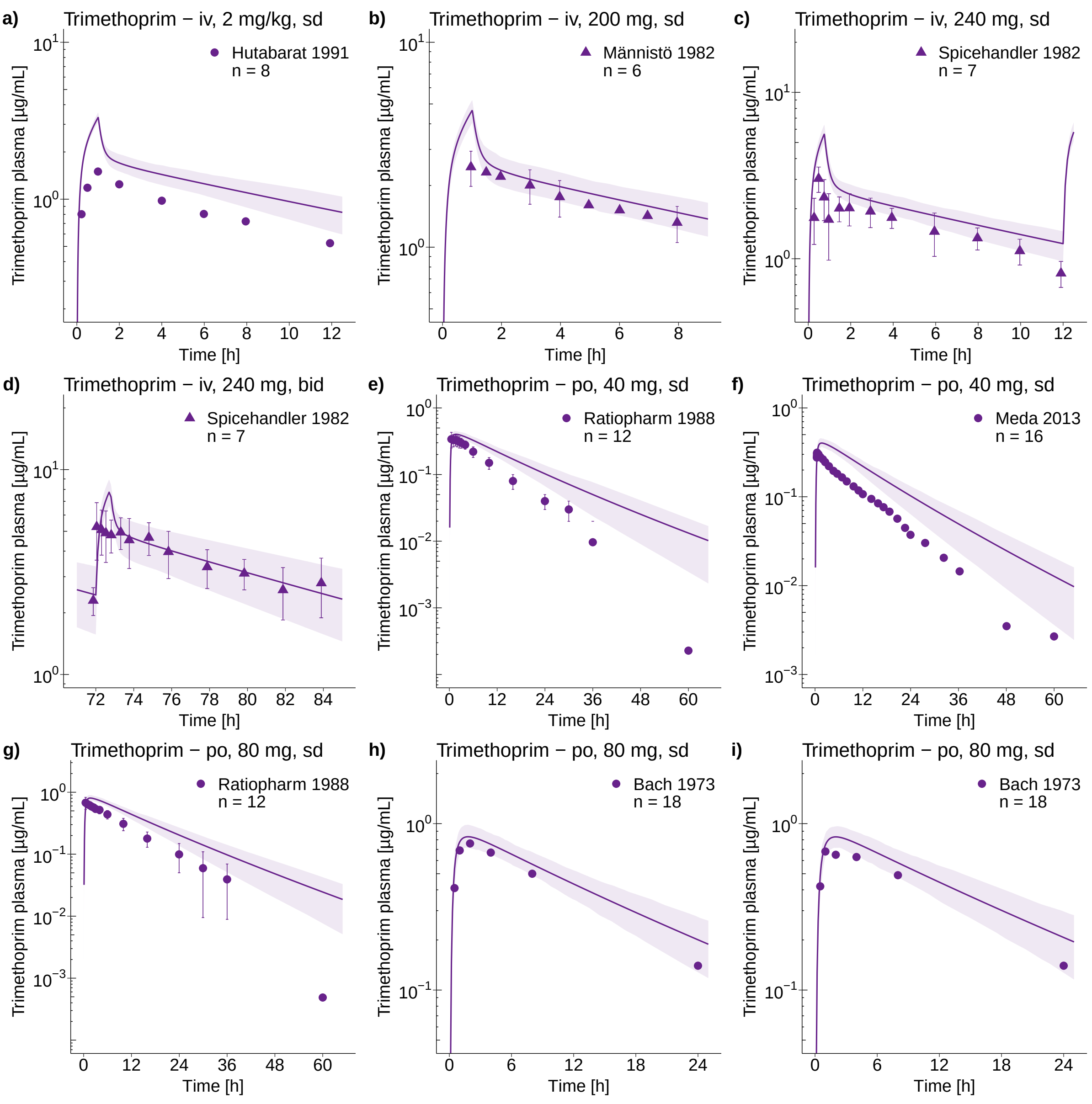

Figure S5: Trimethoprim plasma (or whole blood) concentration-time profiles after "cotrimoxazole" administration (semilogarithmic). Observed data are shown as triangles (training dataset) or circles (test dataset) ± standard deviation. Population simulation arithmetic means are shown as lines; the shaded areas represent the 68% population prediction intervals. Details on dosing regimens, study populations and literature references are listed in Table S1. Predicted and observed AUC_{last} and C_{max} values are summarized in Table S5. *iv* intravenous, *n* number of individuals studied, *po* oral, *sd* single dose.

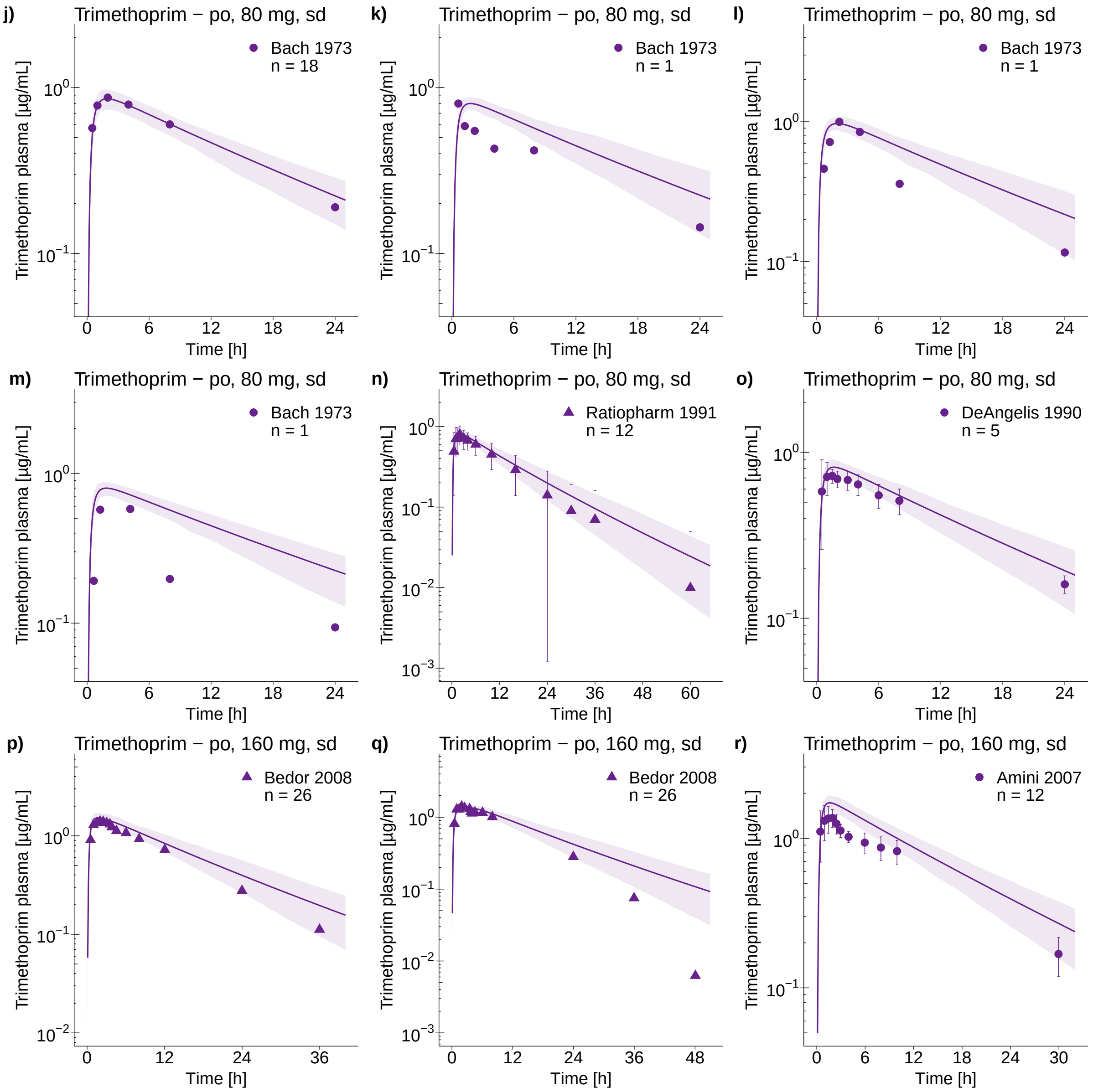

Figure S5: Trimethoprim plasma (or whole blood) concentration-time profiles after "cotrimoxazole" administration (semilogarithmic). Observed data are shown as triangles (training dataset) or circles (test dataset) ± standard deviation. Population simulation arithmetic means are shown as lines; the shaded areas represent the 68% population prediction intervals. Details on dosing regimens, study populations and literature references are listed in Table S1. Predicted and observed AUC_{last} and C_{max} values are summarized in Table S5. *n* number of individuals studied, *po* oral, *sd* single dose. *(continued)*

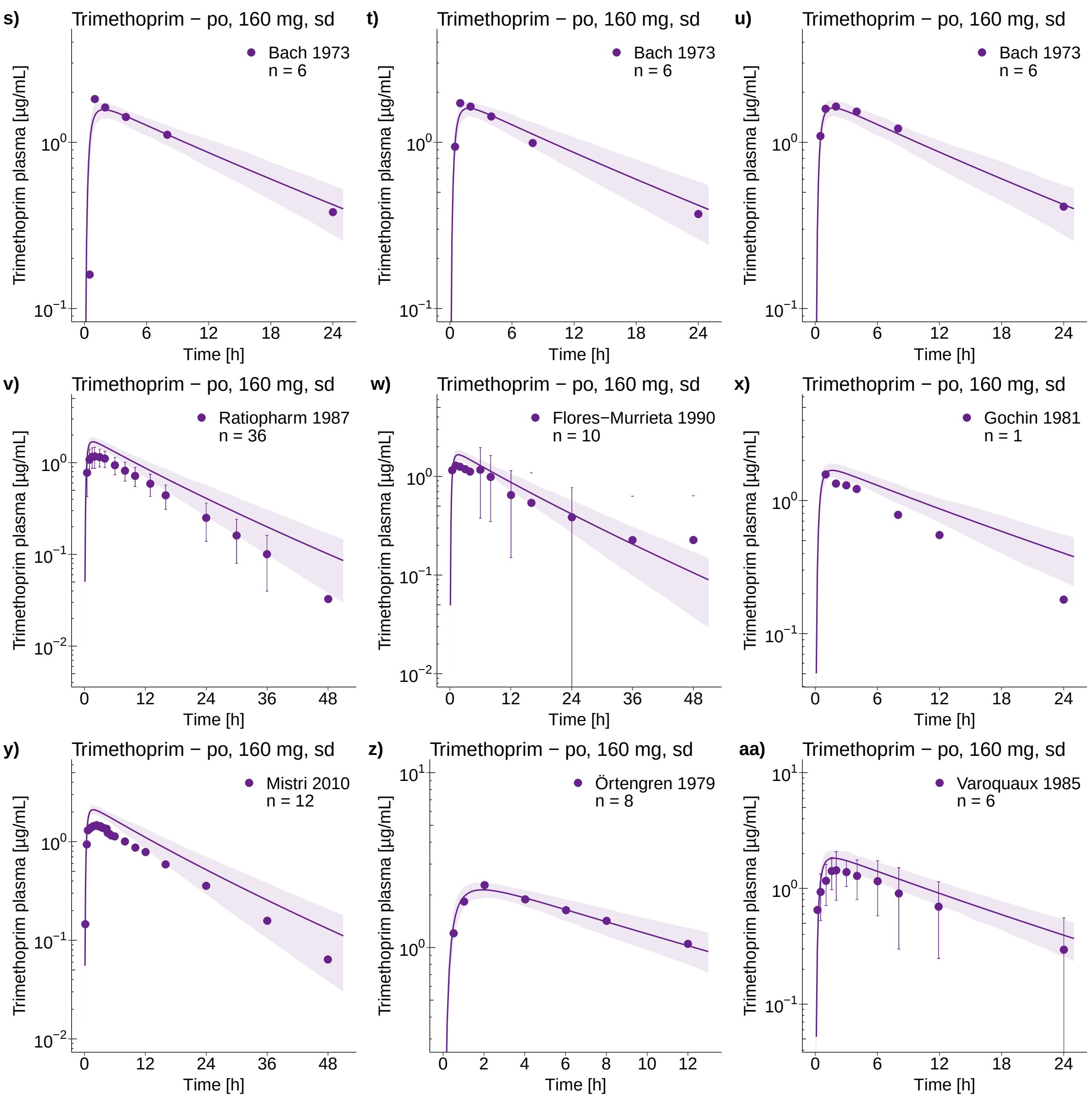

Figure S5: Trimethoprim plasma (or whole blood) concentration-time profiles after "cotrimoxazole" administration (semilogarithmic). Observed data are shown as triangles (training dataset) or circles (test dataset) ± standard deviation. Population simulation arithmetic means are shown as lines; the shaded areas represent the 68% population prediction intervals. Details on dosing regimens, study populations and literature references are listed in Table S1. Predicted and observed AUC_{last} and C_{max} values are summarized in Table S5. *n* number of individuals studied, *po* oral, *sd* single dose. *(continued)*

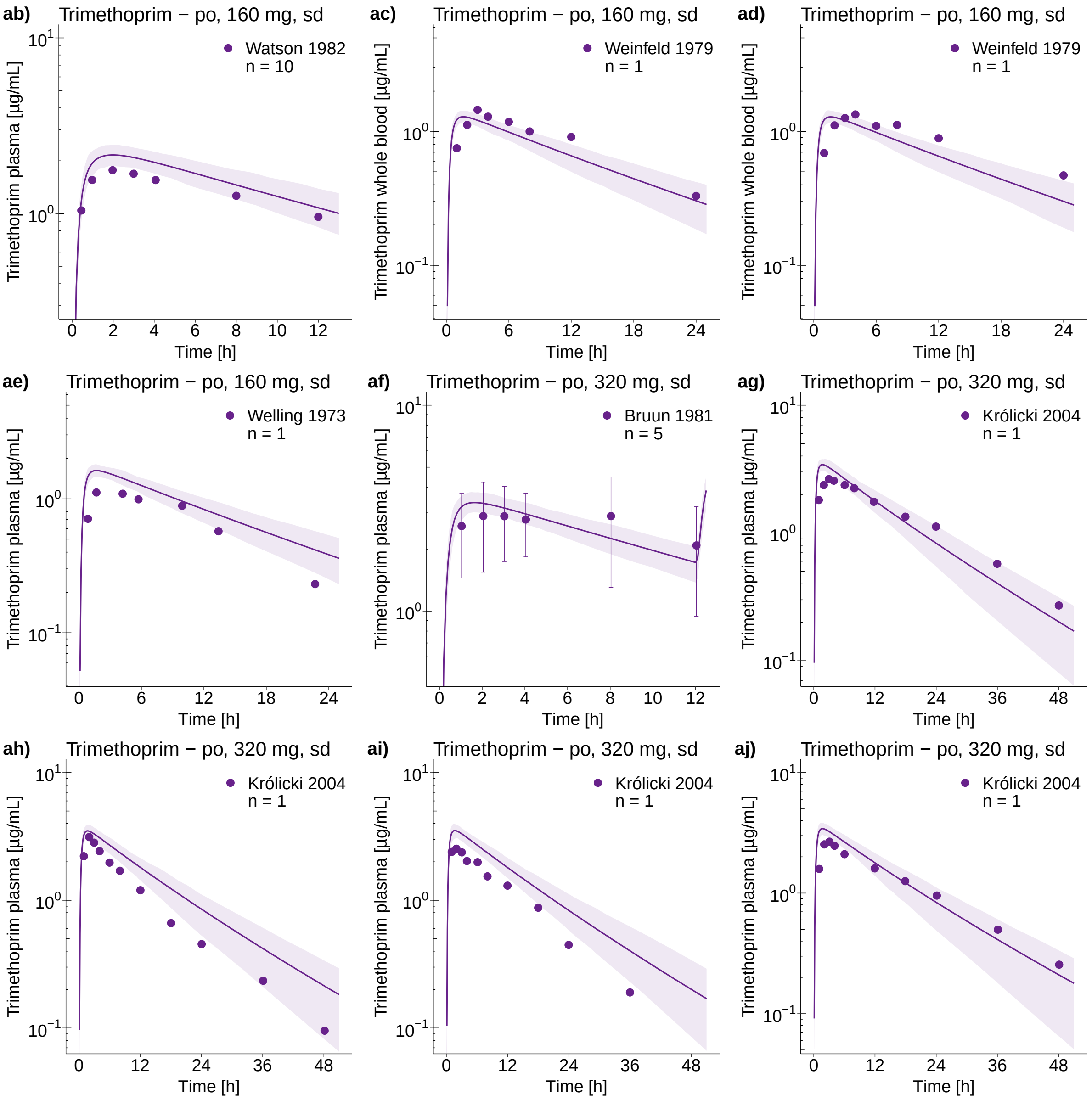

Figure S5: Trimethoprim plasma (or whole blood) concentration-time profiles after "cotrimoxazole" administration (semilogarithmic). Observed data are shown as triangles (training dataset) or circles (test dataset) ± standard deviation. Population simulation arithmetic means are shown as lines; the shaded areas represent the 68% population prediction intervals. Details on dosing regimens, study populations and literature references are listed in Table S1. Predicted and observed AUC_{last} and C_{max} values are summarized in Table S5. *n* number of individuals studied, *po* oral, *sd* single dose. *(continued)*

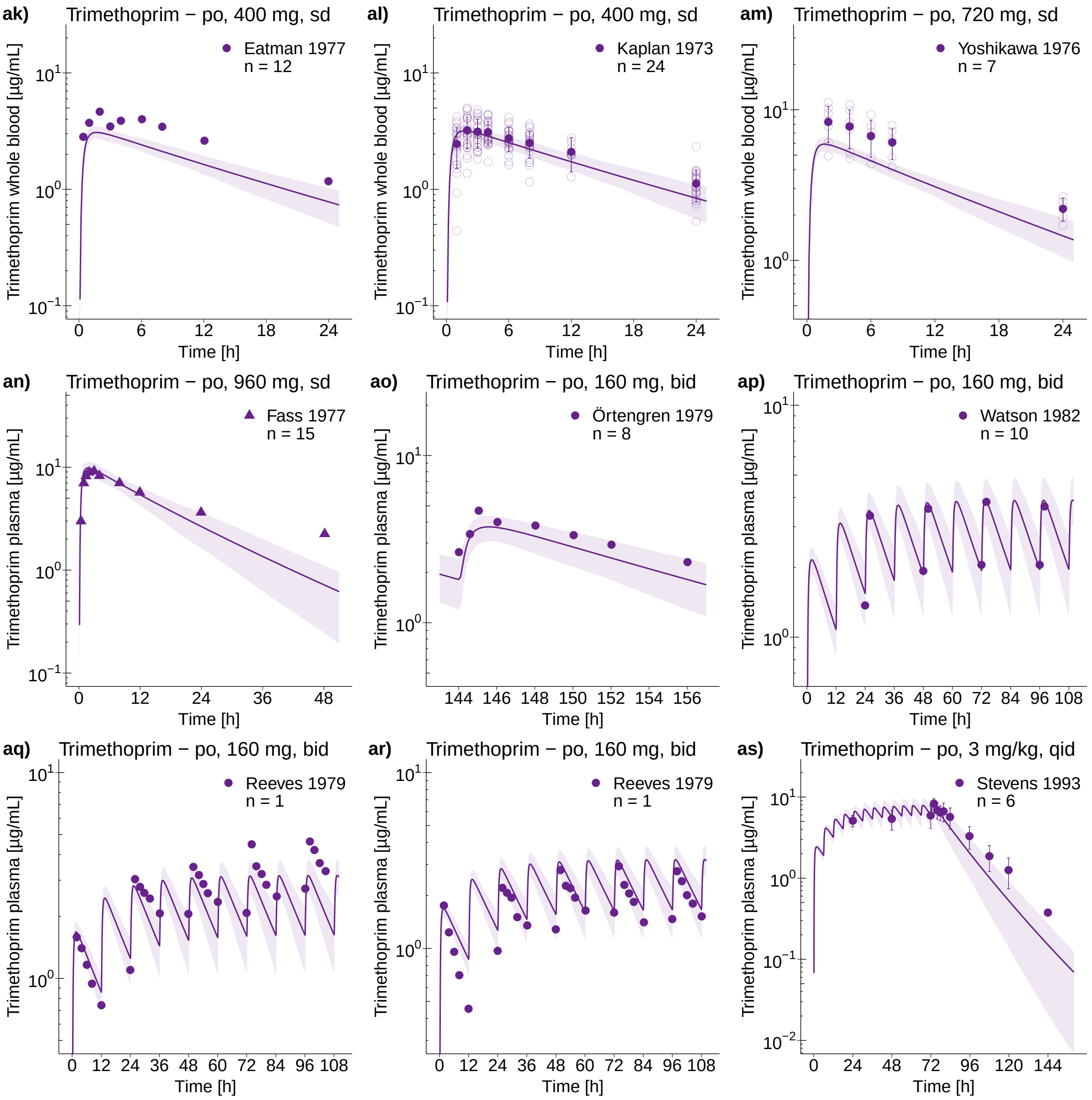

Figure S5: Trimethoprim plasma (or whole blood) concentration-time profiles after "cotrimoxazole" administration (semilogarithmic). Observed data are shown as triangles (training dataset) or circles (test dataset) ± standard deviation. Population simulation arithmetic means are shown as lines; the shaded areas represent the 68% population prediction intervals. Details on dosing regimens, study populations and literature references are listed in Table S1. Predicted and observed AUC_{last} and C_{max} values are summarized in Table S5. *bid* twice daily, *n* number of individuals studied, *po* oral, *qid* four times daily. *(continued)*

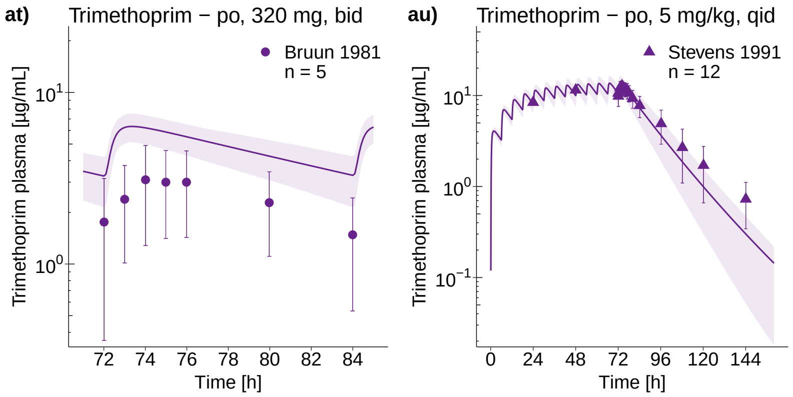

Figure S5: Trimethoprim plasma (or whole blood) concentration-time profiles after "cotrimoxazole" administration (semilogarithmic). Observed data are shown as triangles (training dataset) or circles (test dataset) ± standard deviation. Population simulation arithmetic means are shown as lines; the shaded areas represent the 68% population prediction intervals. Details on dosing regimens, study populations and literature references are listed in Table S1. Predicted and observed AUC_{last} and C_{max} values are summarized in Table S5. *bid* twice daily, *n* number of individuals studied, *po* oral, *qid* four times daily. *(continued)*

2.4.2 Linear plots - Plasma and whole blood

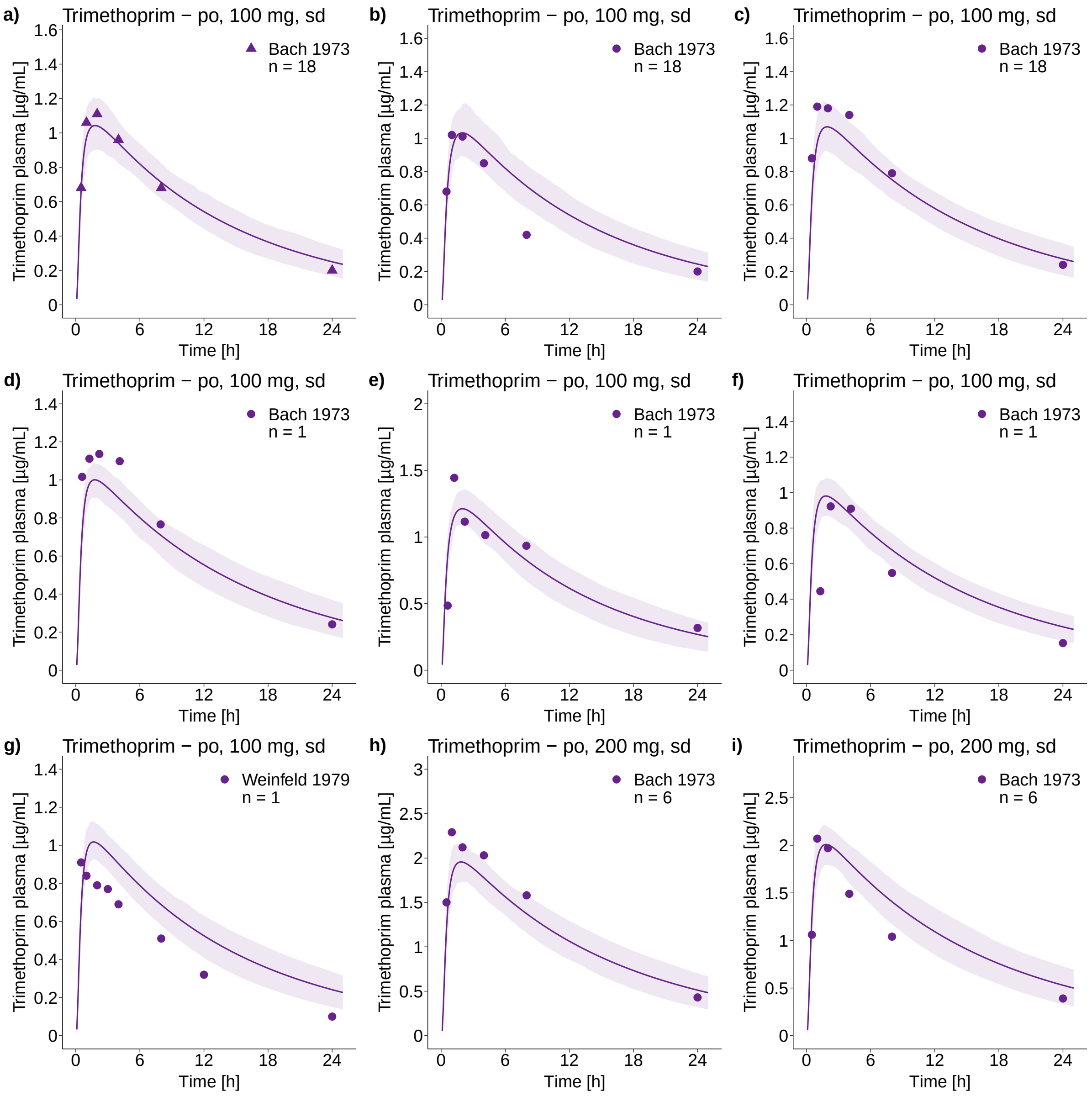

Figure S6: Trimethoprim plasma (or whole blood) concentration-time profiles (linear). Observed data are shown as triangles (training dataset) or circles (test dataset) ± standard deviation. Population simulation arithmetic means are shown as lines; the shaded areas represent the 68% population prediction intervals. Details on dosing regimens, study populations and literature references are listed in Table S1. Predicted and observed AUC_{last} and C_{max} values are summarized in Table S5. *n* number of individuals studied, *po* oral, *sd* single dose.

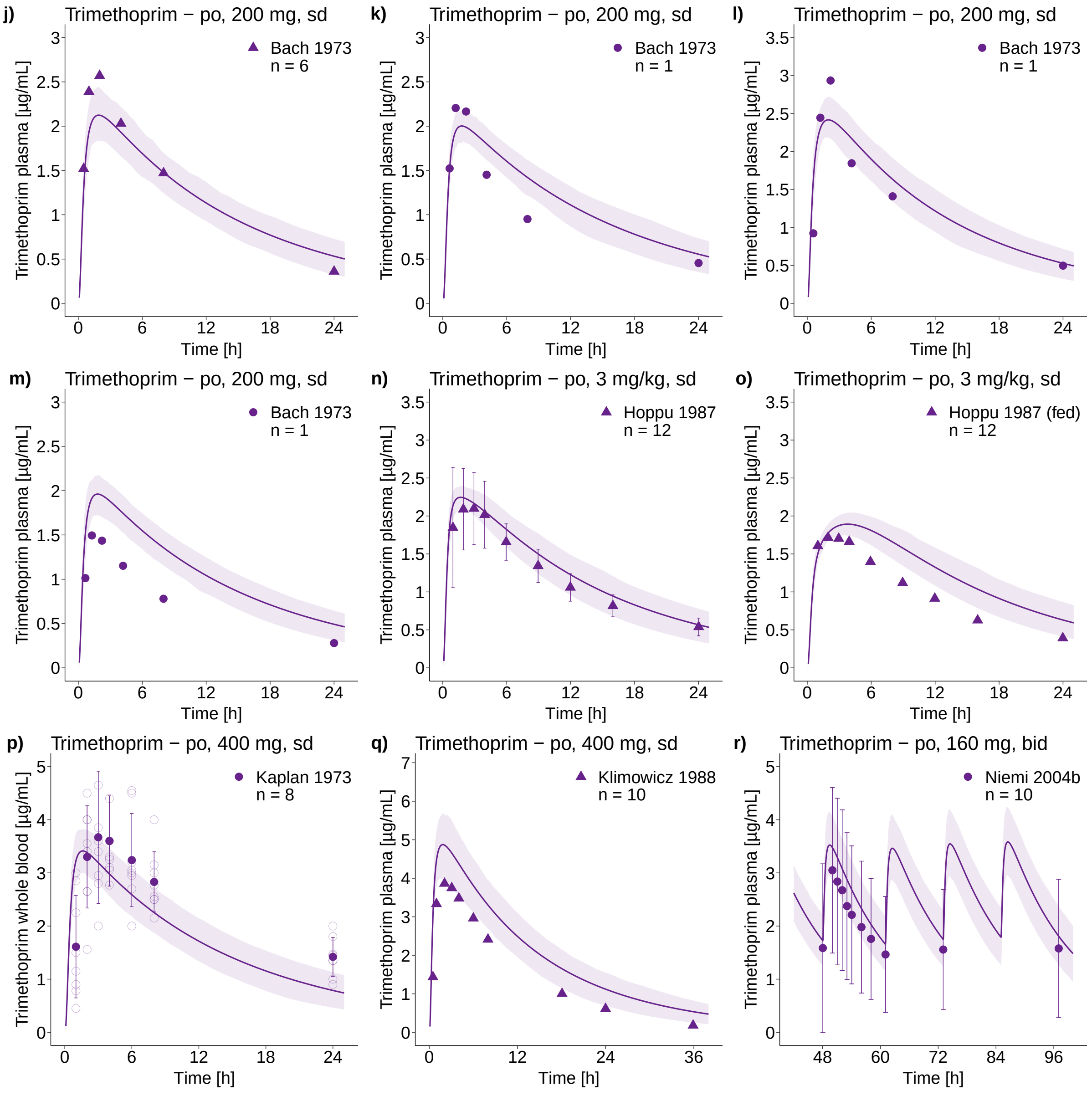

Figure S6: Trimethoprim plasma (or whole blood) concentration-time profiles (linear). Observed data are shown as triangles (training dataset) or circles (test dataset) ± standard deviation. Population simulation arithmetic means are shown as lines; the shaded areas represent the 68% population prediction intervals. Details on dosing regimens, study populations and literature references are listed in Table S1. Predicted and observed AUC_{last} and C_{max} values are summarized in Table S5. *bid* twice daily, *n* number of individuals studied, *po* oral, *sd* single dose. *(continued)*

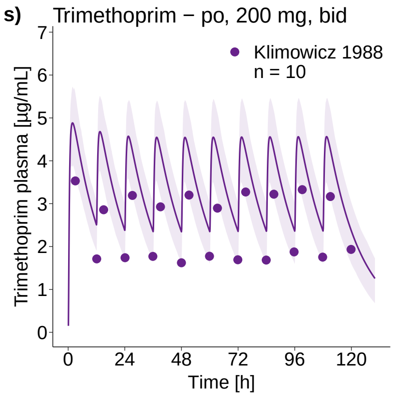

Figure S6: Trimethoprim plasma (or whole blood) concentration-time profiles (linear). Observed data are shown as triangles (training dataset) or circles (test dataset) ± standard deviation. Population simulation arithmetic means are shown as lines; the shaded areas represent the 68% population prediction intervals. Details on dosing regimens, study populations and literature references are listed in Table S1. Predicted and observed AUC_{last} and C_{max} values are summarized in Table S5. *bid* twice daily, *n* number of individuals studied, *po* oral. *(continued)*

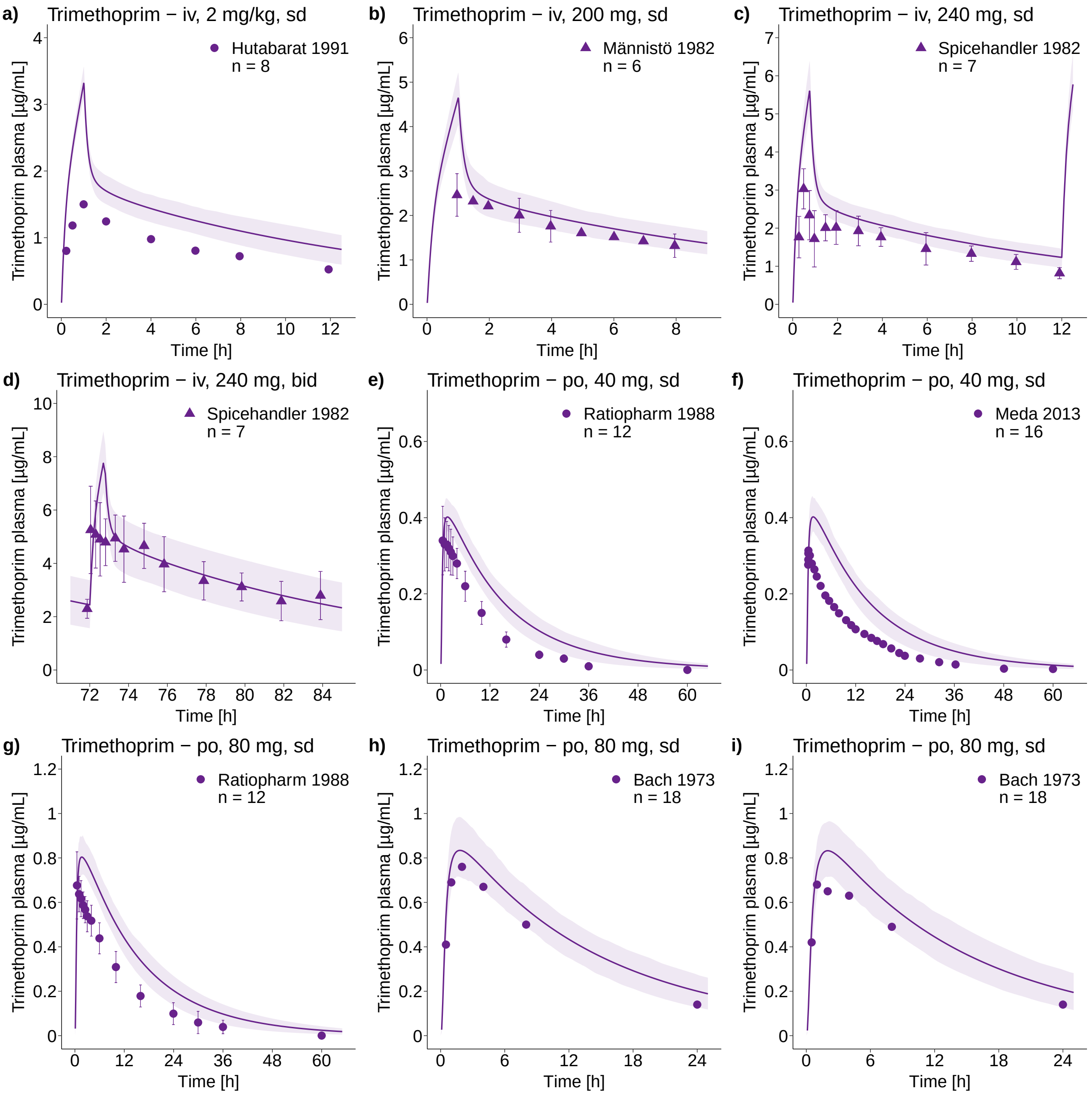

Figure S7: Trimethoprim plasma (or whole blood) concentration-time profiles after "cotrimoxazole" administration (linear). Observed data are shown as triangles (training dataset) or circles (test dataset) ± standard deviation. Population simulation arithmetic means are shown as lines; the shaded areas represent the 68% population prediction intervals. Details on dosing regimens, study populations and literature references are listed in Table S1. Predicted and observed AUC_{last} and C_{max} values are summarized in Table S5. *iv* intravenous, *n* number of individuals studied, *po* oral, *sd* single dose.

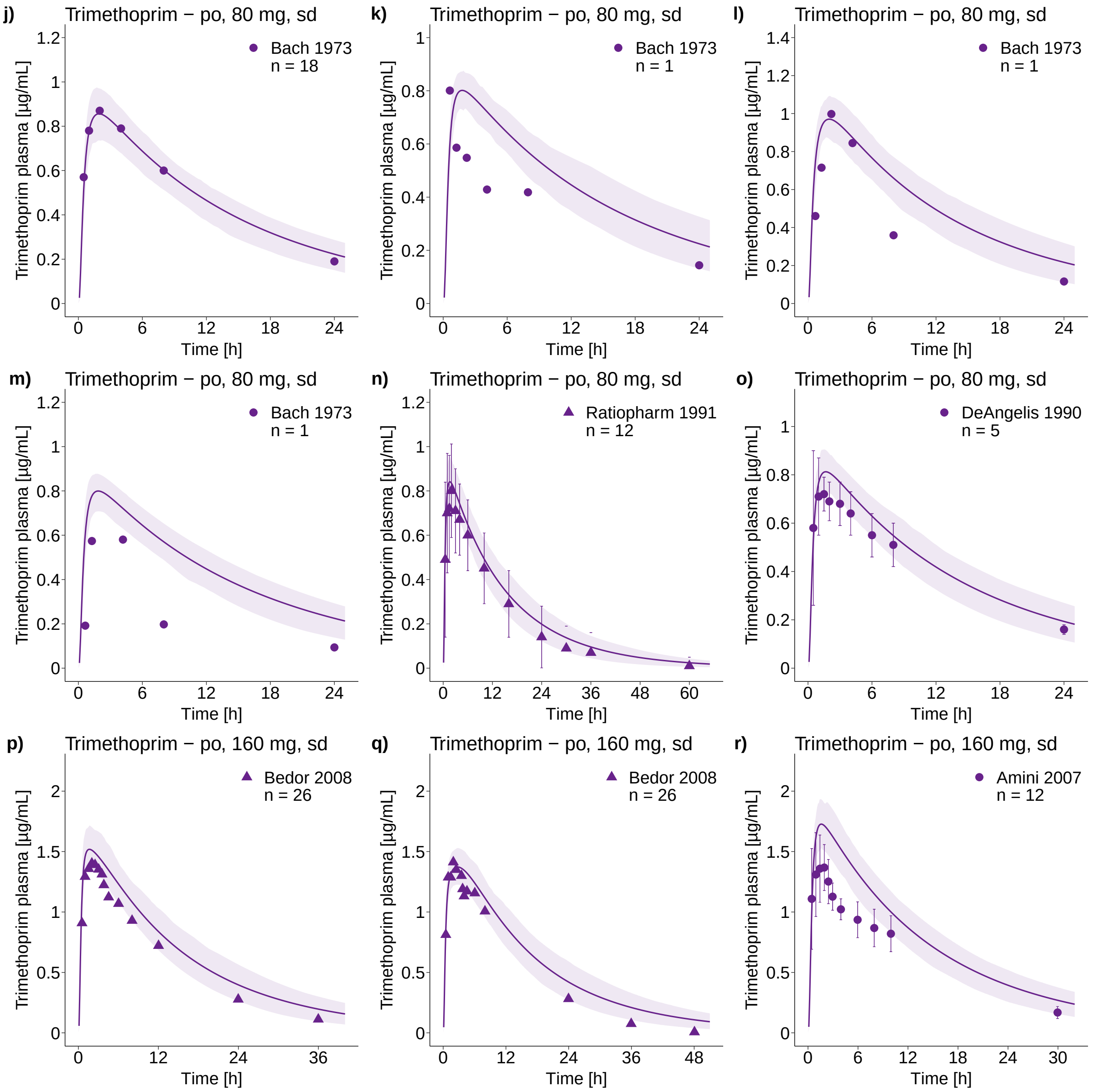

Figure S7: Trimethoprim plasma (or whole blood) concentration-time profiles after "cotrimoxazole" administration (linear). Observed data are shown as triangles (training dataset) or circles (test dataset) ± standard deviation. Population simulation arithmetic means are shown as lines; the shaded areas represent the 68% population prediction intervals. Details on dosing regimens, study populations and literature references are listed in Table S1. Predicted and observed AUC_{last} and C_{max} values are summarized in Table S5. *n* number of individuals studied, *po* oral, *sd* single dose. *(continued)*

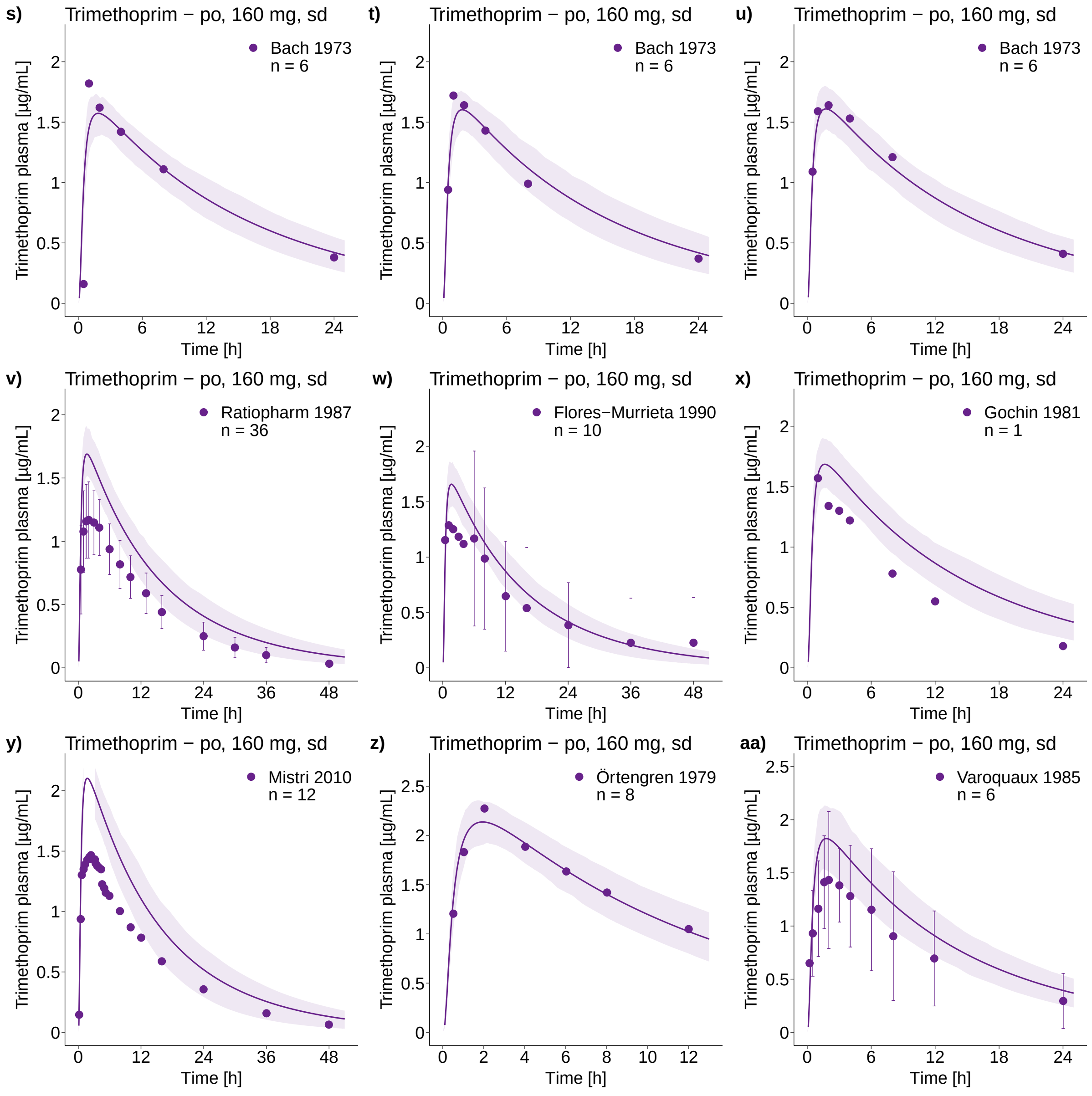

Figure S7: Trimethoprim plasma (or whole blood) concentration-time profiles after "cotrimoxazole" administration (linear). Observed data are shown as triangles (training dataset) or circles (test dataset) ± standard deviation. Population simulation arithmetic means are shown as lines; the shaded areas represent the 68% population prediction intervals. Details on dosing regimens, study populations and literature references are listed in Table S1. Predicted and observed AUC_{last} and C_{max} values are summarized in Table S5. *n* number of individuals studied, *po* oral, *sd* single dose. *(continued)*

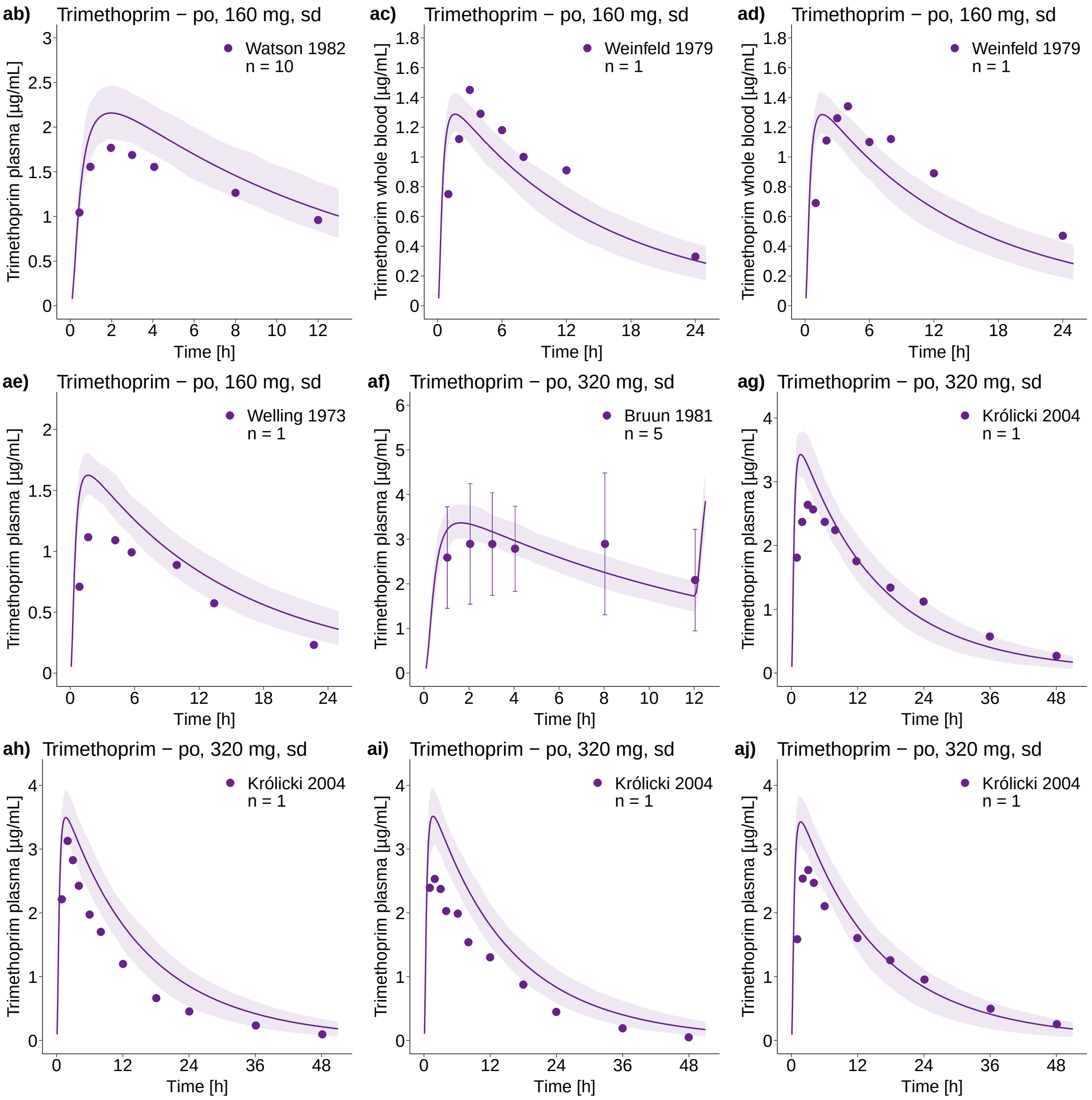

Figure S7: Trimethoprim plasma (or whole blood) concentration-time profiles after "cotrimoxazole" administration (linear). Observed data are shown as triangles (training dataset) or circles (test dataset) ± standard deviation. Population simulation arithmetic means are shown as lines; the shaded areas represent the 68% population prediction intervals. Details on dosing regimens, study populations and literature references are listed in Table S1. Predicted and observed AUC_{last} and C_{max} values are summarized in Table S5. *n* number of individuals studied, *po* oral, *sd* single dose. *(continued)*

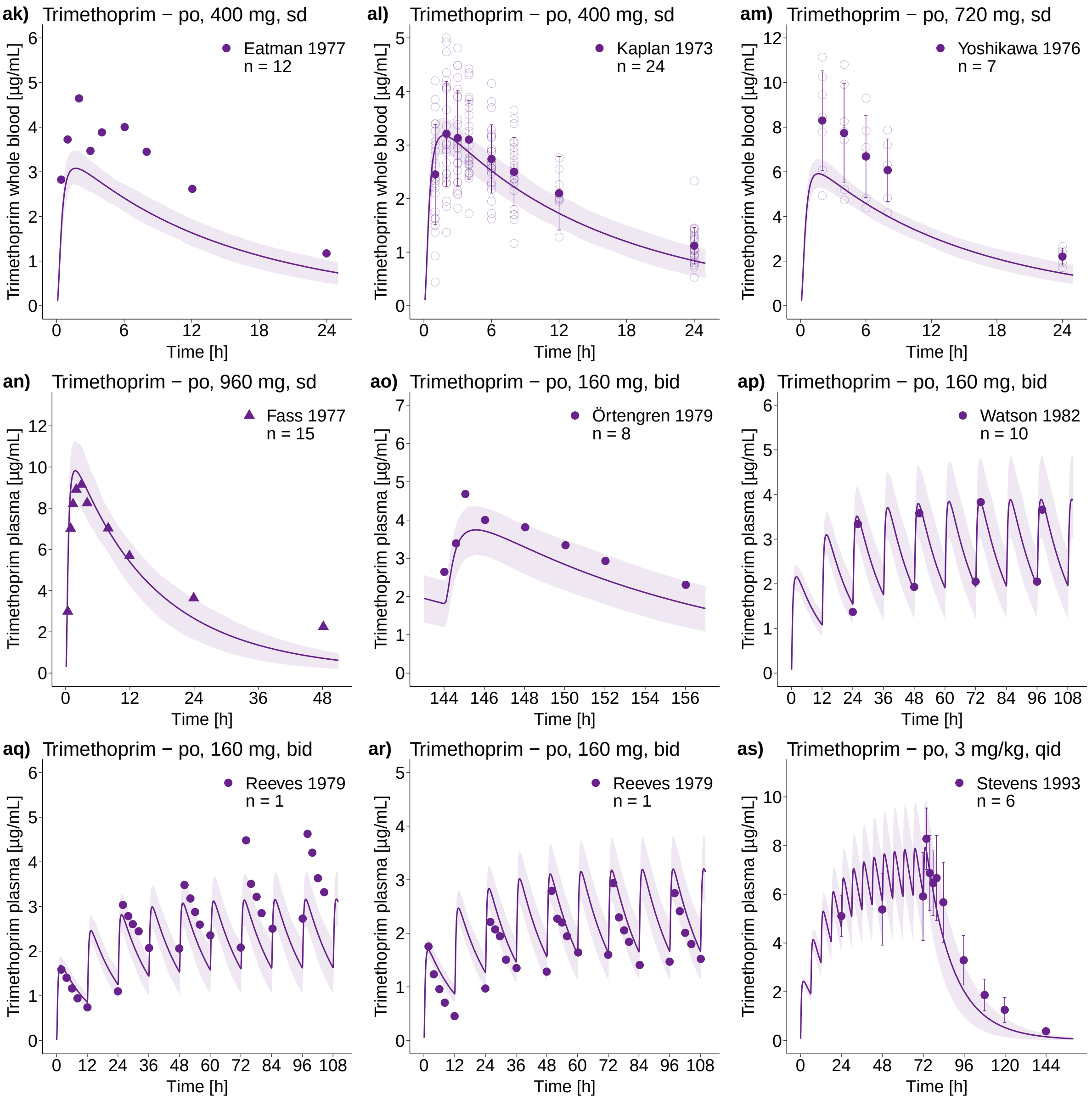

Figure S7: Trimethoprim plasma (or whole blood) concentration-time profiles after "cotrimoxazole" administration (linear). Observed data are shown as triangles (training dataset) or circles (test dataset) ± standard deviation. Population simulation arithmetic means are shown as lines; the shaded areas represent the 68% population prediction intervals. Details on dosing regimens, study populations and literature references are listed in Table S1. Predicted and observed AUC_{last} and C_{max} values are summarized in Table S5. *bid* twice daily, *n* number of individuals studied, *po* oral, *sd* single dose, *qid* four times daily. *(continued)*

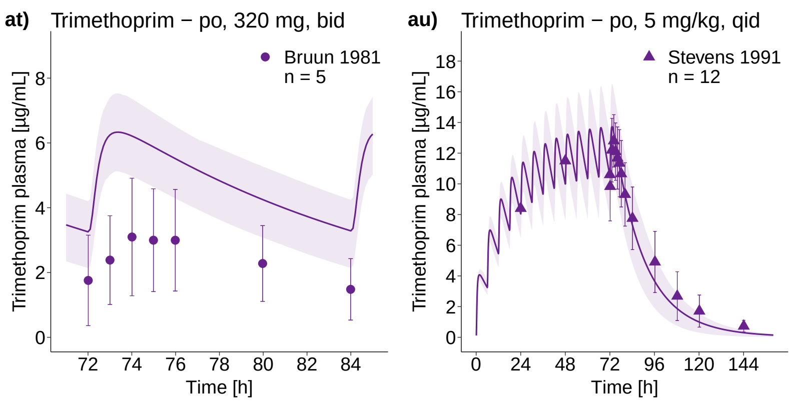

Figure S7: Trimethoprim plasma (or whole blood) concentration-time profiles after "cotrimoxazole" administration (linear). Observed data are shown as triangles (training dataset) or circles (test dataset) ± standard deviation. Population simulation arithmetic means are shown as lines; the shaded areas represent the 68% population prediction intervals. Details on dosing regimens, study populations and literature references are listed in Table S1. Predicted and observed AUC_{last} and C_{max} values are summarized in Table S5. *bid* twice daily, *n* number of individuals studied, *po* oral, *qid* four times daily. *(continued)*

2.4.3 Linear plots - Fraction excreted unchanged in urine

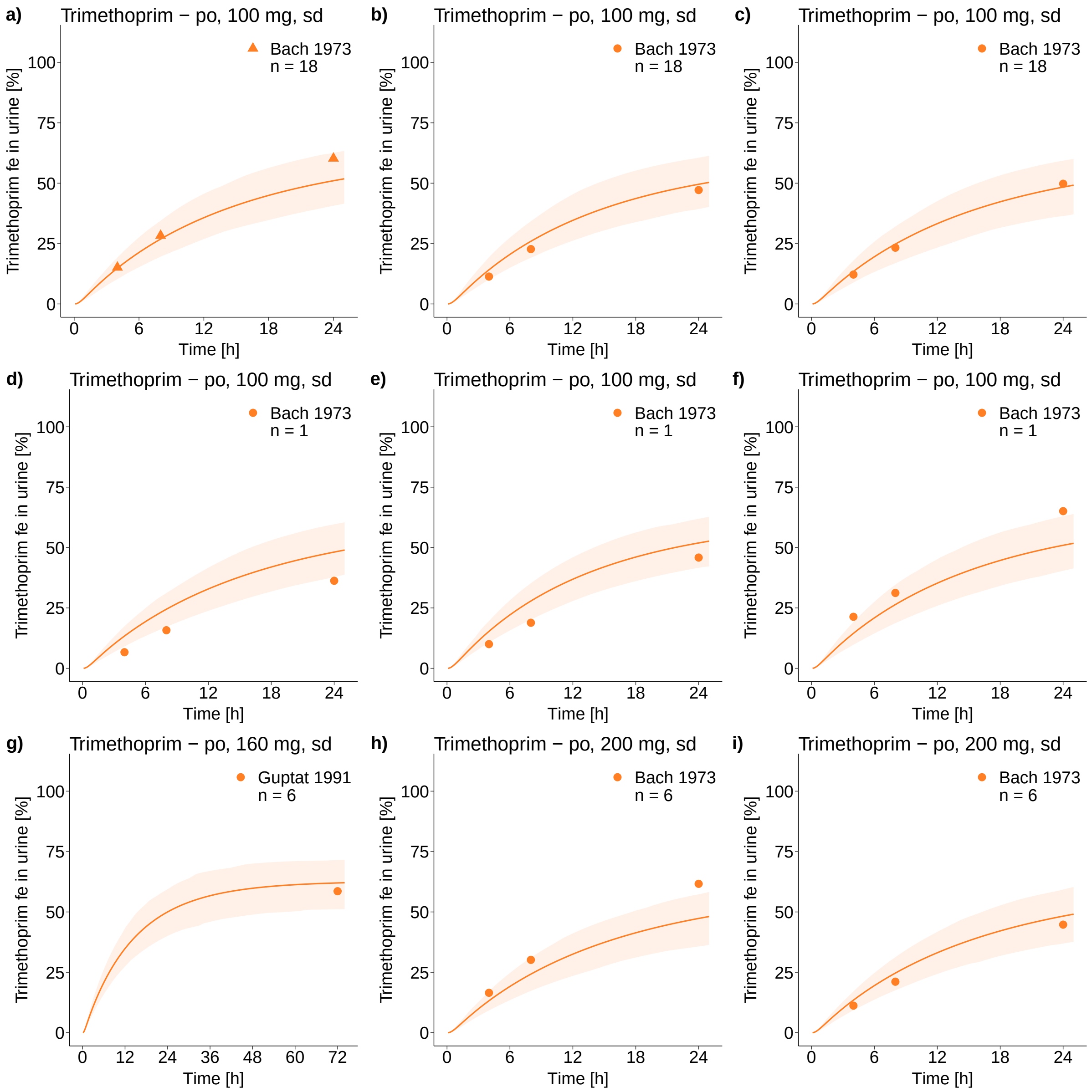

Figure S8: Trimethoprim fraction excreted unchanged in urine profiles. Observed data are shown as triangles (training dataset) or circles (test dataset) ± standard deviation. Population simulation arithmetic means are shown as lines; the shaded areas represent the 68% population prediction intervals. Details on dosing regimens, study populations and literature references are listed in Table S1. Predicted and observed fractions excreted unchanged in urine are summarized in Table S4. *fe in urine* fraction excreted unchanged in urine, *n* number of individuals studied, *po* oral, *sd* single dose.

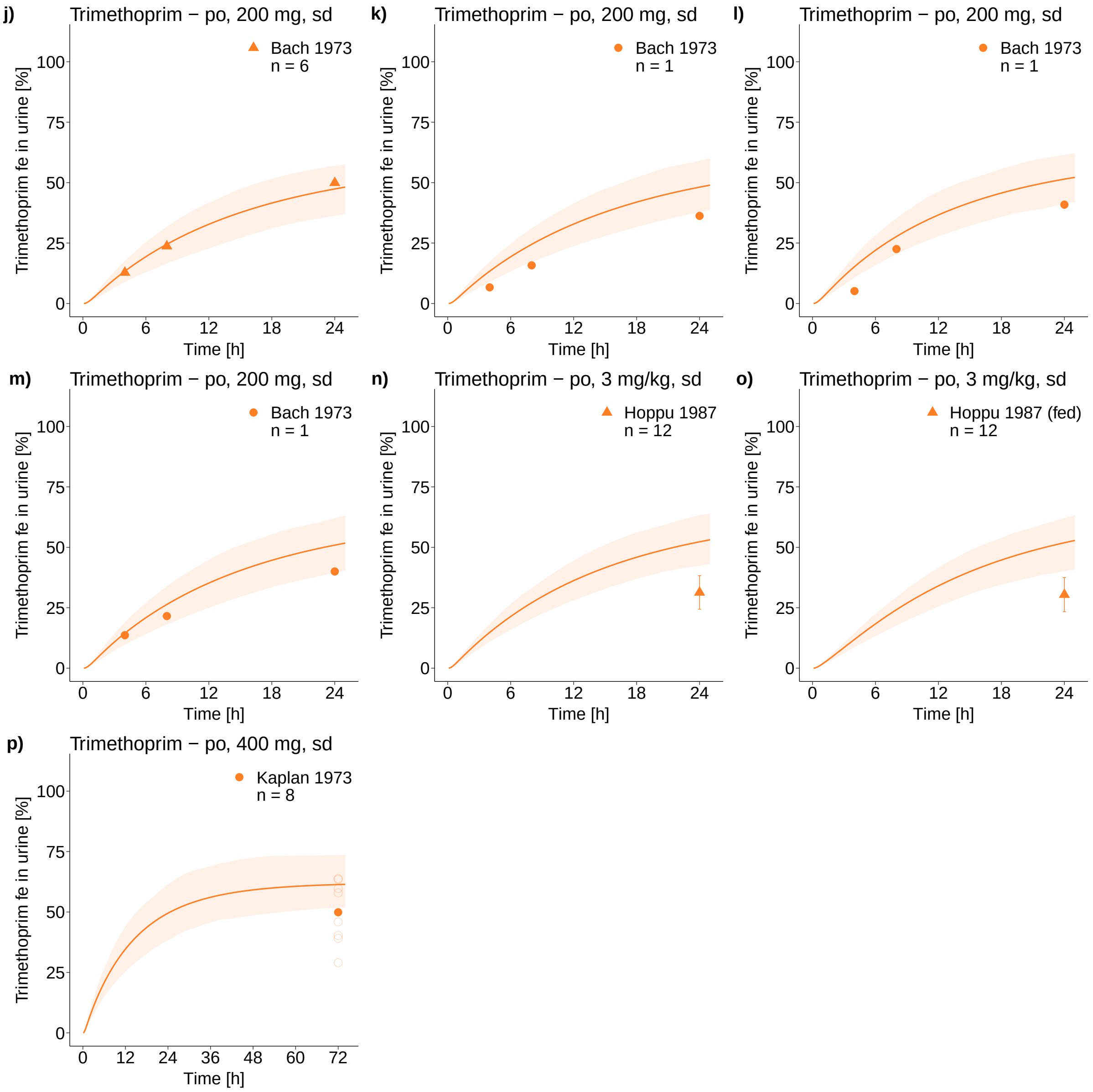

Figure S8: Trimethoprim fraction excreted unchanged in urine profiles. Observed data are shown as triangles (training dataset) or circles (test dataset) ± standard deviation. Population simulation arithmetic means are shown as lines; the shaded areas represent the 68% population prediction intervals. Details on dosing regimens, study populations and literature references are listed in Table S1. Predicted and observed fractions excreted unchanged in urine are summarized in Table S4. *fe in urine* fraction excreted unchanged in urine, *n* number of individuals studied, *po* oral, *sd* single dose. *(continued)*

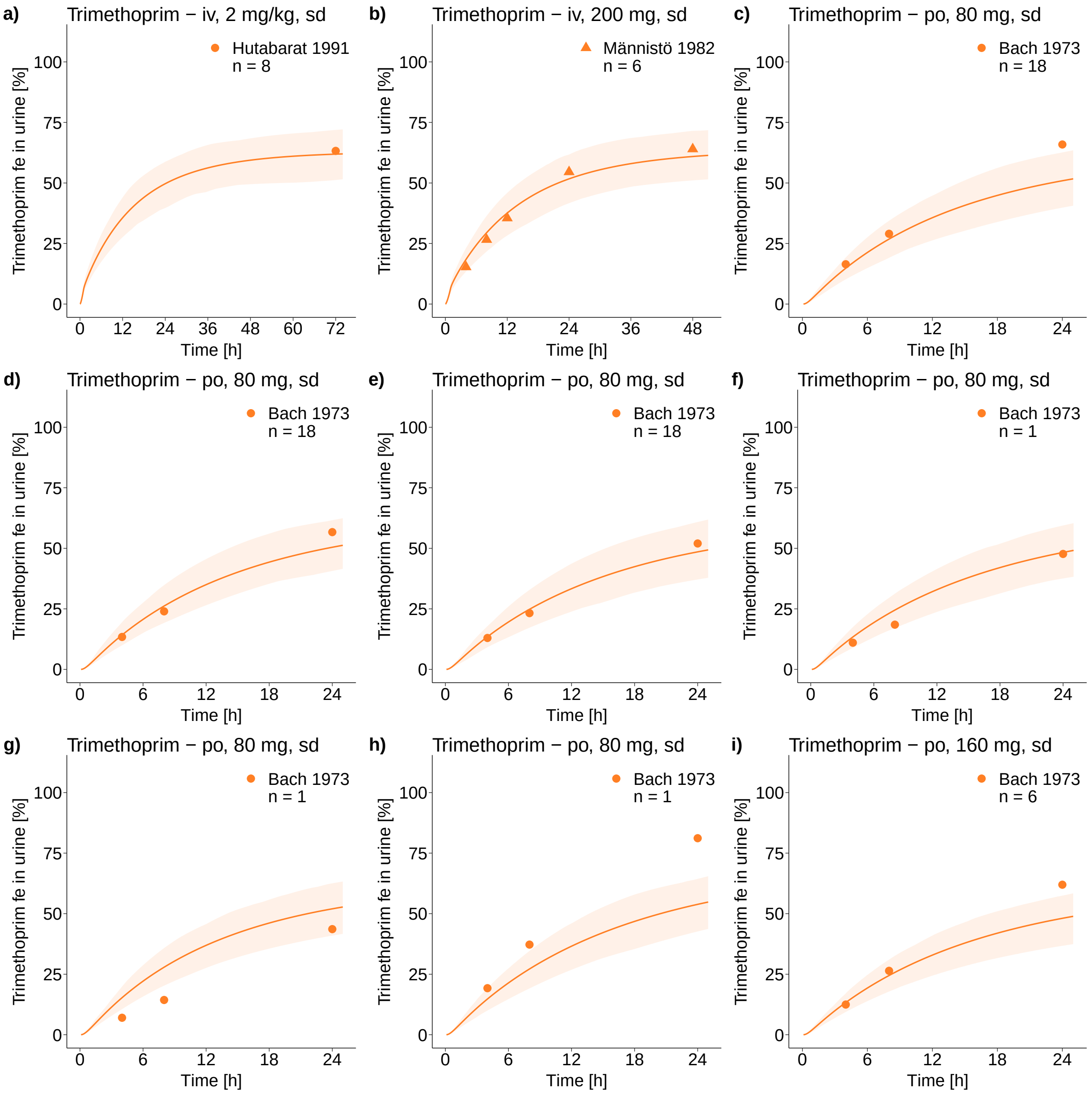

Figure S9: Trimethoprim fraction excreted unchanged in urine profiles after "cotrimoxazole" administration. Observed data are shown as triangles (training dataset) or circles (test dataset) ± standard deviation. Population simulation arithmetic means are shown as lines; the shaded areas represent the 68% population prediction intervals. Details on dosing regimens, study populations and literature references are listed in Table S1. Predicted and observed fractions excreted unchanged in urine are summarized in Table S4. *fe in urine* fraction excreted unchanged in urine, *iv* intravenous, *n* number of individuals studied, *po* oral, *sd* single dose.

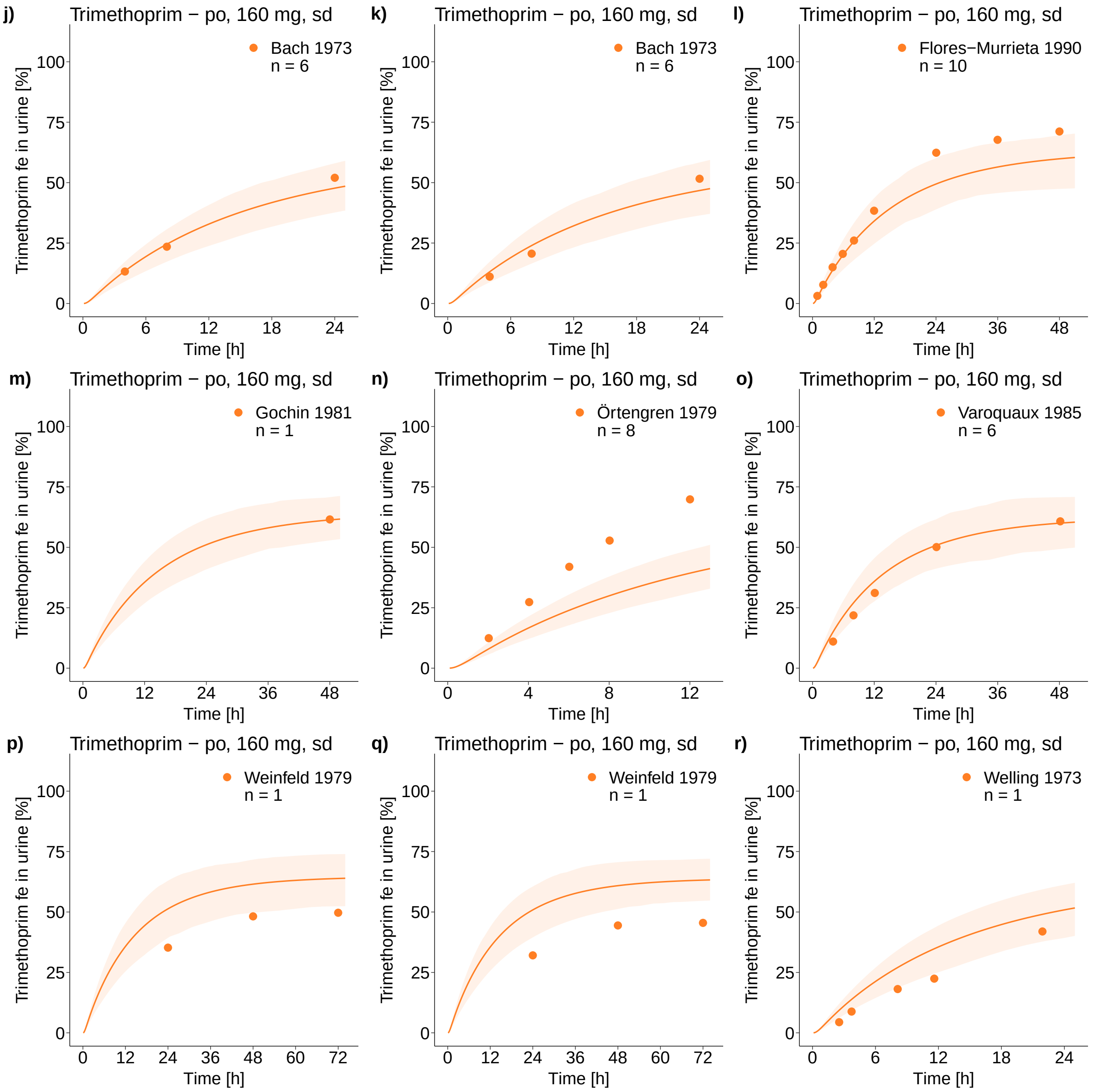

Figure S9: Trimethoprim fraction excreted unchanged in urine profiles after "cotrimoxazole" administration. Observed data are shown as triangles (training dataset) or circles (test dataset) ± standard deviation. Population simulation arithmetic means are shown as lines; the shaded areas represent the 68% population prediction intervals. Details on dosing regimens, study populations and literature references are listed in Table S1. Predicted and observed fractions excreted unchanged in urine are summarized in Table S4. *fe in urine* fraction excreted unchanged in urine, *n* number of individuals studied, *po* oral, *sd* single dose. *(continued)*

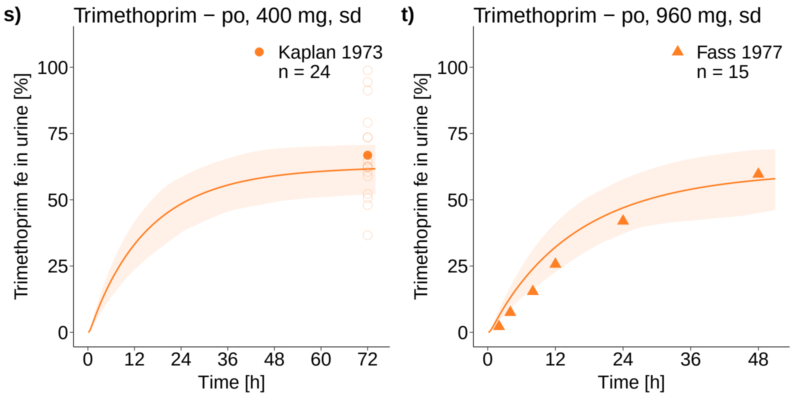

Figure S9: Trimethoprim fraction excreted unchanged in urine profiles after "cotrimoxazole" administration. Observed data are shown as triangles (training dataset) or circles (test dataset) ± standard deviation. Population simulation arithmetic means are shown as lines; the shaded areas represent the 68% population prediction intervals. Details on dosing regimens, study populations and literature references are listed in Table S1. Predicted and observed fractions excreted unchanged in urine are summarized in Table S4. *fe in urine* fraction excreted unchanged in urine, *n* number of individuals studied, *po* oral, *sd* single dose. *(continued)*

2.5 Trimethoprim PBPK model evaluation

2.5.1 Plasma and whole blood goodness-of-fit plot

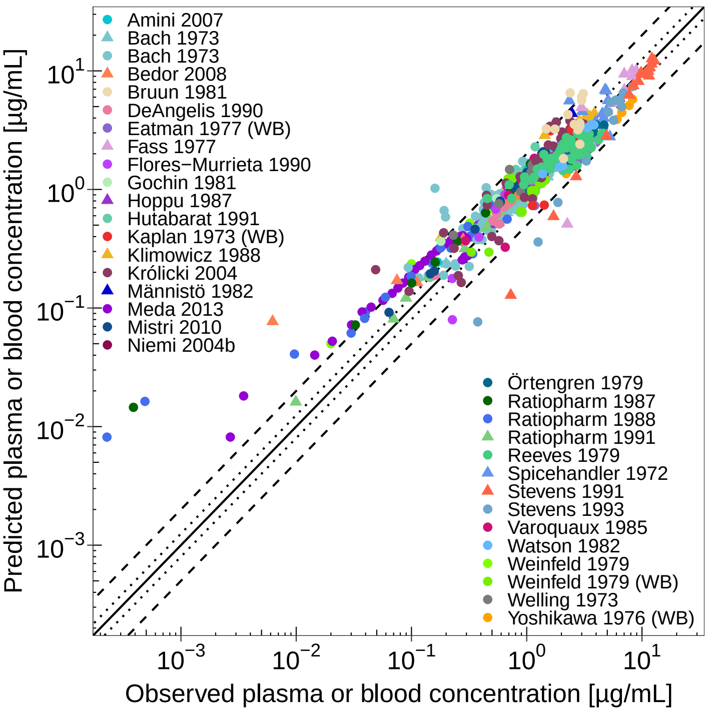

Figure S10: Comparison of predicted to the corresponding observed trimethoprim plasma (or whole blood) concentration values of all clinical studies. The solid line marks the line of identity, dotted lines indicate 1.25-fold and dashed lines indicate 2-fold deviation. Data are shown as triangles (training dataset) or dots (test dataset). Details on the study protocols are given in table S1. *WB* whole blood.

2.5.2 MRD of plasma and whole blood predictions

Table S3: MRD values of trimethoprim plasma (or whole blood) concentration predictions

Route	Compartment	Dose [mg]	MRD	Reference
Trimethoprim				
Oral				
po (tab, sd)	Venous Blood Plasma	100	1.10	Bach 1973 [29]
po (tab, sd)	Venous Blood Plasma	100	1.24	Bach 1973 [29]
po (tab, sd)	Venous Blood Plasma	100	1.23	Bach 1973 [29]
po (tab, sd)	Venous Blood Plasma	100	1.10	Bach 1973 [29]
po (tab, sd)	Venous Blood Plasma	100	1.37	Bach 1973 [29]
po (tab, sd)	Venous Blood Plasma	100	1.55	Bach 1973 [29]
po (-, sd)	Venous Blood Plasma	100	1.64	Weinfeld 1979 [25]
po (tab, sd)	Venous Blood Plasma	200	1.14	Bach 1973 [29]
po (tab, sd)	Venous Blood Plasma	200	1.19	Bach 1973 [29]
po (tab, sd)	Venous Blood Plasma	200	1.22	Bach 1973 [29]
po (tab, sd)	Venous Blood Plasma	200	1.25	Bach 1973 [29]
po (tab, sd)	Venous Blood Plasma	200	1.32	Bach 1973 [29]
po (tab, sd)	Venous Blood Plasma	200	1.58	Bach 1973 [29]
po (susp, sd)	Venous Blood Plasma	3 /kg	1.08	Hoppu 1987 [30]
po (susp, sd, fed)	Venous Blood Plasma	3 /kg	1.27	Hoppu 1987 [30]
po (tab, sd)	Venous Whole Blood	400	1.49	Kaplan 1973 [24]
po (-, sd)	Venous Blood Plasma	400	1.47	Klimowicz 1988 [31]
po (tab, bid)	Venous Blood Plasma	160	1.08	Niemi 2004b [32]
po (-, bid)	Venous Blood Plasma	400/200	1.15	Klimowicz 1988 [31]
**mean MRD (range)**			**1.29 (1.08 – 1.64)**<br>**19/19 with MRD ≤ 2**	
Cotrimoxazole				
Intravenous				
iv (1 h, sd)	Venous Blood Plasma	2 /kg	1.66	Hutabarat 1991 [33]
iv (1 h, sd)	Venous Blood Plasma	200	1.22	Mannistö 1982 [34]
iv (0.75 h, sd)	Venous Blood Plasma	240	1.54	Spicehandler 1982 [35]
iv (0.75 h, bid)	Venous Blood Plasma	240	1.27	Spicehandler 1982 [35]
**mean MRD (range)**			**1.42 (1.22 – 1.66)**<br>**4/4 with MRD ≤ 2**	
Oral				
po (susp, sd)	Venous Blood Plasma	40	3.04	Ratiopharm 1988 [36]
po (susp, sd)	Venous Blood Plasma	40	2.03	Meda 2013 [37]
po (susp, sd)	Venous Blood Plasma	80	2.82	Ratiopharm 1988 [36]
po (tab, sd)	Venous Blood Plasma	80	1.18	Bach 1973 [29]
po (tab, sd)	Venous Blood Plasma	80	1.21	Bach 1973 [29]
po (tab, sd)	Venous Blood Plasma	80	1.08	Bach 1973 [29]
po (tab, sd)	Venous Blood Plasma	80	1.49	Bach 1973 [29]
po (tab, sd)	Venous Blood Plasma	80	1.45	Bach 1973 [29]
po (tab, sd)	Venous Blood Plasma	80	2.35	Bach 1973 [29]
po (tab, sd)	Venous Blood Plasma	80	1.22	Ratiopharm 1991 [28]
po (-, sd)	Venous Blood Plasma	80	1.13	DeAngelis 1990 [38]

bid twice daily, *caps* capsule, *iv* intravenous, *MRD* mean relative deviation, *po* oral, *qid* four times daily, *sd* single dose, *susp* oral suspension, *tab* tablet.

Table S3: MRD values of trimethoprim plasma concentration predictions *(continued)*

Route	Compartment	Dose [mg]	MRD	Reference
po (susp, sd)	Venous Blood Plasma	160	1.19	Bedor 2008 [39]
po (caps, sd)	Venous Blood Plasma	160	2.04	Bedor 2008 [39]
po (tab, sd)	Venous Blood Plasma	160	1.31	Amini 2007 [40]
po (tab, sd)	Venous Blood Plasma	160	2.14	Bach 1973 [29]
po (tab, sd)	Venous Blood Plasma	160	1.07	Bach 1973 [29]
po (tab, sd)	Venous Blood Plasma	160	1.07	Bach 1973 [29]
po (tab, sd)	Venous Blood Plasma	160	2.68	Ratiopharm 1987 [28]
po (tab, sd)	Venous Blood Plasma	160	1.48	Flores-Murrieta 1990 [41]
po (tab, sd)	Venous Blood Plasma	160	1.44	Gochin 1981 [42]
po (tab, sd)	Venous Blood Plasma	160	1.38	Mistri 2010 [43]
po (tab, sd)	Venous Blood Plasma	160	1.07	Örtengren 1979 [44]
po (tab, sd)	Venous Blood Plasma	160	1.40	Varoquaux 1985 [45]
po (tab, sd)	Venous Blood Plasma	160	1.19	Watson 1982 [46]
po (-, sd)	Venous Whole Blood	160	1.28	Weinfeld 1979 [25]
po (-, sd)	Venous Whole Blood	160	1.37	Weinfeld 1979 [25]
po (-, sd)	Venous Blood Plasma	160	1.52	Welling 1973 [47]
po (tab, sd)	Venous Blood Plasma	320	1.22	Bruun 1981 [48]
po (-, sd)	Venous Blood Plasma	320	1.45	Królicki 2004 [49]
po (-, sd)	Venous Blood Plasma	320	1.41	Królicki 2004 [49]
po (-, sd)	Venous Blood Plasma	320	1.86	Królicki 2004 [49]
po (-, sd)	Venous Blood Plasma	320	1.38	Królicki 2004 [49]
po (tab, sd)	Venous Whole Blood	400	1.47	Eatman 1977 [50]
po (tab, sd)	Venous Whole Blood	400	1.23	Kaplan 1973 [24]
po (tab, sd)	Venous Whole Blood	720	1.53	Yoshikawa 1976 [51]
po (tab, sd)	Venous Blood Plasma	960	1.69	Fass 1977 [52]
po (tab, bid)	Venous Blood Plasma	160	1.35	Örtengren 1979 [44]
po (tab, bid)	Venous Blood Plasma	160	1.17	Watson 1982 [46]
po (-, bid)	Venous Blood Plasma	160	1.37	Reeves 1979 [53]
po (-, bid)	Venous Blood Plasma	160	1.21	Reeves 1979 [53]
po (-, qid)	Venous Blood Plasma	3 /kg	1.97	Stevens 1993 [54]
po (tab, bid)	Venous Blood Plasma	320	2.10	Bruun 1981 [48]
po (-, qid)	Venous Blood Plasma	5 /kg	1.77	Stevens 1991 [55]
**mean MRD (range)**			**1.55 (1.07 – 3.04)** **35/43 with MRD ≤ 2**	
**Overall MRD (range)**			**1.47 (1.07 – 3.04)** **58/66 with MRD ≤ 2**	

bid twice daily, *caps* capsule, *iv* intravenous, *MRD* mean relative deviation, *po* oral, *qid* four times daily, *sd* single dose, *susp* oral suspension, *tab* tablet.

2.5.3 Fraction excreted unchanged in urine goodness-of-fit plot

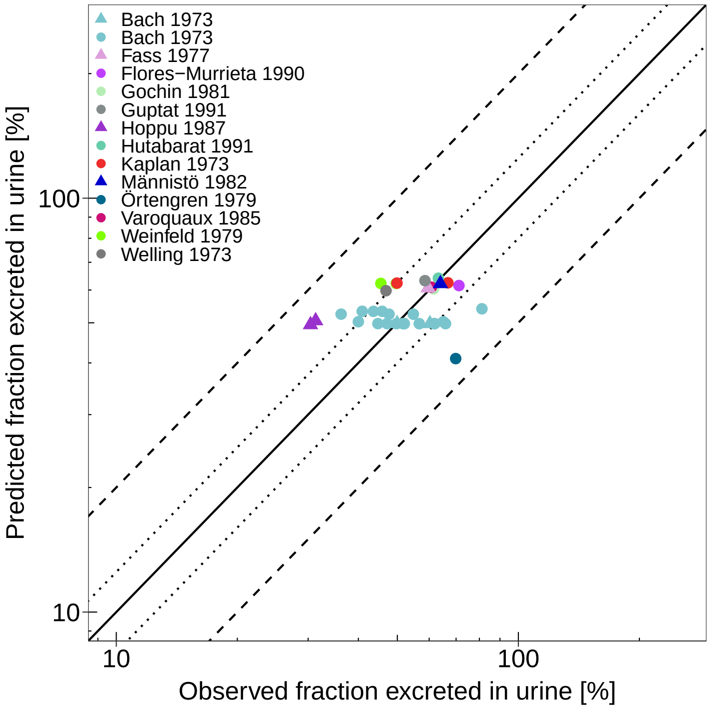

Figure S11: Comparison of predicted to observed trimethoprim fractions excreted unchanged in urine of all clinical studies. The solid line marks the line of identity, dotted lines indicate 1.25-fold and dashed lines indicate 2-fold deviation. Data are shown as triangles (training dataset) or dots (test dataset). Details on the study protocols and the predicted and observed fraction excreted unchanged in urine values are given in Tables S1 and S4, respectively.

2.5.4 Predicted and observed fractions excreted unchanged in urine with mean GMFE values and ranges

Table S4: Predicted and observed trimethoprim fractions excreted unchanged in urine

			fe in urine			
Route	Dose [mg]	t_{last} [h]	Pred [%]	Obs [%]	Pred/Obs	Reference
Trimethoprim						
Oral						
po (tab, sd)	100	24	49.8	60.3	0.83	Bach 1973 [29]
po (tab, sd)	100	24	49.8	47.2	1.06	Bach 1973 [29]
po (tab, sd)	100	24	49.8	49.8	1.00	Bach 1973 [29]
po (tab, sd)	100	24	52.4	54.8	0.96	Bach 1973 [29]
po (tab, sd)	100	24	53.3	45.9	1.16	Bach 1973 [29]
po (tab, sd)	100	24	50.3	65.1	0.77	Bach 1973 [29]
po (-, sd)	160	72 [a]	63.2	58.6	1.08	Guptat 1991 [26]
po (tab, sd)	200	24	49.8	61.6	0.81	Bach 1973 [29]
po (tab, sd)	200	24	49.8	44.7	1.11	Bach 1973 [29]
po (tab, sd)	200	24	49.8	49.9	1.00	Bach 1973 [29]
po (tab, sd)	200	24	52.5	36.2	1.45	Bach 1973 [29]
po (tab, sd)	200	24	53.3	40.9	1.30	Bach 1973 [29]
po (tab, sd)	200	24	50.3	40.0	1.26	Bach 1973 [29]
po (susp, sd)	3 /kg	24	50.6	31.3	1.61	Hoppu 1987 [30]
po (susp, sd, fed)	3 /kg	24	49.5	30.4	1.63	Hoppu 1987 [30]
po (tab, sd)	400	72	62.4	49.9	1.25	Kaplan 1973 [24]
**mean GMFE (range)**					**1.22 (1.00 – 1.63)** **16/16 with GMFE ≤ 2**	
Cotrimoxazole						
Intravenous						
iv (1 h, sd)	2 /kg	72	64.1	63.3	1.01	Hutabarat 1991 [33]
iv (1 h, sd)	200	48	62.2	64.0	0.97	Mannistö 1982 [34]
**mean GMFE (range)**					**1.02 (1.01 – 1.03)** **2/2 with GMFE ≤ 2**	
Oral						
po (tab, sd)	80	24	49.8	65.9	0.76	Bach 1973 [29]
po (tab, sd)	80	24	49.8	56.7	0.88	Bach 1973 [29]
po (tab, sd)	80	24	49.8	52.0	0.96	Bach 1973 [29]
po (tab, sd)	80	24	52.4	47.7	1.10	Bach 1973 [29]
po (tab, sd)	80	24	53.3	43.6	1.22	Bach 1973 [29]
po (tab, sd)	80	24	54.0	81.2	0.67	Bach 1973 [29]
po (tab, sd)	160	24	49.8	62.0	0.80	Bach 1973 [29]
po (tab, sd)	160	24	49.8	52.0	0.96	Bach 1973 [29]
po (tab, sd)	160	24	49.8	51.6	0.96	Bach 1973 [29]
po (tab, sd)	160	48	61.5	71.2	0.86	Flores-Murrieta 1990 [41]
po (tab, sd)	160	48	60.4	61.5	0.98	Gochin 1981 [42]
po (tab, sd)	160	12	41.0	69.8	0.59	Örtengren 1979 [44]
po (tab, sd)	160	48	61.0	60.8	1.00	Varoquaux 1985 [45]
po (-, sd)	160	72	62.2	49.7	1.25	Weinfeld 1979 [25]
po (-, sd)	160	72	62.2	45.5	1.37	Weinfeld 1979 [25]
po (-, sd)	160	48	59.8	46.9	1.28	Welling 1973 [47]
po (tab, sd)	400	72	62.5	66.8	0.93	Kaplan 1973 [24]
po (tab, sd)	960	48	60.7	59.6	1.02	Fass 1977 [52]
**mean GMFE (range)**					**1.18 (1.00 – 1.70)** **18/18 with GMFE ≤ 2**	
**Overall GMFE (range)**					**1.19 (1.00 – 1.70)** **36/36 with GMFE ≤ 2**	

[a] time assumed. *fe in urine* fraction excreted unchanged in urine, *GMFE* geometric mean fold error, *iv* intravenous, *obs* observed, *po* oral, *pred* predicted, *sd* single dose, *susp* oral suspension, *tab* tablet, t_{last} time of the last urine measurement.

2.5.5 AUC_{last} and C_{max} goodness-of-fit plots

a) AUC_{last}

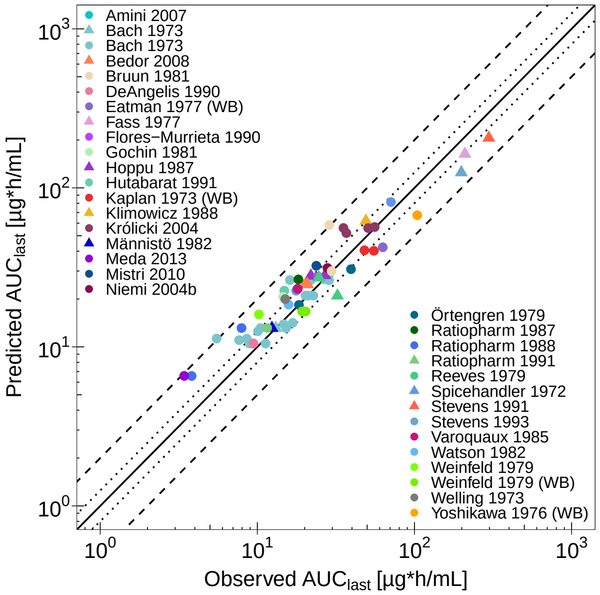

b) C_{max}

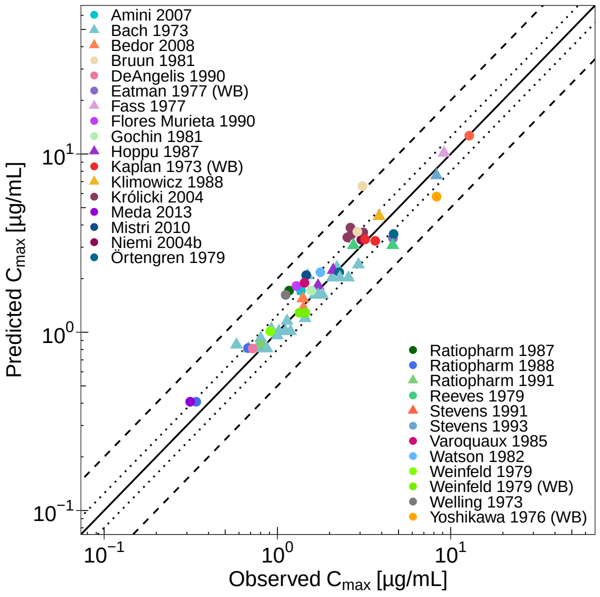

Figure S12: Comparison of predicted to the corresponding observed trimethoprim (**a**) AUC_{last} and (**b**) C_{max} values of all clinical studies. The solid line marks the line of identity, dotted lines indicate 1.25-fold and dashed lines indicate 2-fold deviation. Data are shown as triangles (training dataset) or dots (test dataset). Details on the study protocols and the predicted and observed AUC_{last} and C_{max} values are given in Tables S1 and S5, respectively. *WB* whole blood.

2.5.6 Predicted and observed AUC_{last} and C_{max} values with mean GMFE values and ranges

Table S5: Predicted and observed trimethoprim AUC_{last} and C_{max} values

				AUC_{last}			C_{max}			
Route	Compartment	Dose [mg]	t_{last} [h]	Pred [µg*h/mL]	Obs [µg*h/mL]	Pred/Obs	Pred [µg/mL]	Obs [µg/mL]	Pred/Obs	Reference
Trimethoprim										
Oral										
po (tab, sd)	Venous Blood Plasma	100	24	13.1	13.1	1.00	1.01	1.11	0.91	Bach 1973 [29]
po (tab, sd)	Venous Blood Plasma	100	24	13.1	10.5	1.25	1.01	1.02	0.99	Bach 1973 [29]
po (tab, sd)	Venous Blood Plasma	100	24	13.1	15.2	0.86	1.01	1.19	0.85	Bach 1973 [29]
po (tab, sd)	Venous Blood Plasma	100	24	13.9	14.7	0.94	1.16	1.14	1.02	Bach 1973 [29]
po (tab, sd)	Venous Blood Plasma	100	24	14.2	16.8	0.84	1.20	1.44	0.83	Bach 1973 [29]
po (tab, sd)	Venous Blood Plasma	100	24	12.6	10.1	1.24	1.04	0.92	1.13	Bach 1973 [29]
po (-, sd)	Venous Blood Plasma	100	48	16.0	10.2	1.56	1.01	0.91	1.11	Weinfeld 1979 [25]
po (tab, sd)	Venous Blood Plasma	200	24	26.3	28.6	0.92	2.03	2.29	0.88	Bach 1973 [29]
po (tab, sd)	Venous Blood Plasma	200	24	26.3	21.8	1.20	2.03	2.07	0.98	Bach 1973 [29]
po (tab, sd)	Venous Blood Plasma	200	24	26.3	27.6	0.95	2.03	2.57	0.79	Bach 1973 [29]
po (tab, sd)	Venous Blood Plasma	200	24	27.6	22.0	1.26	2.31	2.20	1.05	Bach 1973 [29]
po (tab, sd)	Venous Blood Plasma	200	24	28.4	28.5	1.00	2.40	2.93	0.82	Bach 1973 [29]
po (tab, sd)	Venous Blood Plasma	200	24	26.3	16.1	1.63	2.08	1.49	1.39	Bach 1973 [29]
po (susp, sd)	Venous Blood Plasma	3 /kg	24	28.2	27.0	1.05	2.23	2.10	1.06	Hoppu 1987 [30]
po (susp, sd, fed)	Venous Blood Plasma	3 /kg	24	28.0	21.9	1.28	1.82	1.72	1.06	Hoppu 1987 [30]
po (tab, sd)	Venous Whole Blood	400	24	40.2	55.2	0.73	3.26	3.67	0.89	Kaplan 1973 [24]
po (-, sd)	Venous Blood Plasma	400	36	62.0	49.1	1.26	4.48	3.86	1.16	Klimowicz 1988 [31]
po (tab, bid)	Venous Blood Plasma	160	24	31.3	27.9	1.12	3.30	3.05	1.08	Niemi 2004b [32]
po (-, bid) [a]	Venous Blood Plasma	200	-	-	-	-	-	-	-	Klimowicz 1988 [31]
**mean GMFE (range)**						**1.21 (1.00 – 1.63)** **18/18 with GMFE ≤ 2**			**1.13 (1.01 – 1.39)** **18/18 with GMFE ≤ 2**	
Cotrimoxazole										
Intravenous										
iv (1 h, sd)	Venous Blood Plasma	2 /kg	24	22.6	14.8	1.52	-	-	-	Hutabarat 1991 [33]
iv (1 h, sd)	Venous Blood Plasma	200	8	13.1	12.4	1.06	-	-	-	Mannistö 1982 [34]
iv (0.75 h, sd)	Venous Blood Plasma	240	12	22.6	17.7	1.28	-	-	-	Spicehandler 1982 [35]
iv (0.75 h, bid)	Venous Blood Plasma	240	12	81.4	70.8	1.15	-	-	-	Spicehandler 1982 [35]
**mean GMFE (range)**						**1.25 (1.06 – 1.52)** **4/4 with GMFE ≤ 2**				
Oral										
po (susp, sd)	Venous Blood Plasma	40	60	6.6	3.8	1.72	0.41	0.34	1.20	Ratiopharm 1988 [36]
po (susp, sd)	Venous Blood Plasma	40	60	6.6	3.4	1.92	0.41	0.31	1.30	Meda 2013 [37]
po (susp, sd)	Venous Blood Plasma	80	60	13.1	7.9	1.66	0.82	0.68	1.20	Ratiopharm 1988 [36]
po (tab, sd)	Venous Blood Plasma	80	24	10.5	9.3	1.13	0.81	0.76	1.07	Bach 1973 [29]
po (tab, sd)	Venous Blood Plasma	80	24	10.5	8.9	1.18	0.81	0.68	1.19	Bach 1973 [29]
po (tab, sd)	Venous Blood Plasma	80	24	10.5	11.3	0.93	0.81	0.87	0.93	Bach 1973 [29]
po (tab, sd)	Venous Blood Plasma	80	24	11.0	7.6	1.44	0.92	0.80	1.15	Bach 1973 [29]
po (tab, sd)	Venous Blood Plasma	80	24	11.3	8.6	1.31	0.96	1.00	0.96	Bach 1973 [29]
po (tab, sd)	Venous Blood Plasma	80	24	11.3	5.5	2.05	0.85	0.58	1.47	Bach 1973 [29]
po (tab, sd)	Venous Blood Plasma	80	60	13.1	11.6	1.14	0.86	0.80	1.07	Ratiopharm 1991 [28]
po (-, sd)	Venous Blood Plasma	80	24	10.5	9.5	1.11	0.81	0.72	1.13	DeAngelis 1990 [38]

[a] No calculation of AUC_{last} or C_{max}, as only peak and trough values after multiple dose administration are given. *AUC* area under the concentration-time curve, *bid* twice daily, *caps* capsule, C_{max} peak plasma concentration, *GMFE* geometric mean fold error, *iv* intravenous, *obs* observed, *po* oral, *pred* predicted, *sd* single dose, *susp* oral suspension, *tab* tablet, t_{last} time of the last concentration measurement, *qid* four times daily.

Table S5: Predicted and observed trimethoprim AUC_{last} and C_{max} values *(continued)*

				AUC_{last}			C_{max}			
Route	Compartment	Dose [mg]	t_{last} [h]	Pred [µg*h/mL]	Obs [µg*h/mL]	Pred/Obs	Pred [µg/mL]	Obs [µg/mL]	Pred/Obs	Reference
po (susp, sd)	Venous Blood Plasma	160	48	24.7	20.9	1.19	1.55	1.40	1.10	Bedor 2008 [39]
po (caps, sd)	Venous Blood Plasma	160	48	24.8	20.2	1.22	1.37	1.41	0.97	Bedor 2008 [39]
po (tab, sd)	Venous Blood Plasma	160	30	23.2	18.0	1.29	1.71	1.37	1.25	Amini 2007 [40]
po (tab, sd)	Venous Blood Plasma	160	24	21.0	21.2	0.99	1.62	1.82	0.89	Bach 1973 [29]
po (tab, sd)	Venous Blood Plasma	160	24	21.0	20.3	1.04	1.62	1.72	0.94	Bach 1973 [29]
po (tab, sd)	Venous Blood Plasma	160	24	21.0	22.7	0.92	1.62	1.64	0.99	Bach 1973 [29]
po (tab, sd)	Venous Blood Plasma	160	72	26.6	18.3	1.46	1.71	1.17	1.47	Ratiopharm 1987 [28]
po (tab, sd)	Venous Blood Plasma	160	48	27.7	24.3	1.14	1.82	1.29	1.41	Flores-Murrieta 1990 [41]
po (tab, sd)	Venous Blood Plasma	160	24	20.7	14.6	1.42	1.71	1.57	1.09	Gochin 1981 [42]
po (tab, sd)	Venous Blood Plasma	160	48	32.4	23.7	1.36	2.09	1.47	1.43	Mistri 2010 [43]
po (tab, sd)	Venous Blood Plasma	160	12	18.4	18.4	1.00	2.17	2.27	0.95	Örtengren 1979 [44]
po (tab, sd)	Venous Blood Plasma	160	24	23.2	18.1	1.28	1.89	1.43	1.32	Varoquaux 1985 [45]
po (tab, sd)	Venous Blood Plasma	160	12	18.5	15.8	1.17	2.17	1.77	1.23	Watson 1982 [46]
po (-, sd)	Venous Whole Blood	160	24	16.7	19.3	0.87	1.29	1.45	0.89	Weinfeld 1979 [25]
po (-, sd)	Venous Whole Blood	160	24	16.7	20.3	0.82	1.29	1.34	0.96	Weinfeld 1979 [25]
po (-, sd)	Venous Blood Plasma	160	24	20.0	15.1	1.33	1.62	1.12	1.45	Welling 1973 [47]
po (tab, sd)	Venous Blood Plasma	320	12	30.0	29.7	1.01	3.67	2.89	1.27	Bruun 1981 [48]
po (-, sd)	Venous Blood Plasma	320	48	56.7	55.8	1.02	3.86	2.64	1.46	Królicki 2004 [49]
po (-, sd)	Venous Blood Plasma	320	48	52.0	36.9	1.41	3.63	3.13	1.16	Królicki 2004 [49]
po (-, sd)	Venous Blood Plasma	320	48	55.9	35.3	1.58	3.41	2.53	1.35	Królicki 2004 [49]
po (-, sd)	Venous Blood Plasma	320	48	55.8	50.7	1.10	3.53	2.67	1.32	Królicki 2004 [49]
po (tab, sd)	Venous Whole Blood	400	24	42.3	62.8	0.67	3.37	4.64	0.73	Eatman 1977 [50]
po (tab, sd)	Venous Whole Blood	400	24	40.5	48.1	0.84	3.32	3.21	1.03	Kaplan 1973 [24]
po (tab, sd)	Venous Whole Blood	720	24	67.3	104.2	0.65	5.77	8.30	0.70	Yoshikawa 1976 [51]
po (tab, sd)	Venous Blood Plasma	960	48	163.3	209.5	0.78	10.13	9.15	1.11	Fass 1977 [52]
po (tab, bid)	Venous Blood Plasma	160	12	30.8	39.4	0.78	3.56	4.68	0.76	Örtengren 1979 [44]
po (tab, bid) [a]	Venous Blood Plasma	160	-	-	-	-	-	-	-	Watson 1982 [46]
po (-, bid)	Venous Blood Plasma	160	12	21.0	32.3	0.65	3.08	4.63	0.66	Reeves 1979 [53]
po (-, bid)	Venous Blood Plasma	160	12	27.3	24.6	1.11	3.08	2.75	1.12	Reeves 1979 [53]
po (-, qid)	Venous Blood Plasma	3 /kg	72	124.9	198.6	0.63	7.58	8.28	0.92	Stevens 1993 [54]
po (tab, bid)	Venous Blood Plasma	320	12	58.4	28.7	2.04	6.60	3.10	2.13	Bruun 1981 [48]
po (-, qid)	Venous Blood Plasma	5 /kg	72	206.2	297.3	0.69	12.68	12.80	0.99	Stevens 1991 [55]
**mean GMFE (range)**						**1.33 (1.00 – 2.05)**<br>**40/42 with GMFE ≤ 2**			**1.23 (1.01 – 2.13)**<br>**41/42 with GMFE ≤ 2**	
**Overall GMFE (range)**						**1.29 (1.00 – 2.05)**<br>**62/64 with GMFE ≤ 2**			**1.20 (1.01 – 2.13)**<br>**59/60 with GMFE ≤ 2**	

[a] No calculation of AUC_{last} or C_{max}, as only peak and trough values after multiple dose administration are given. *AUC* area under the concentration-time curve, *bid* twice daily, *caps* capsule, C_{max} peak plasma concentration, *GMFE* geometric mean fold error, *iv* intravenous, *obs* observed, *po* oral, *pred* predicted, *sd* single dose, *susp* oral suspension, *tab* tablet, t_{last} time of the last concentration measurement, *qid* four times daily.

2.5.7 Sensitivity analysis

Sensitivity of the final model to single parameters (local sensitivity analysis) was calculated, measured as the relative change of the $AUC_{0–12}$, C_{max} or t_{max} at steady state of an oral 160 mg twice daily trimethoprim regimen. Parameters were included into the analysis if they have been optimized, if they are associated with optimized parameters, or if they could have a strong impact due to their use in the calculation of permeabilities or partition coefficients. Sensitivity analysis was carried out using a relative perturbation of 1000% (variation range 10.0, maximum number of 9 steps). The parameters evaluated during sensitivity analysis provided in Table S6. The trimethoprim model predictions are sensitive to the value of fraction unbound in plasma, for which a literature value is used in the model (56% [27]) (see Figure S13).

Table S6: Parameters evaluated during trimethoprim sensitivity analysis

Parameter	Value	Unit	Source
CL_{hep}	1.61E-02	1/min	Optimized
CYP3A4 k_{cat}	0.56	1/min	Optimized
CYP3A4 K_M	375.57	µmol/L	Optimized
Dissolution shape	1.00		Optimized
Dissolution time (50% dissolved)	52.59	minutes	Optimized
Fraction unbound	56	%	Literature
Intestinal permeability	1.24E-02	cm/min	Optimized
Lipophilicity	1.01		Optimized
P-gp k_{cat}	1.44	1/min	Optimized
P-gp K_M	195.75	µmol/L	Optimized
Solubility (pH 7.0)	0.40	g/L	Literature

CL_{hep} hepatic metabolic clearance, *CYP* cytochrome P450, k_{cat} transport or catalytic rate constant, K_M Michaelis-Menten constant, *P-gp* P-glycoprotein.

a) $AUC_{0–12}$

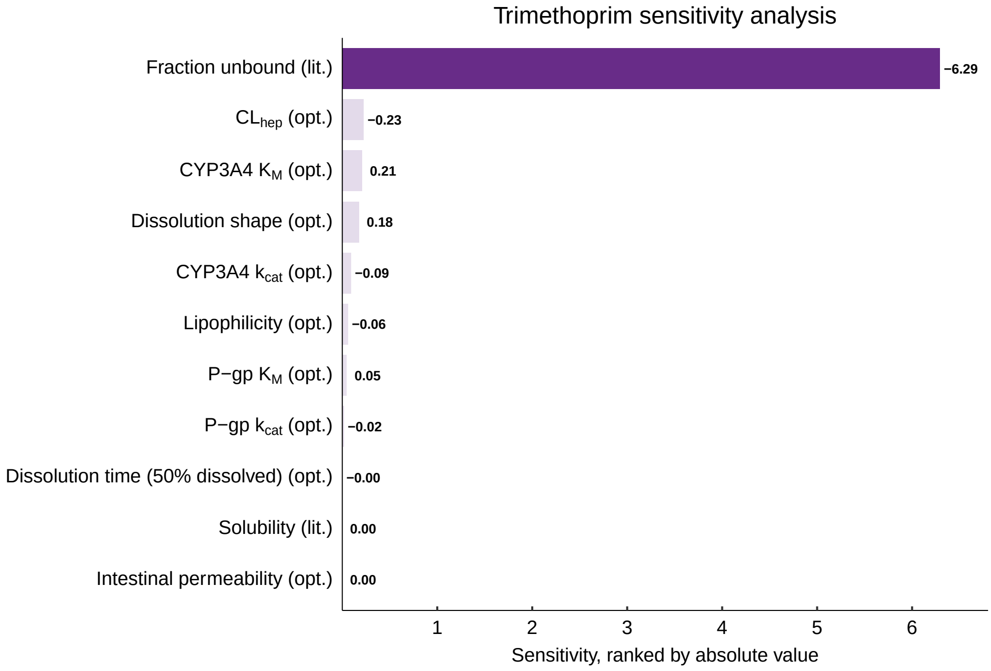

b) C_{max}

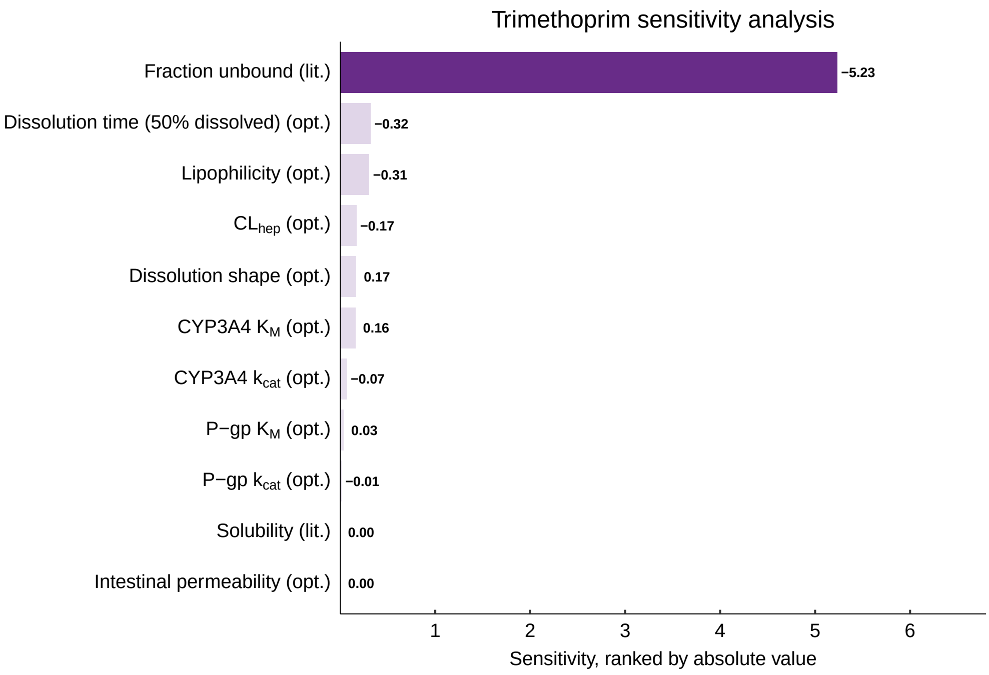

c) t_{max}

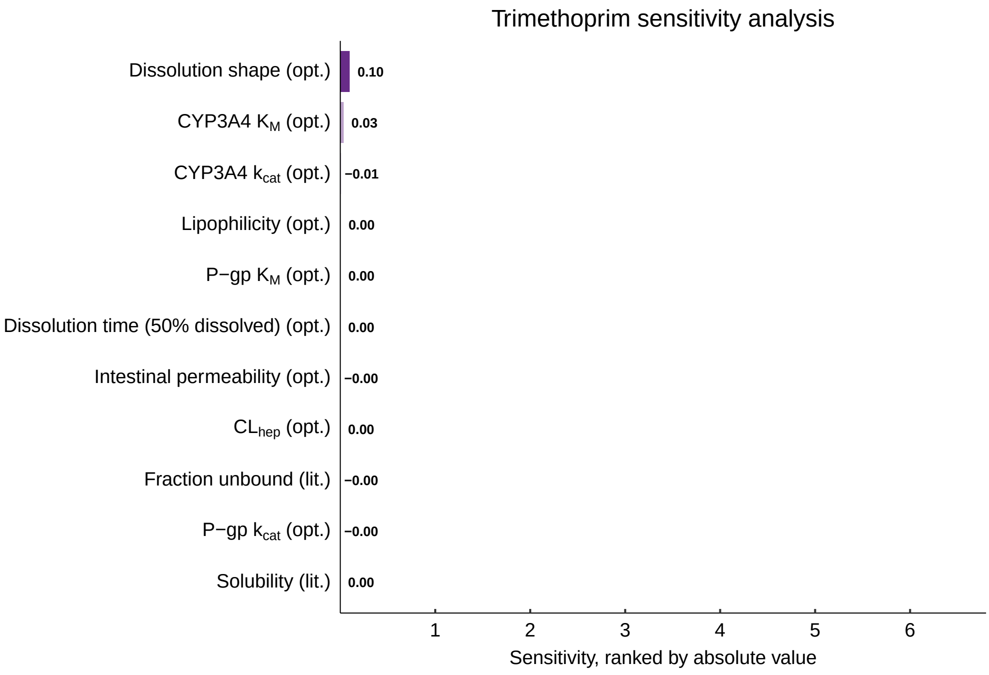

Figure S13: Sensitivity analysis of the trimethoprim model. Sensitivity of the model to single parameters, determined as change of the simulated (**a**) AUC_{0-12}, (**b**) C_{max} and (**c**) t_{max} at steady state of an oral 160 mg twice daily trimethoprim regimen. CL_{hep} hepatic metabolic clearance, *CYP* cytochrome P450, k_{cat} transport or catalytic rate constant, K_M Michaelis-Menten constant, *lit.* literature value, *opt.* optimized value, *P-gp* P-glycoprotein.

3 Trimethoprim-metformin DDI and DDGI

3.1 DDI and DDGI modeling

Metformin is listed by the FDA as the only recommended MATE and OCT2 substrate for clinical DDI studies and drug labeling [20]. The trimethoprim-metformin DDI and DDGI were predicted using literature values for all interaction constants without further optimization. The competitive inhibition of MATE1, OCT1 and OCT2 by trimethoprim was modeled applying K_i values of 4.45 µmol/L [21, 22, 65–67], 32.20 µmol/L [21, 22] and 47.82 µmol/L [21, 22, 65, 68, 69], respectively, determined using transporter expressing CHO or HEK 293 cells, without correction for fraction unbound in the incubation (fu_{inc}). The implemented model processes are visualized in Figure S14. The interaction parameters are listed in the trimethoprim drug-dependent parameter Table S2 and the parameters of the applied metformin model [6], including different OCT2 k_{cat} values to describe the *SLC22A2 808G>T* polymorphism, are reproduced in Table S8.

The population predictions of metformin plasma concentration-time profiles before and during trimethoprim co-administration, compared to observed data, are shown in semilogarithmic (Figure S15) and linear plots (Figure S16). The correlation of predicted and observed DDI and DDGI AUC_{last} and C_{max} ratios is shown in Figure S17. Table S9 lists the corresponding predicted and observed DDI and DDGI AUC_{last} and C_{max} ratios as well as GMFE values (mean GMFEs of 1.08 and 1.14, respectively).

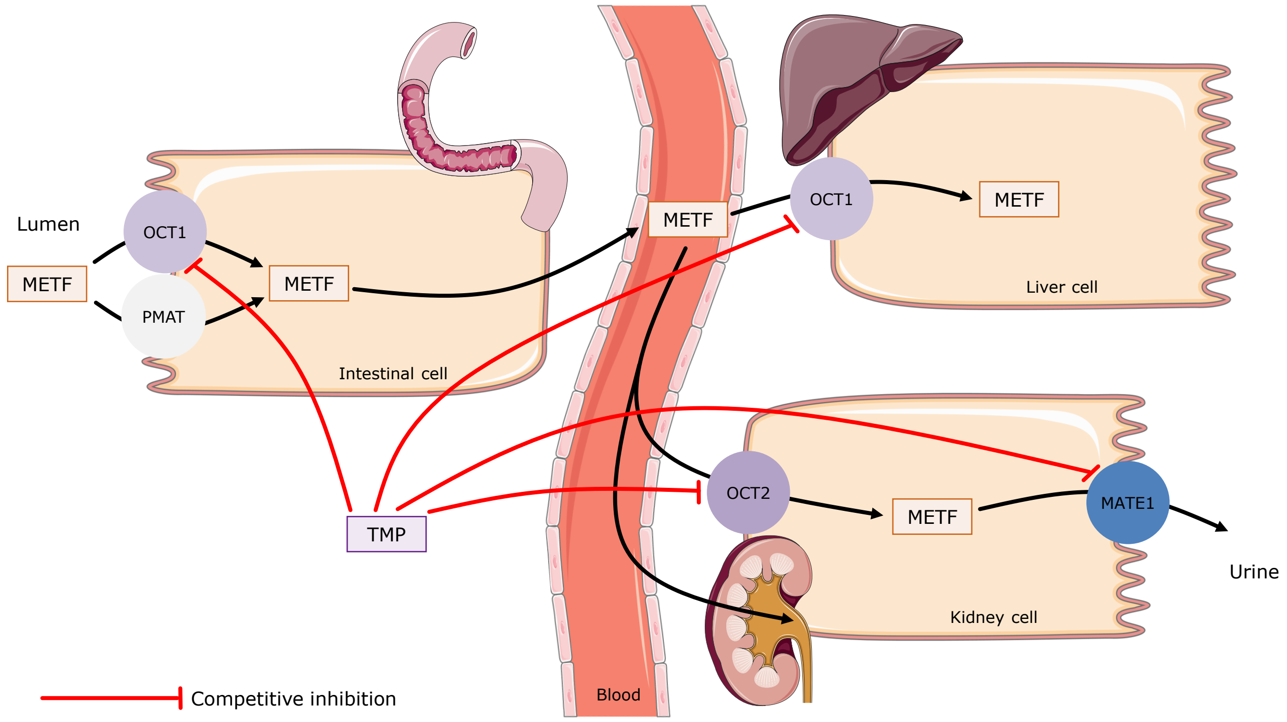

Figure S14: Trimethoprim-metformin DDI model processes. Drawings by Servier, licensed under CC BY 3.0. *METF* metformin, *MATE* multidrug and toxin extrusion protein, *OCT* organic cation transporter, *PMAT* plasma membrane monoamine transporter, *TMP* trimethoprim.

3.2 Clinical studies

Details on the clinical studies investigating the trimethoprim-metformin DDI and DDGI are given in Table S7.

Table S7: Clinical studies investigating the trimethoprim-metformin DDI and DDGI

Perpetrator		Victim										
Route	Dose	Route	Dose	Dose gap	n	Females [%]	Age [years]	Weight [kg]	Height [cm]	BMI [kg/m^2]	*SLC22A2* [a]	Reference
Trimethoprim		*Metformin*										
DDI												
po, bid, D1-5	200 mg	po, bid, D4-5	850 mg	0 h	12	33	21-38 (26.7)	55-84 (69.5)	-	20-25 (22.1)	-	Müller 2015 [22]
po, bid, D4-10	200 mg	po, tid, D1-10	500 mg	0.5 h	6	50	(32)	(71.6)	(175)	-	wildtype	Grün 2013 [2]
DDGI												
po, bid, D4-10	200 mg	po, tid, D1-10	500 mg	0.5 h	5	40	(33)	(72.2)	(171)	-	*808GT*	Grün 2013 [2]

Values for age, weight, height and BMI are reported as range (mean). [a] genotype. - not given, *bid* twice daily, *BMI* body mass index, *D* day of administration, *DDI* drug-drug interaction, *DDGI* drug-drug-gene interaction, *n* number of individuals studied, *po* oral, *SLC* solute carrier family member, *tid* three times daily.

3.3 Metformin drug-dependent parameters

Table S8: Drug-dependent parameters of the metformin PBPK model [6]

Parameter	Value	Unit	Source	Literature	Reference	Description
Metformin						
MW	129.16	g/mol	Literature	129.16	[57]	Molecular weight
pKa_1 (base)	2.80		Literature	2.80	[73]	Acid dissociation constant
pKa_2 (base)	11.50		Literature	11.50	[73]	Acid dissociation constant
Solubility (pH 6.8)	350.90	g/L	Literature	350.90	[73]	Solubility
logP	-1.43		Literature	-1.43	[74]	Lipophilicity
fu	100	%	Literature	100	[75–77]	Fraction unbound plasma
B/P ratio	-		-	Time-dependent	[75]	Blood/plasma concentration ratio
MATE1 K_M	283.00	µmol/L	Literature	283.00	[78]	Michaelis-Menten constant
MATE1 k_{cat}	165.69	1/min	Optimized	-	-	Transport rate constant
OCT1 K_M	1180.00	µmol/L	Literature	1180.00	[4]	Michaelis-Menten constant
OCT1 k_{cat}	641.19	1/min	Optimized	-	-	Transport rate constant
OCT2 K_M	810.00	µmol/L	Literature	810.00	[4]	Michaelis-Menten constant
OCT2 (*SLC22A2 808G*) k_{cat}	5.17E+04	1/min	Optimized	-	-	Transport rate constant
OCT2 (*SLC22A2 808T*) k_{cat}	1.38E+05	1/min	Optimized	-	-	Transport rate constant
PMAT K_M	367.57	µmol/L	Optimized	1320.00	[79]	Michaelis-Menten constant
PMAT k_{cat}	76.47	1/min	Optimized	-	-	Transport rate constant
PMAT Hill	3.00		Literature	2.64	[79]	Hill coefficient
GFR fraction	1		Assumed	-	-	Fraction of filtered drug in the urine
EHC continuous fraction	1		Assumed	-		Fraction of bile continually released
Partition coefficients	Diverse		Calculated	PK-Sim	[80]	Cell to plasma partition coefficients
Cellular permeability	2.30E-04	cm/min	Calculated	CDS norm.	[16]	Permeability into the cellular space
Intestinal permeability	8.49E-07	cm/min	Optimized	1.87E-07	Calculated	Transcellular intestinal permeability
Basolat. small int. permeability	1.16E-05	cm/min	Optimized	1.11E-06	Calculated	Basolateral permeability out of the mucosa
Basolat. large int. permeability	0	cm/min	Assumed	1.11E-06	Calculated	Basolateral permeability out of the mucosa
Formulation	Weibull [a]		Literature/Optimized	-	[72]	Formulation used in predictions

[a] Weibull fasted: Weibull function with a dissolution time of 7.90 minutes (50% dissolved) and a dissolution shape of 1.36 (both extracted from literature [72]), Weibull fed: Weibull function with a dissolution time of 7.90 minutes and a dissolution shape of 0.11 (both optimized). *basolat.* basolateral, *CDS norm.* charge-dependent Schmitt normalized to PK-Sim calculation method, *EHC* enterohepatic circulation, *GFR* glomerular filtration rate, *int.* intestinal, *MATE* multidrug and toxin extrusion protein, *OCT* organic cation transporter, *PK-Sim* PK-Sim standard calculation method, *PMAT* plasma membrane monoamine transporter.

3.4 Profiles

3.4.1 Semilogarithmic plots - Plasma

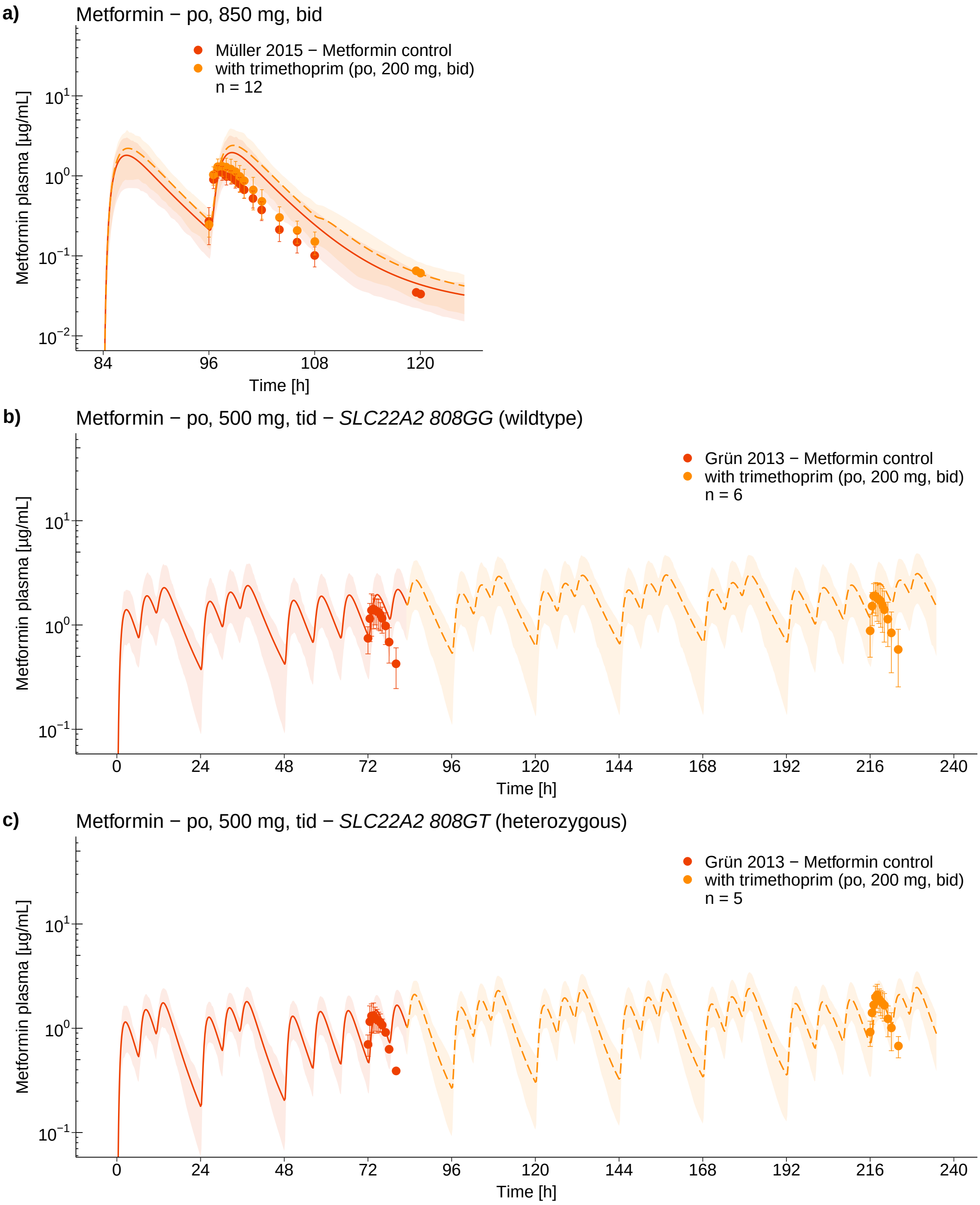

Figure S15: Metformin plasma concentration-time profiles before and during trimethoprim co-administration (semilogarithmic). Profiles (**a-b**) show the trimethoprim-metformin DDI; the profile (**c**) shows the DDGI. Observed data are shown as circles ± standard deviation. Population simulation arithmetic means are shown as lines (solid lines: victim drug alone; dashed lines: victim drug during perpetrator co-administration); the shaded areas represent the 68% population prediction intervals. Details on dosing regimens, study populations and literature references are listed in Table S7. Predicted and observed DDI and DDGI AUC_{last} and C_{max} ratios are summarized in Table S9. *bid* twice daily, *n* number of individuals studied, *po* oral, *SLC* solute carrier family member, *tid* three times daily.

3.4.2 Linear plots - Plasma

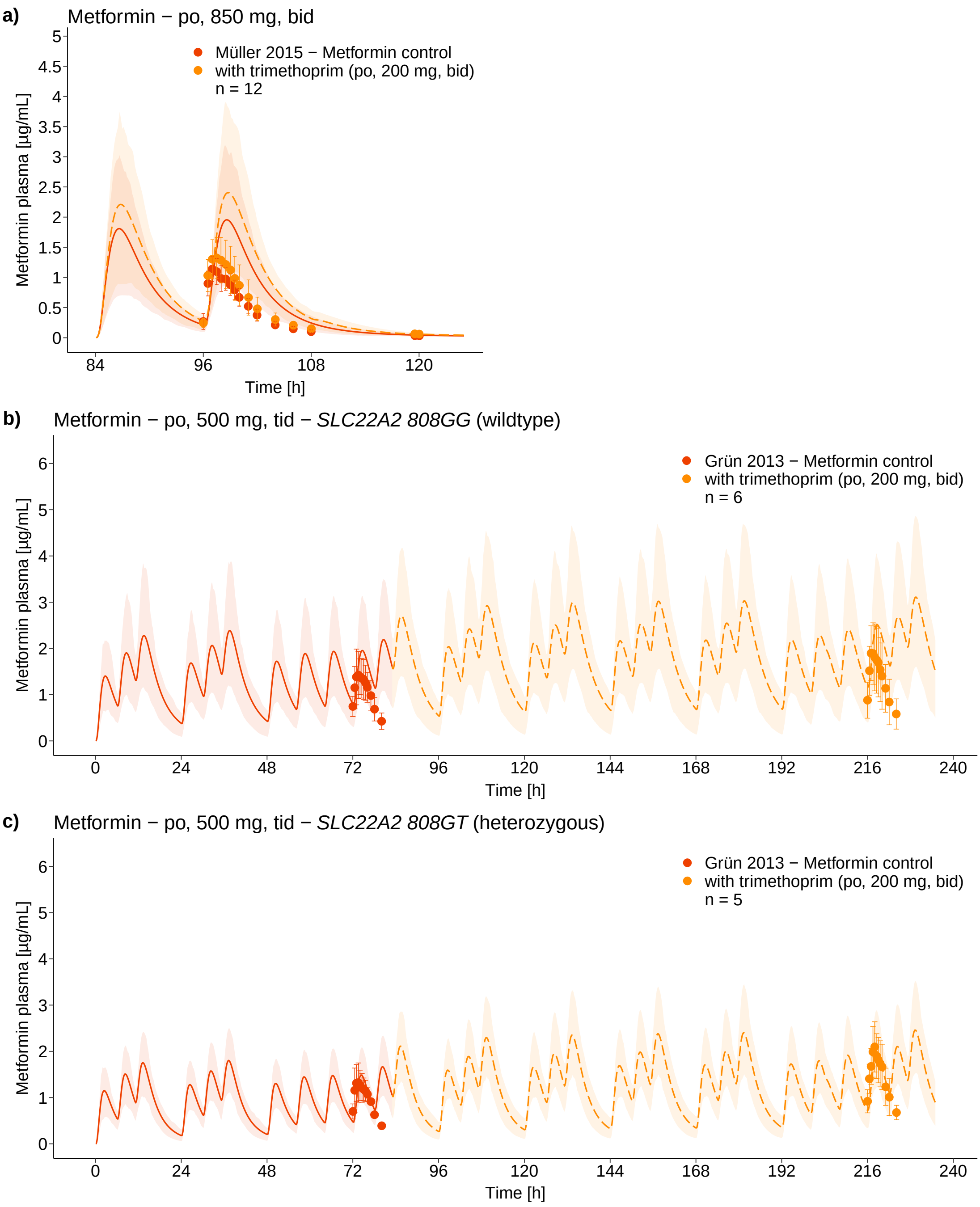

Figure S16: Metformin plasma concentration-time profiles before and during trimethoprim co-administration (linear). Profiles (**a-b**) show the trimethoprim-metformin DDI; the profile (**c**) shows the DDGI. Observed data are shown as circles ± standard deviation. Population simulation arithmetic means are shown as lines (solid lines: victim drug alone; dashed lines: victim drug during perpetrator co-administration); the shaded areas represent the 68% population prediction intervals. Details on dosing regimens, study populations and literature references are listed in Table S7. Predicted and observed DDI and DDGI AUC_{last} and C_{max} ratios parameters are summarized in Table S9. *bid* twice daily, *n* number of individuals studied, *po* oral, *SLC* solute carrier family member, *tid* three times daily.

3.5 DD(G)I model performance evaluation

3.5.1 DDI and DDGI AUC_{last} and C_{max} ratio goodness-of-fit plots

a) DDI and DDGI AUC_{last} ratios

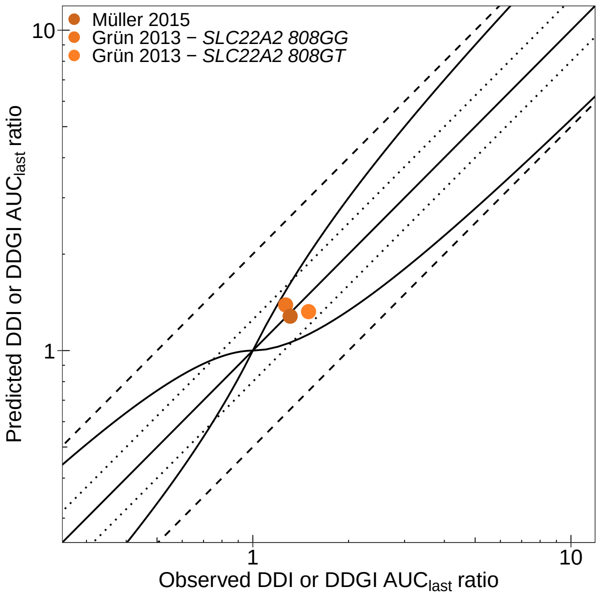

b) DDI and DDGI C_{max} ratios

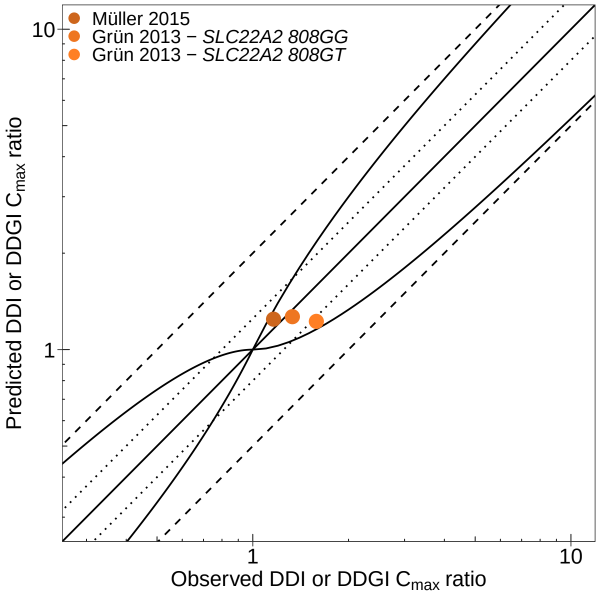

Figure S17: Comparison of predicted to the corresponding observed metformin DDI or DDGI (**a**) AUC_{last} and (**b**) C_{max} ratios of the trimethoprim-metformin DDI and DDGI. The solid straight line line marks the line of identity, dotted lines indicate 1.25-fold and dashed lines indicate 2-fold deviation. The curved lines show the prediction success limits suggested by Guest et al. [81]. Details on the study protocols and the predicted and observed DDI and DDGI AUC_{last} and C_{max} ratios are given in Tables S7 and S9, respectively. *SLC* solute carrier family member.

3.5.2 Predicted and observed DDI and DDGI AUC_{last} and C_{max} ratios with mean GMFE values and ranges

Table S9: Predicted and observed trimethoprim-metformin DDI and DDGI AUC_{last} and C_{max} ratios

Perpetrator		Victim			DDI or DDGI AUC_{last} ratio			DDI or DDGI C_{max} ratio				
Route	Dose	Route	Dose	t_{last}	Pred	Obs	Pred/Obs	Pred	Obs	Pred/Obs	*SLC22A2* [a]	Reference
Trimethoprim		*Metformin*										
DDI												
po, bid, D1-5	200 mg	po, bid, D4-5	850 mg	24 h	1.28	1.31	0.98	1.24	1.16	1.07	-	Müller 2015 [22]
po, bid, D4-10	200 mg	po, tid, D1-10	500 mg	6 h	1.39	1.27	1.10	1.27	1.33	0.95	wildtype	Grün 2013 [2]
**mean GMFE (range)**							**1.06 (1.02 – 1.10)**<br>**2/2 with GMFE ≤ 2**			**1.06 (1.05 – 1.07)**<br>**2/2 with GMFE ≤ 2**		
DDGI												
po, bid, D4-10	200 mg	po, tid, D1-10	500 mg	6 h	1.32	1.50	0.88	1.22	1.58	0.77	*808GT*	Grün 2013 [2]
**GMFE**							**1.13**<br>**1/1 with GMFE ≤ 2**			**1.29**<br>**1/1 with GMFE ≤ 2**		
**Overall GMFE (range)**							**1.08 (1.02 – 1.13)**<br>**3/3 with GMFE ≤ 2**			**1.14 (1.05 – 1.29)**<br>**3/3 with GMFE ≤ 2**		

[a] genotype. *AUC* area under the concentration-time curve, *bid* twice daily, C_{max} peak plasma concentration, *D* day of administration, *DDI* drug-drug interaction, *DDGI* drug-drug-gene interaction, *GMFE* geometric mean fold error, *obs* observed, *po* oral, *pred* predicted, t_{last} time of the last concentration measurement, *tid* three times daily.

4 Trimethoprim-repaglinide DDI

4.1 DDI modeling

Repaglinide is mainly metabolized by CYP2C8 and recommended by the FDA as sensitive CYP2C8 index substrate for the use in clinical DDI studies [20]. The trimethoprim-repaglinide DDI was predicted using a literature value for the interaction constant without further optimization. The competitive inhibition of CYP2C8 by trimethoprim was modeled with K_i = 4.85 µmol/L [70], determined using human liver microsomes without correction for fu_{inc}, as the calculated fu_{inc} is almost 100% according to [82]. The implemented model processes are visualized in Figure S18. The interaction parameters are listed in the trimethoprim drug-dependent parameter Table S2 and the parameters of the applied repaglinide model [17] are reproduced in Table S11.

The population predictions of repaglinide plasma concentration-time profiles before and during trimethoprim co-administration, compared to observed data, are shown in semilogarithmic and linear plots (Figure S19). The correlation of predicted and observed DDI AUC_{last} and C_{max} ratios is shown in Figure S20. Table S12 lists the corresponding predicted and observed DDI AUC_{last} and C_{max} ratios as well as GMFE values (GMFEs of 1.27 and 1.11, respectively).

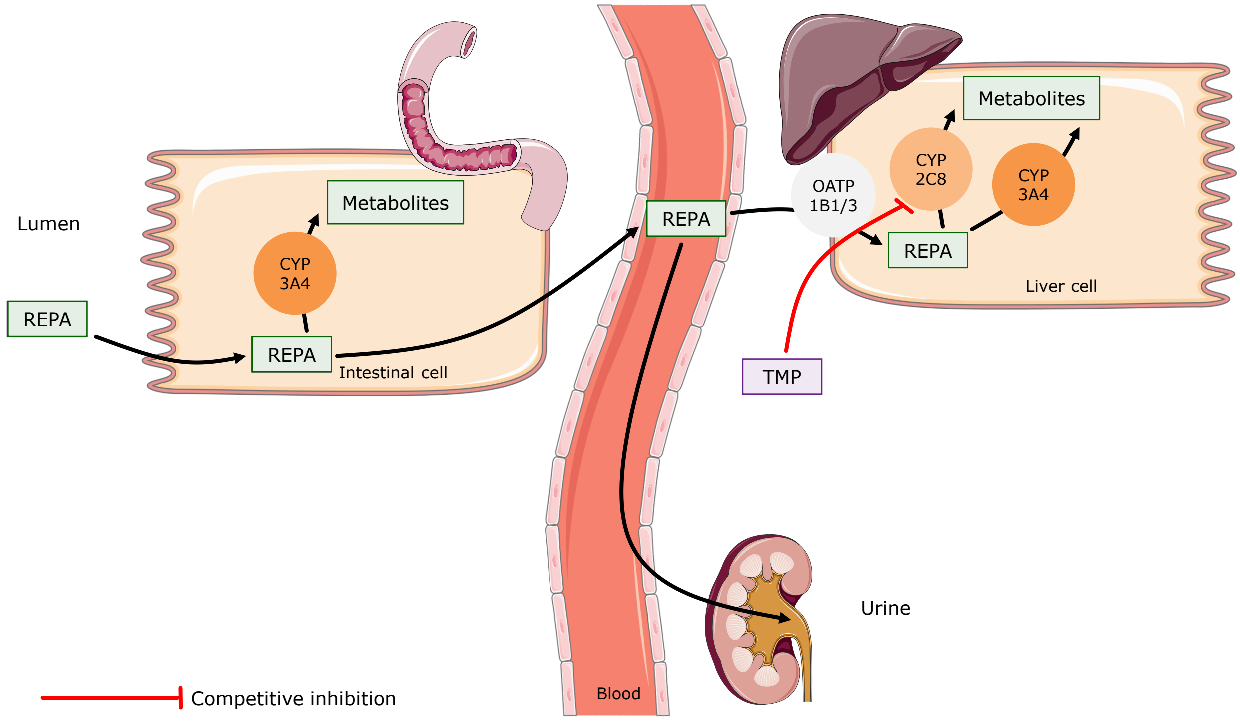

Figure S18: Trimethoprim-repaglinide DDI model processes. Drawings by Servier, licensed under CC BY 3.0. *CYP* cytochrome P450, *OATP* organic-anion-transporting polypeptide, *REPA* repaglinide, *TMP* trimethoprim.

4.2 Clinical studies

Details on the clinical study investigating the trimethoprim-repaglinide DDI are given in Table S10.

Table S10: Clinical studies investigating the trimethoprim-repaglinide DDI

Perpetrator		Victim									
Route	Dose	Route	Dose	Dose gap	n	Females [%]	Age [years]	Weight [kg]	Height [cm]	BMI [kg/m^2]	Reference
Trimethoprim		*Repaglinide*									
po, bid, D1-3	160 mg	po, sd, D3	0.25 mg	1 h	9	11	19-23	62-97	-	-	Niemi 2004a [83]

Values for age and weight are reported as range. - not given, *bid* twice daily, *BMI* body mass index, *D* day of administration, *DDI* drug-drug interaction, *n* number of individuals studied, *po* oral, *sd* single dose.

4.3 Repaglinide drug-dependent parameters

Table S11: Drug-dependent parameters of the repaglinide PBPK model [17]

Parameter	Value	Unit	Source	Literature	Reference	Description
Repaglinide						
MW	452.60	g/mol	Literature	452.60	[59]	Molecular weight
pKa_1 (acid)	4.16		Literature	3.68, 3.96, 4.16, 4.19	[57, 85, 86]	Acid dissociation constant
pKa_2 (base)	6.01		Literature	4.82, 5.78, 6.01, 6.20	[57, 85, 86]	Acid dissociation constant
Solubility (pH 7.4)	0.14	g/L	Literature	0.14	[87]	Solubility
logP	2.72		Optimized	3.95, 3.98, 4.87, 5.05	[57, 85, 86]	Lipophilicity
fu	2.9	%	Optimized	1.5, 2.6, 3.6	[88–90]	Fraction unbound plasma
OATP1B1 K_M	12.8	µmol/L	Literature	12.8 [a]	[91]	Michaelis-Menten constant
OATP1B1 k_{cat}	1600.24	1/min	Optimized	-	-	Transport rate constant
OATP1B3 K_M	12.8	µmol/L	Literature	12.8 [a]	[91]	Michaelis-Menten constant
OATP1B3 k_{cat}	551.24	1/min	Optimized	-	-	Transport rate constant
CYP2C8 K_M	2.8	µmol/L	Literature	2.8	[92]	Michaelis-Menten constant
CYP2C8 k_{cat}	4.56	1/min	Optimized	-	-	Catalytic rate constant
CYP3A4 K_M	15.6	µmol/L	Literature	15.6	[92]	Michaelis-Menten constant
CYP3A4 k_{cat}	0.86	1/min	Optimized	-	-	Catalytic rate constant
GFR fraction	1		Assumed	-	-	Fraction of filtered drug in the urine
EHC continuous fraction	1		Assumed	-	-	Fraction of bile continually released
Partition coefficients	Diverse		Calculated	Schmitt	[93]	Cell to plasma partition coefficients
Cellular permeability	0.04	cm/min	Optimized	CDS	[16]	Permeability into the cellular space
Intestinal permeability	2.02E-05	cm/min	Optimized	9.38E-06	Calculated	Transcellular intestinal permeability
Formulation	Tablet [b]		Literature	-	[84]	Formulation used in predictions

[a] repaglinide hepatic uptake unbound affinity constant, [b] tablet dissolution profile from literature [84]. *CDS* charge-dependent Schmitt calculation method, *CYP* cytochrome P450, *EHC* enterohepatic circulation, *GFR* glomerular filtration rate, *OATP* organic-anion-transporting polypeptide, *Schmitt* Schmitt calculation method.

4.4 Profiles

4.4.1 Semilogarithmic and linear plots - Plasma

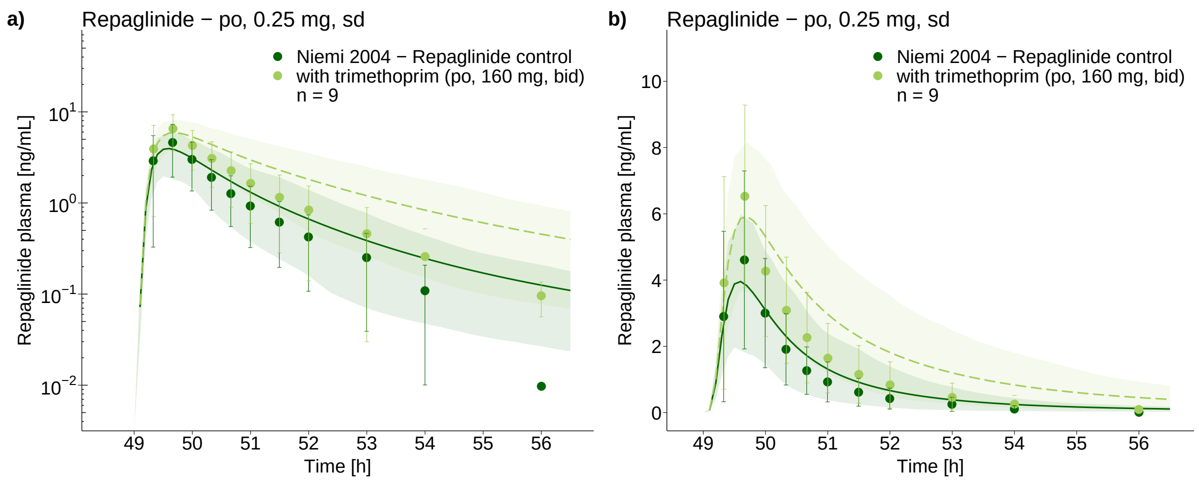

Figure S19: Repaglinide plasma concentration-time profiles before and during trimethoprim co-administration, shown in (**a**) semilogarithmic and (**b**) linear plots. Observed data are shown as circles ± standard deviation. Population simulation arithmetic means are shown as lines (solid lines: victim drug alone; dashed lines: victim drug during perpetrator co-administration); the shaded areas represent the 68% population prediction intervals. Details on dosing regimens, study populations and literature references are listed in Table S10. Predicted and observed DDI AUC_{last} and C_{max} ratios are summarized in Table S12. *bid* twice daily, *n* number of individuals studied, *po* oral, *sd* single dose.

4.5 DDI model performance evaluation

4.5.1 DDI AUC_{last} and C_{max} ratio goodness-of-fit plots

a) DDI AUC_{last} ratio

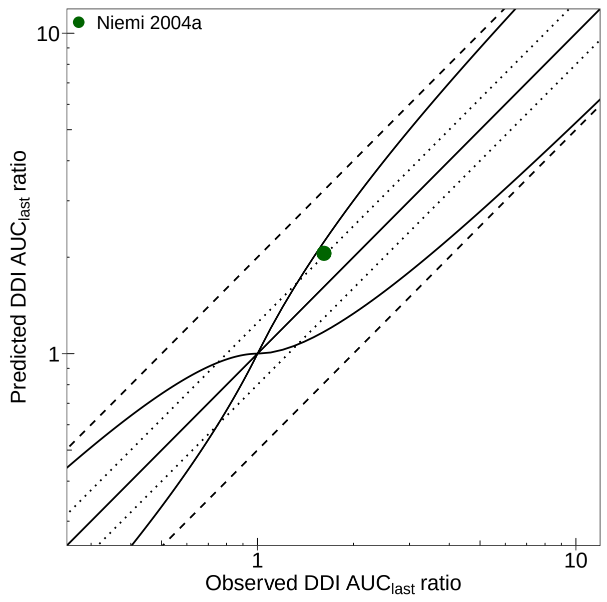

b) DDI C_{max} ratio

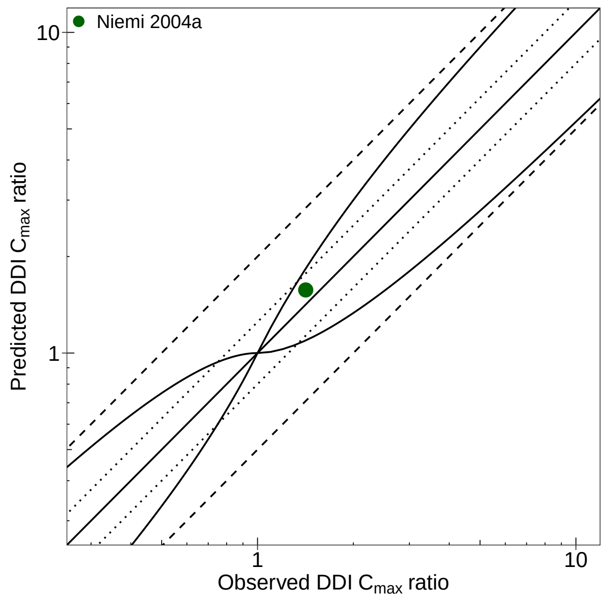

Figure S20: Comparison of predicted the corresponding observed repaglinide DDI (**a**) AUC_{last} and (**b**) C_{max} ratios of the trimethoprim-repaglinide DDI. The solid straight line marks the line of identity, dotted lines indicate 1.25-fold and dashed lines indicate 2-fold deviation. The curved lines show the prediction success limits suggested by Guest et al. [81]. Details on the study protocols and the predicted and observed DDI AUC_{last} and C_{max} ratios are given in Tables S10 and S12, respectively.

4.5.2 Predicted and observed DDI $\mathbf{AUC_{last}}$ and $\mathbf{C_{max}}$ ratios with GMFE values

Table S12: Predicted and observed trimethoprim-repaglinide DDI AUC_{last} and C_{max} ratios

Perpetrator		Victim			DDI AUC_{last} ratio			DDI C_{max} ratio			
Route	Dose	Route	Dose	t_{last}	Pred	Obs	Pred/Obs	Pred	Obs	Pred/Obs	Reference
Trimethoprim		*Repaglinide*									
po, bid, D1-3	160 mg	po, sd, D3	0.25 mg	7 h	2.06	1.62	1.27	1.57	1.42	1.11	Niemi 2004a [83]
**GMFE**							**1.27**<br>**1/1 with GMFE ≤ 2**			**1.11**<br>**1/1 with GMFE ≤ 2**	

AUC area under the concentration-time curve, *bid* twice daily, C_{max} peak plasma concentration, *D* day of administration, *DDI* drug-drug interaction, *GMFE* geometric mean fold error, *obs* observed, *po* oral, *pred* predicted, *sd* single dose, t_{last} time of the last concentration measurement.

5 Trimethoprim-pioglitazone DDI and DDGI

5.1 DDI and DDGI modeling

Pioglitazone is mainly metabolized by CYP2C8 and recommended by the FDA as moderately sensitive CYP2C8 substrate for the use in clinical DDI studies [20]. The trimethoprim-pioglitazone DDI and DDGI were predicted using literature values for all interaction constants without further optimization. The competitive inhibition of CYP2C8 by trimethoprim was modeled with $K_i = 4.85$ µmol/L [70], determined using human liver microsomes without correction for fu_{inc}, as the calculated fu_{inc} is almost 100% according to [82]. The same K_i was assumed for CYP2C8 wildtype and *3 variants. The implemented model processes are visualized in Figure S21. The interaction parameters are listed in the trimethoprim drug-dependent parameter Table S2 and the parameters of the applied pioglitazone model [17], including K_M and k_{cat} values to model the CYP2C8 polymorphism, are reproduced in Table S14.

The population predictions of pioglitazone plasma concentration-time profiles before and during trimethoprim co-administration, compared to observed data, are shown in semilogarithmic (Figure S22) and linear plots (Figure S23). For the DDGI, no observed plasma concentration-time profiles are provided in the study report (only for the DGI = pioglitazone without trimethoprim), but the reported values for observed $AUC_{0-\infty}$ were taken from the publication and compared to predicted $AUC_{0-\infty}$ values. The correlation of predicted and observed DDI and DDGI AUC_{last} and C_{max} ratios is shown in Figure S24. Table S15 lists the corresponding predicted and observed DDI and DDGI AUC_{last} and C_{max} ratios as well as GMFE values (mean GMFEs of 1.32 and 1.04, respectively).

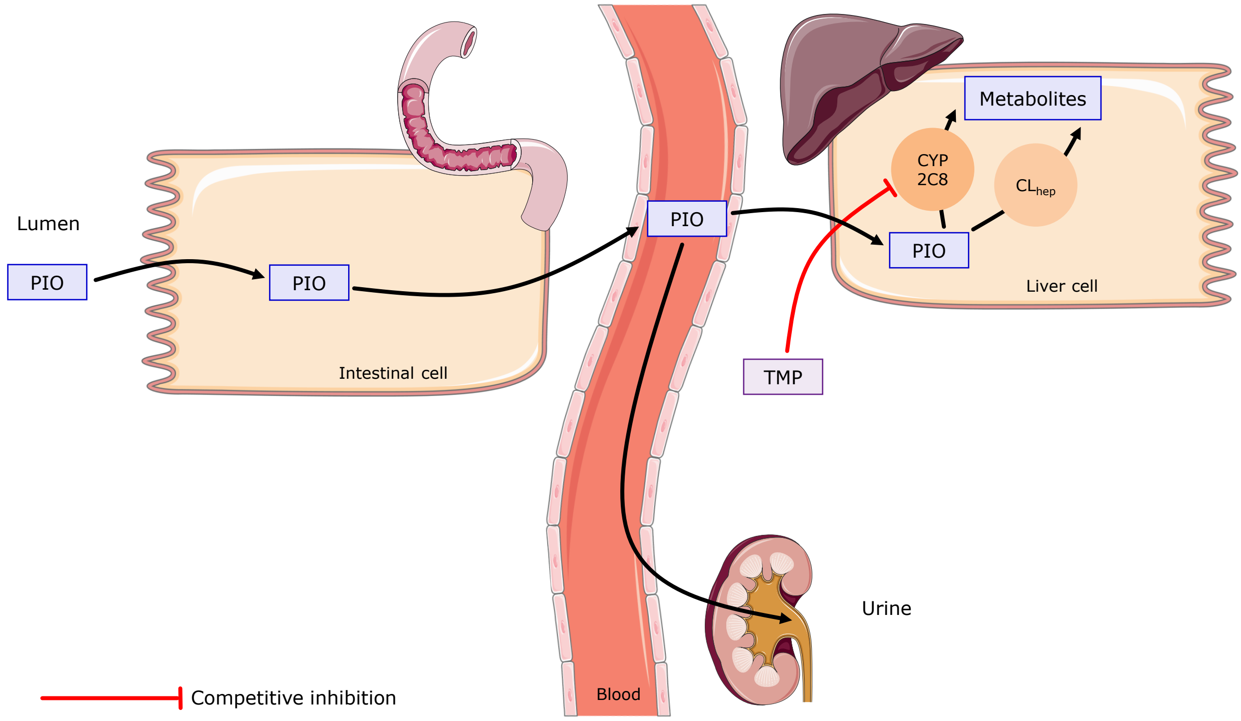

Figure S21: Trimethoprim-pioglitazone DDI model processes. Drawings by Servier, licensed under CC BY 3.0. CL_{hep} hepatic metabolic clearance, *CYP* cytochrome P450, *PIO* pioglitazone, *TMP* trimethoprim.

5.2 Clinical studies

Details on the clinical studies investigating the trimethoprim-pioglitazone DDI and DDGI are given in Table S13.

Table S13: Clinical studies investigating the trimethoprim-pioglitazone DDI and DDGI

Perpetrator		Victim										
Route	Dose	Route	Dose	Dose gap	n	Females [%]	Age [years]	Weight [kg]	Height [cm]	BMI [kg/m^2]	*CYP2C8* [a]	Reference
Trimethoprim		*Pioglitazone*										
DDI												
po, bid, D1-6	160 mg	po, bid, D3	15 mg	1 h	16	50	19-25 (21)	44-93 (68)	-	18-27 (22)	**1/*1*: n = 8<br>**1/*3*: n = 5<br>**3/*3*: n = 3	Tornio 2008 [10]
DDGI												
po, bid, D1-6	160 mg	po, bid, D3	15 mg	1 h	8	50	19-25 (21)	56-93 (70)	-	18-27 (22)	**1/*1*	Tornio 2008 [10]
po, bid, D1-6	160 mg	po, bid, D3	15 mg	1 h	5	40	19-25 (21)	60-83 (67)	-	19-23 (22)	**1/*3*	Tornio 2008 [10]
po, bid, D1-6	160 mg	po, bid, D3	15 mg	1 h	3	67	19-25 (21)	44-79 (64)	-	18-25 (22)	**3/*3*	Tornio 2008 [10]

Values for age, weight and BMI are reported as range (mean). [a] genotype. - not given, *bid* twice daily, *BMI* body mass index, *CYP* cytochrome P450, *D* day of administration, *DDI* drug-drug interaction, *DDGI* drug-drug-gene interaction, *n* number of individuals studied, *po* oral.

5.3 Pioglitazone drug-dependent parameters

Table S14: Drug-dependent parameters of the pioglitazone PBPK model [17]

Parameter	Value	Unit	Source	Literature	Reference	Description
Pioglitazone						
MW	356.40	g/mol	Literature	356.40	[59]	Molecular weight
pKa_1 (base)	5.80		Literature	5.80	[95]	Acid dissociation constant
pKa_2 (acid)	6.40		Literature	6.40	[95]	Acid dissociation constant
Solubility (pH 6.5)	0.02	g/L	Literature	0.01, 0.02	[87, 96]	Solubility
logP	2.81		Optimized	3.31	[97]	Lipophilicity
fu	0.21	%	Optimized	< 1	[95]	Fraction unbound plasma
CYP2C8 K_M	21.0	µmol/L	Literature	21.0 [a]	[11]	Michaelis-Menten constant
CYP2C8 k_{cat}	68.09	1/min	Optimized	-	-	Catalytic rate constant
CYP2C8 (*CYP2C8*1*) K_M	21.0	µmol/L	Literature	21.0 [a]	[11]	Michaelis-Menten constant
CYP2C8 (*CYP2C8*1*) k_{cat}	84.79	1/min	Optimized	-	-	Catalytic rate constant
CYP2C8 (*CYP2C8*3*) K_M	10.0	µmol/L	Literature	10.0	[11]	Michaelis-Menten constant
CYP2C8 (*CYP2C8*3*) k_{cat}	104.82	1/min	Optimized	-	-	Catalytic rate constant
CL_{hep}	2.14	1/min	Optimized	-	-	Hepatic metabolic clearance
GFR fraction	1		Assumed	-	-	Fraction of filtered drug in the urine
EHC continuous fraction	1		Assumed	-	-	Fraction of bile continually released
Partition coefficients	Diverse		Calculated	Berezhkovskiy	[71]	Cell to plasma partition coefficients
Cellular permeability	9.10E-03	cm/min	Calculated	PK-Sim	[98]	Permeability into the cellular space
Intestinal permeability	4.38E-05	cm/min	Optimized	3.40E-05	Calculated	Transcellular intestinal permeability
Formulation	Tablet [b]		Literature	-	[94]	Formulation used in predictions

[a] same CYP2C8 Michaelis-Menten constant assumed for CYP2C8 genotype unknown and *CYP2C8*1*, [b] tablet dissolution profile from literature [94]. *Berezhkovskiy* Berezhkovskiy calculation method, CL_{hep} hepatic metabolic clearance, *CYP* cytochrome P450, *EHC* enterohepatic circulation, *GFR* glomerular filtration rate, *PK-Sim* PK-Sim standard calculation method.

5.4 Profiles

5.4.1 Semilogarithmic plots - Plasma

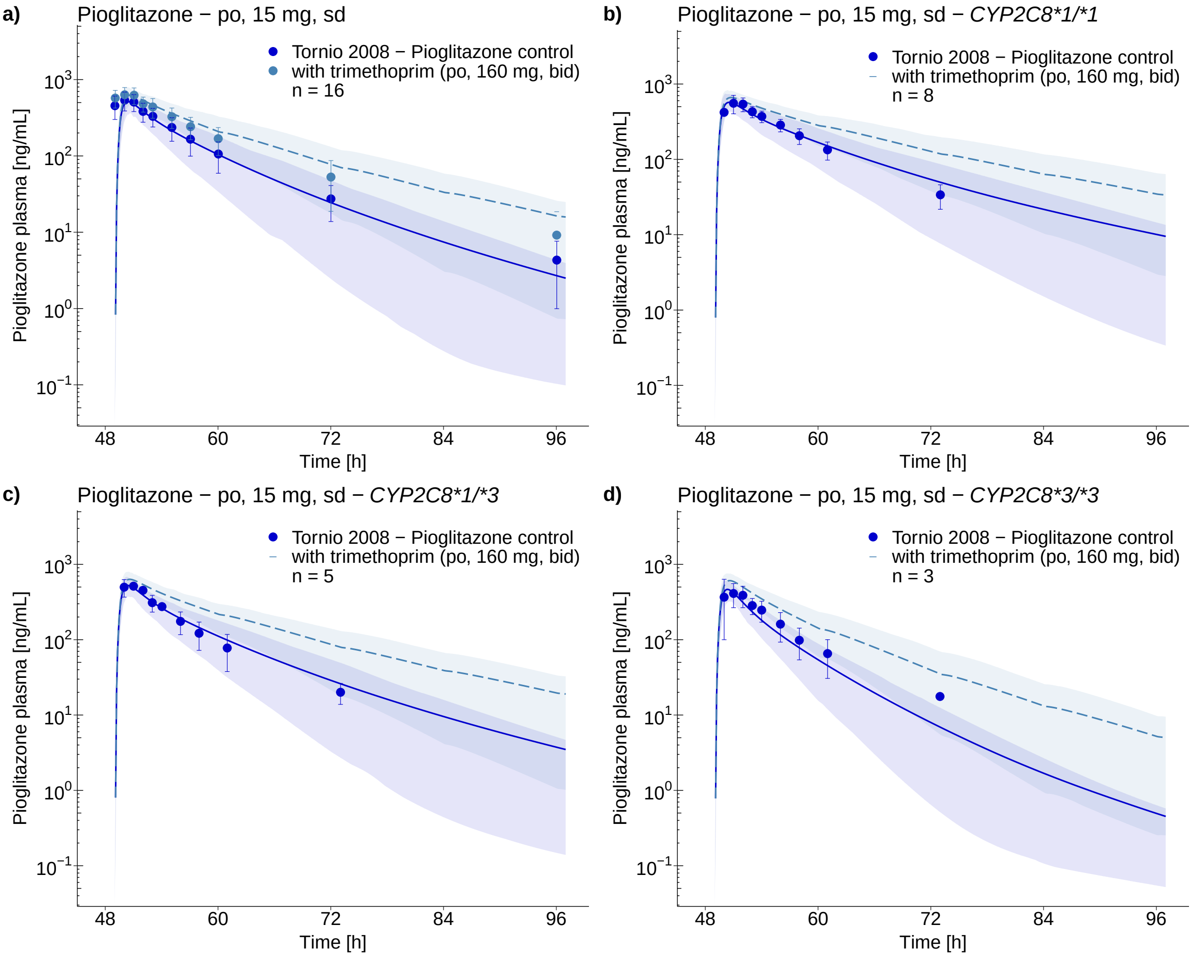

Figure S22: Pioglitazone plasma concentration-time profiles before and during trimethoprim co-administration (semilogarithmic). Profile (**a**) shows the trimethoprim-pioglitazone DDI; profiles (**b-d**) show the DDGI. Observed data are shown as circles ± standard deviation. Population simulation arithmetic means are shown as lines (solid lines: victim drug alone; dashed lines: victim drug during perpetrator co-administration); the shaded areas represent the 68% population prediction intervals. Details on dosing regimens, study populations and literature references are listed in Table S13. Predicted and observed DDI and DDGI AUC_{last} and C_{max} ratios are summarized in Table S15. *bid* twice daily, *CYP* cytochrome P450, *n* number of individuals studied, *po* oral, *sd* single dose.

5.4.2 Linear plots - Plasma

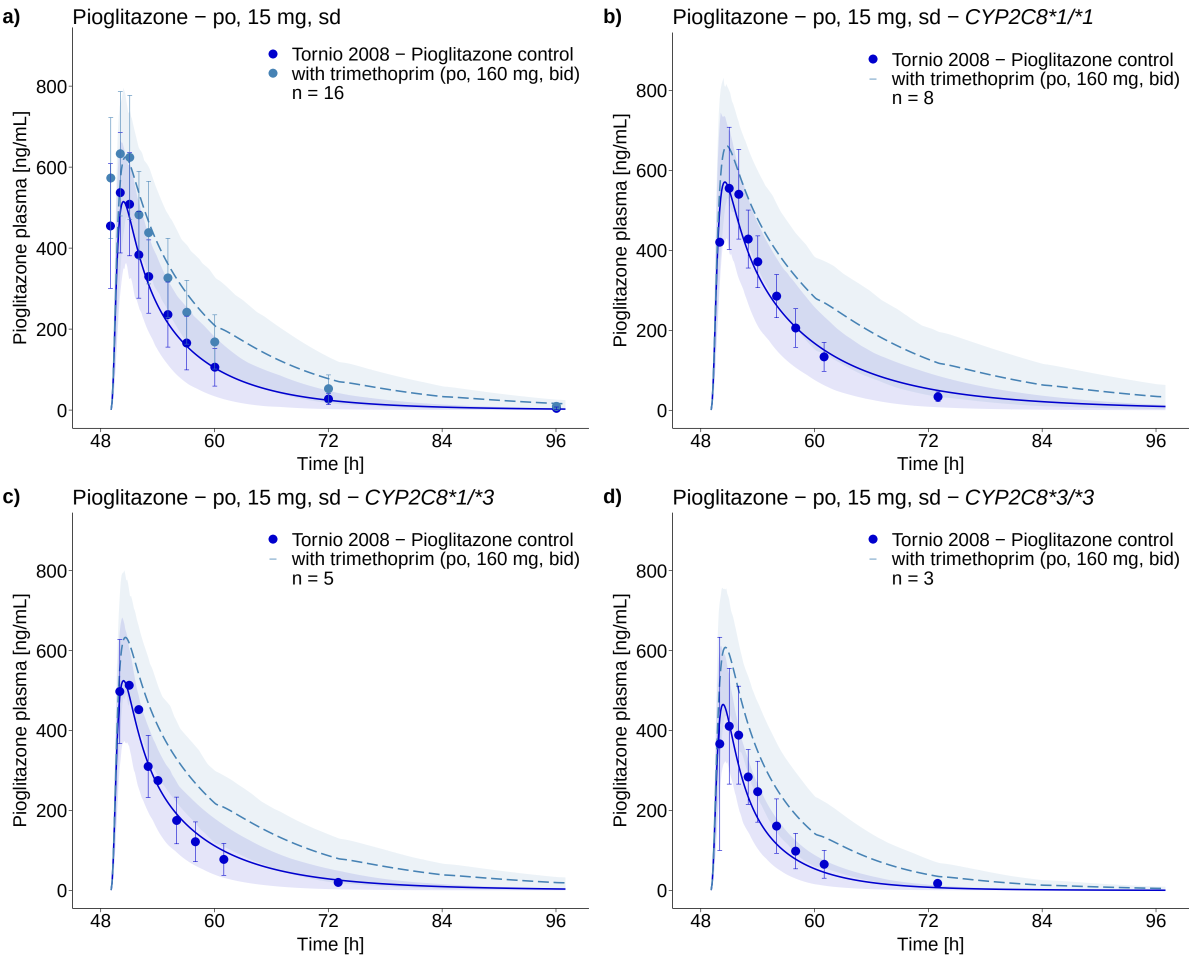

Figure S23: Pioglitazone plasma concentration-time profiles before and during trimethoprim co-administration (linear). Profile (**a**) shows the trimethoprim-pioglitazone DDI; profiles (**b-d**) show the DDGI. Observed data are shown as circles ± standard deviation. Population simulation arithmetic means are shown as lines (solid lines: victim drug alone; dashed lines: victim drug during perpetrator co-administration); the shaded areas represent the 68% population prediction intervals. Details on dosing regimens, study populations and literature references are listed in Table S13. Predicted and observed DDI and DDGI AUC_{last} and C_{max} ratios are summarized in Table S15. *bid* twice daily, *CYP* cytochrome P450, *n* number of individuals studied, *po* oral, *sd* single dose.

5.5 DD(G)I model performance evaluation

5.5.1 DDI and DDGI AUC_{last} and C_{max} ratio goodness-of-fit plots

a) DDI and DDGI AUC_{last} ratios

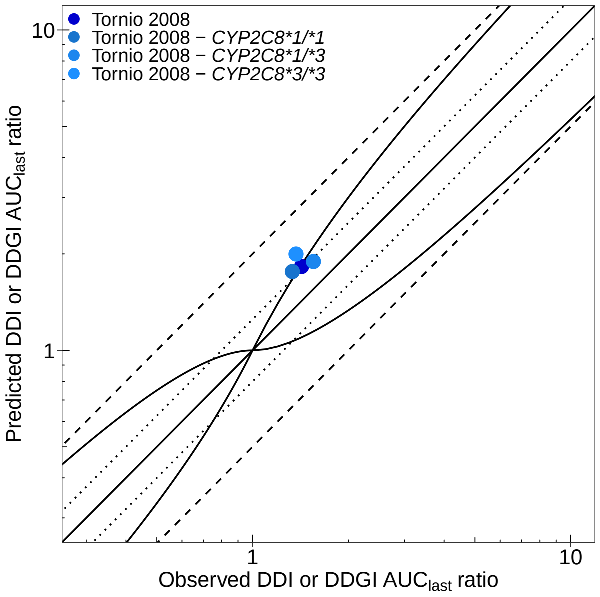

b) DDI and DDGI C_{max} ratios

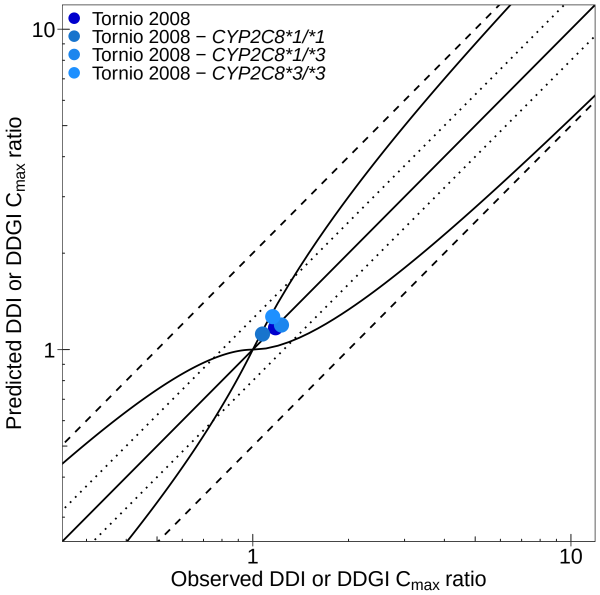

Figure S24: Comparison of predicted to the corresponding observed pioglitazone DDI or DDGI (**a**) AUC_{last} and (**b**) C_{max} ratios of the trimethoprim-pioglitazone DDI and DDGI. The solid straight line marks the line of identity, dotted lines indicate 1.25-fold and dashed lines indicate 2-fold deviation. The curved lines show the prediction success limits suggested by Guest et al. [81]. Details on the study protocols and the predicted and observed DDI and DDGI AUC_{last} and C_{max} ratios are given in Tables S13 and S15, respectively. *CYP* cytochrome P450.

5.5.2 Predicted and observed DDI and DDGI AUC_{last} and C_{max} ratios with mean GMFE values and ranges

Table S15: Predicted and observed trimethoprim-pioglitazone DDI and DDGI AUC_{last} and C_{max} ratios

Perpetrator		Victim			DDI or DDGI AUC_{last} ratio			DDI or DDGI C_{max} ratio				
Route	Dose	Route	Dose	t_{last}	Pred	Obs	Pred/Obs	Pred	Obs	Pred/Obs	*CYP2C8* [a]	Reference
Trimethoprim		*Pioglitazone*										
DDI												
po, bid, D1-6	160 mg	po, sd, D3	15 mg	48 h	1.83	1.43	1.28	1.17	1.18	0.99	**1/*1*: n = 5<br>**1/*3*: n = 5<br>**3/*3*: n = 3	Tornio 2008 [10]
**GMFE**							**1.28**<br>**1/1 with GMFE ≤ 2**			**1.01**<br>**1/1 with GMFE ≤ 2**		
DDGI												
po, bid, D1-6	160 mg	po, sd, D3	15 mg	∞	1.76	1.33	1.32	1.12	1.07	1.04	**1/*1*	Tornio 2008 [10]
po, bid, D1-6	160 mg	po, sd, D3	15 mg	∞	1.89	1.55	1.22	1.19	1.23	0.97	**1/*3*	Tornio 2008 [10]
po, bid, D1-6	160 mg	po, sd, D3	15 mg	∞	2.00	1.37	1.46	1.27	1.16	1.10	**3/*3*	Tornio 2008 [10]
**mean GMFE (range)**							**1.33 (1.22 – 1.46)**<br>**3/3 with GMFE ≤ 2**			**1.06 (1.03 – 1.10)**<br>**3/3 with GMFE ≤ 2**		
**Overall GMFE (range)**							**1.32 (1.22 – 1.46)**<br>**4/4 with GMFE ≤ 2**			**1.04 (1.01 – 1.10)**<br>**4/4 with GMFE ≤ 2**		

[a] genotype. *AUC* area under the concentration-time curve, *bid* twice daily, C_{max} peak plasma concentration, *D* day of administration, *DDI* drug-drug interaction, *DDGI* drug-drug-gene interaction, *GMFE* geometric mean fold error, *obs* observed, *po* oral, *pred* predicted, *sd* single dose, t_{last} time of the last concentration measurement.

6 Rifampicin-trimethoprim DDI

6.1 DDI modeling

Rifampicin is an inducer of P-gp and CYP enzymes [20, 99]. The rifampicin-trimethoprim DDI was modeled using interaction parameters, that have been established during the rifampicin model development [18], applying literature values for all interaction parameters without further optimization. The implemented model processes are visualized in Figure S25. The parameters of the trimethoprim model are given in Table S2 and the parameters of the applied rifampicin model [18] are reproduced in Table S17.

The population predictions of trimethoprim plasma concentration-time profiles before and during rifampicin co-administration, compared to observed data, are shown in semilogarithmic (Figure S26) and linear plots (Figure S27). As no trimethoprim control group without co-administration of rifampicin was included in the only published study of the rifampicin-trimethoprim DDI, DDI AUC_{last} and C_{max} ratios were calculated as DDI AUC_{last} or C_{max} day 8 / DDI AUC_{last} or C_{max} day 1. The correlation of predicted and observed DDI AUC_{last} and C_{max} ratios is shown in Figure S28. Table S18 lists the corresponding predicted and observed DDI AUC_{last} and C_{max} ratios as well as GMFE values (GMFEs of 1.08 and 1.30, respectively).

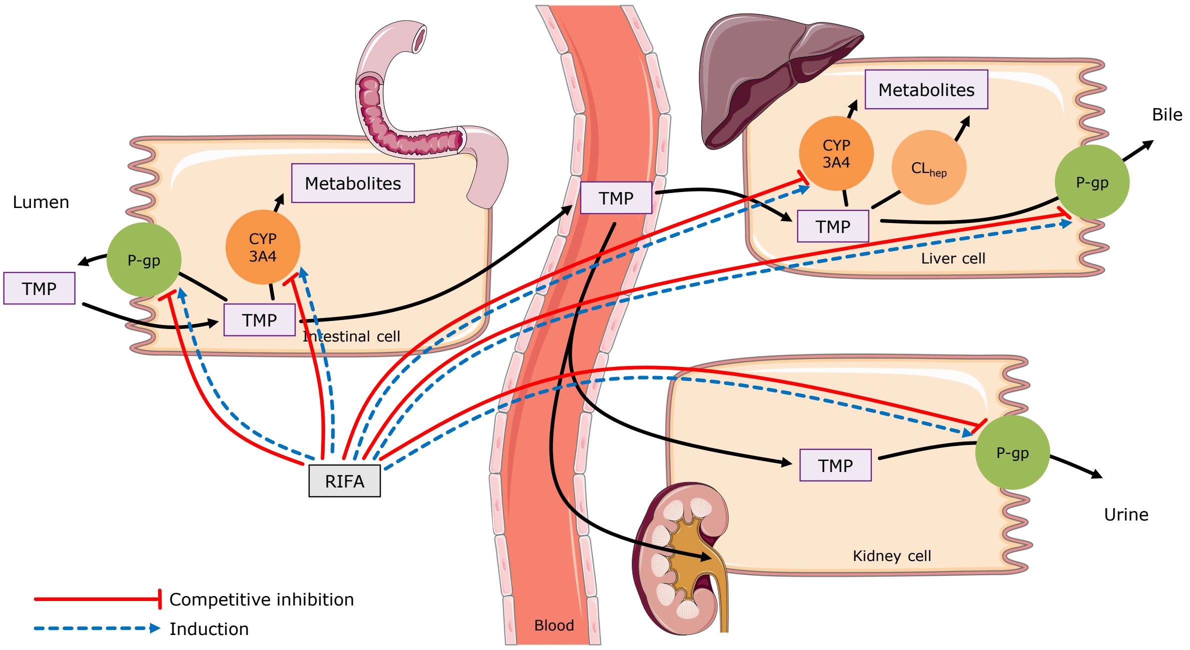

Figure S25: Rifampicin-trimethoprim DDI model processes. Drawings by Servier, licensed under CC BY 3.0. CL_{hep} hepatic metabolic clearance, *CYP* cytochrome P450, *P-gp* P-glycoprotein, *RIFA* rifampicin, *TMP* trimethoprim.

6.2 Clinical studies

Details on the clinical study investigating the rifampicin-trimethoprim DDI are given in Table S16.

Table S16: Clinical studies investigating the rifampicin-trimethoprim DDI

Perpetrator		Victim									
Route	Dose	Route	Dose	Dose gap	n	Females [%]	Age [years]	Weight [kg]	Height [cm]	BMI [kg/m^2]	Reference
Rifampicin		*Trimethoprim*									
po, tid, D1-8	300 mg	po, tid, D1-8	80 mg	0 h	6	33	18-40	-	-	-	Emmerson 1978 [100]

Values for age are given as range. - not given, *BMI* body mass index, *D* day of administration, *DDI* drug-drug interaction, *n* number of individuals studied, *po* oral, *tid* three times daily.

6.3 Rifampicin drug-dependent parameters

Table S17: Drug-dependent parameters of the rifampicin PBPK model [18]

Parameter	Value	Unit	Source	Literature	Reference	Description
Rifampicin						
MW	822.94	g/mol	Literature	822.94	[57]	Molecular weight
pKa_1 (acid)	1.70		Literature	1.70	[58]	Acid dissociation constant
pKa_2 (base)	7.90		Literature	7.90	[58]	Acid dissociation constant
Solubility (pH 7.5)	2.80	g/L	Literature	1.10 (pH 6.5), 1.40 (pH 6.8), 2.54 (pH 6.8), 2.80 (pH 7.5), 3.35 (pH 7.4)	[101–104]	Solubility
logP	2.50		Optimized	1.30, 2.70	[57, 101]	Lipophilicity
fu	17.00	%	Literature	11.00, 16.00, 17.00, 17.50	[101, 104–106]	Fraction unbound plasma
B/P ratio	0.89		Calculated	0.90	[107]	Blood/plasma concentration ratio
OATP1B1 K_M	1.50	µmol/L	Literature	1.50	[108]	Michaelis-Menten constant
OATP1B1 k_{cat}	7.80	1/min	Optimized	-	-	Transport rate constant
P-gp K_M	55.00	µmol/L	Literature	55.00	[109]	Michaelis-Menten constant
P-gp k_{cat}	0.61	1/min	Optimized	-	-	Transport rate constant
AADAC K_M	195.10	µmol/L	Literature	195.10	[110]	Michaelis-Menten constant
AADAC k_{cat}	9.87	1/min	Optimized	-	-	Catalytic rate constant
GFR fraction	1		Assumed	-	-	Fraction of filtered drug in the urine
EHC continuous fraction	1		Assumed	-	-	Fraction of bile continually released
Induction EC_{50}	0.34	µmol/L	Literature	0.34	[105, 106]	Conc. for half-maximal induction
E_{max} OATP1B1	0.38		Optimized	-	-	Maximum in vivo induction effect
E_{max} P-gp	2.50		Literature	2.50	[99]	Maximum in vivo induction effect
E_{max} AADAC	0.99		Optimized	-	-	Maximum in vivo induction effect
E_{max} CYP3A4	9.00		Literature	9.00	[105]	Maximum in vivo induction effect
OATP1B1 K_i	0.48	µmol/L	Literature	0.48	[111]	Conc. for 50% inhibition (competitive)
P-gp K_i	169.00	µmol/L	Literature	169.00	[112]	Conc. for 50% inhibition (competitive)
CYP3A4 K_i	18.50	µmol/L	Literature	18.50	[92]	Conc. for 50% inhibition (competitive)
Partition coefficients	Diverse		Calculated	R+R	[113, 114]	Cell to plasma partition coefficients
Cellular permeability	2.93E-05	cm/min	Calculated	PK-Sim	[98]	Permeability into the cellular space
Intestinal permeability	1.24E-05	cm/min	Optimized	3.84E-07	Calculated	Transcellular intestinal permeability
Formulation	Solution					Formulation used in predictions

AADAC arylacetamide deacetylase, *conc.* concentration, *CYP* cytochrome P450, *EHC* enterohepatic circulation, *GFR* glomerular filtration rate, *OATP* organic-anion-transporting polypeptide, *P-gp* P-glycoprotein, *PK-Sim* PK-Sim standard calculation method, *R+R* Rodgers and Rowland calculation method.

6.4 Profiles

6.4.1 Semilogarithmic plots - Plasma

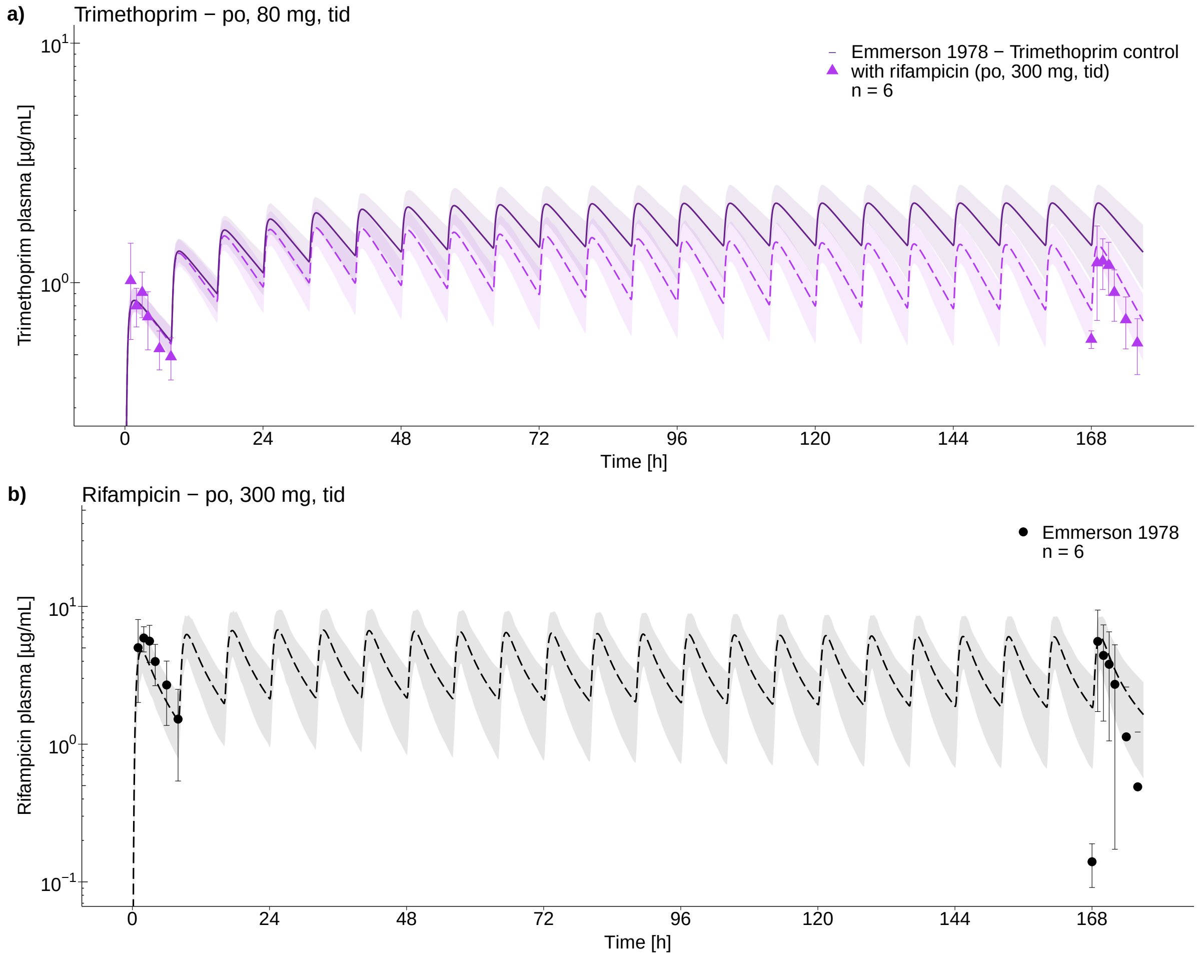

Figure S26: (**a**) Trimethoprim plasma concentration-time profiles alone and during rifampicin co-administration (semilogarithmic). (**b**) Rifampicin plasma concentration-time profile during the rifampicin-trimethoprim DDI (semilogarithmic). Observed data are shown as triangles (trimethoprim training dataset) or circles (rifampicin) ± standard deviation. Population simulation arithmetic means are shown as lines (solid line: trimethoprim alone; dashed line(s): trimethoprim during rifampicin co-administration or rifampicin during the DDI); the shaded areas represent the 68% population prediction intervals. Details on dosing regimens, study populations and literature references are listed in Table S16. Predicted and observed DDI AUC_{last} and C_{max} ratios are summarized in Table S18. *n* number of individuals studied, *po* oral, *tid* three times daily.

6.4.2 Linear plots - Plasma

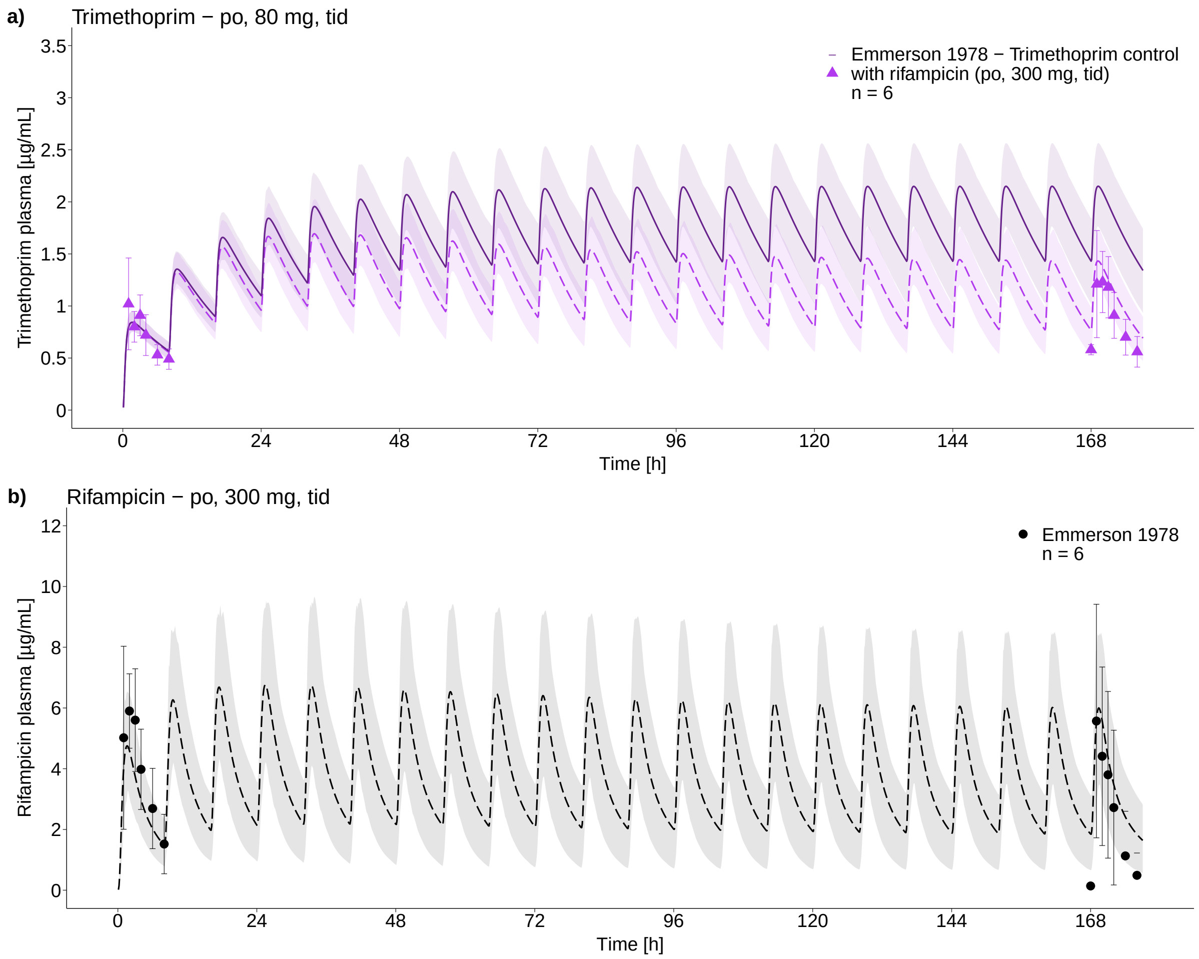

Figure S27: (**a**) Trimethoprim plasma concentration-time profiles alone and during rifampicin co-administration (linear). (**b**) Rifampicin plasma concentration-time profile during the rifampicin-trimethoprim DDI (linear). Observed data are shown as triangles (trimethoprim training dataset) or circles (rifampicin) ± standard deviation. Population simulation arithmetic means are shown as lines (solid line: trimethoprim alone; dashed line(s): trimethoprim during rifampicin co-administration or rifampicin during the DDI); the shaded areas represent the 68% population prediction intervals. Details on dosing regimens, study populations and literature references are listed in Table S16. Predicted and observed DDI AUC_{last} and C_{max} ratios are summarized in Table S18. *n* number of individuals studied, *po* oral, *tid* three times daily.

6.5 DDI model performance evaluation

6.5.1 DDI AUC_{last} and C_{max} ratio goodness-of-fit plots

a) DDI AUC_{last} ratio

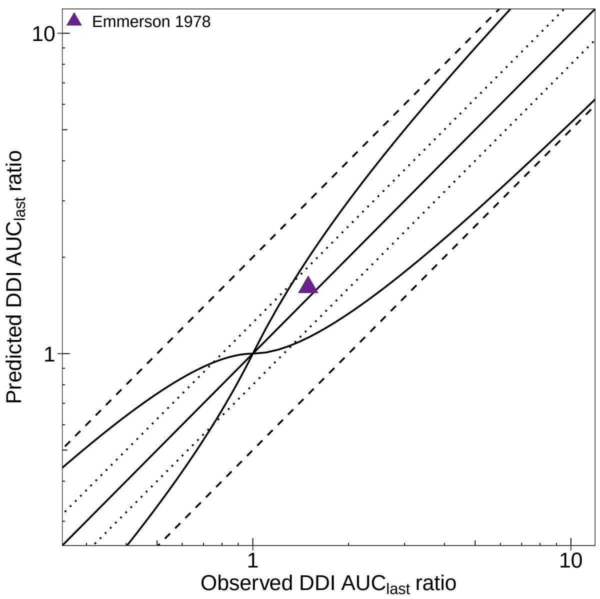

b) DDI C_{max} ratio

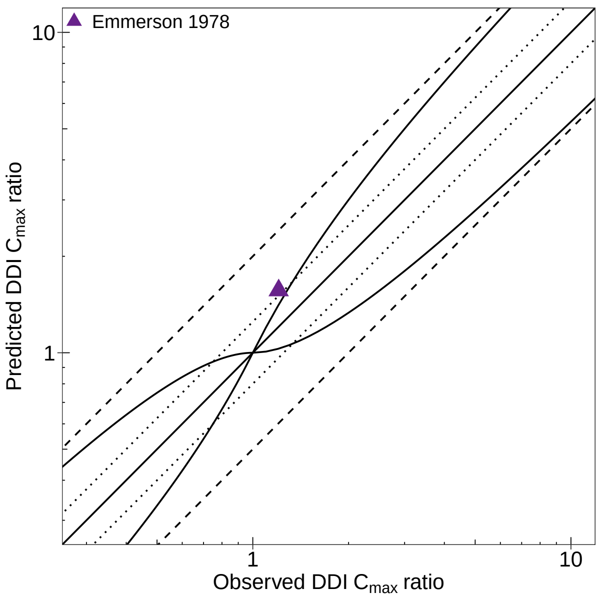

Figure S28: Comparison of predicted to the corresponding observed trimethoprim DDI (**a**) AUC_{last} and (**b**) C_{max} ratios of the rifampicin-trimethoprim DDI. The solid straight line marks the line of identity, dotted lines indicate 1.25-fold and dashed lines indicate 2-fold deviation. The curved lines show the prediction success limits suggested by Guest et al. [81]. Details on the study protocols and the predicted and observed DDI AUC_{last} and C_{max} ratios are given in Tables S16 and S18, respectively.

6.5.2 Predicted and observed DDI AUC_{last} and C_{max} ratios with GMFE values

Table S18: Predicted and observed rifampicin-trimethoprim DDI AUC_{last} and C_{max} ratios

Perpetrator		Victim			DDI AUC_{last} ratio [a]			DDI C_{max} ratio [a]			
Route	Dose	Route	Dose	t_{last}	Pred	Obs	Pred/Obs	Pred	Obs	Pred/Obs	Reference
Rifampicin		*Trimethoprim*									
po, tid, D1-8	300 mg	po, tid, D1-8	80 mg	8 h	1.61	1.49	1.08	1.57	1.21	1.30	Emmerson 1978 [100]
**GMFE**							**1.08**			**1.30**	
							**1/1 with GMFE ≤ 2**			**1/1 with GMFE ≤ 2**	

[a] DDI AUC_{last} and C_{max} ratios are calculated as DDI AUC_{last} or C_{max} day 8 / DDI AUC_{last} or C_{max} day 1, as no trimethoprim plasma concentrations without rifampicin co-administration were reported in this study. *AUC* area under the concentration-time curve, C_{max} peak plasma concentration, *D* day of administration, *DDI* drug-drug interaction, *GMFE* geometric mean fold error, *obs* observed, *po* oral, *pred* predicted, t_{last} time of the last concentration measurement, *tid* three times daily.

7 System-dependent parameters

Details on the expression of drug transporters and metabolic enzymes implemented to model the pharmacokinetics of trimethoprim, metformin, repaglinide, pioglitazone and rifampicin are summarized in Table S19.

Table S19: System-dependent parameters

	Reference concentration					Half-life	
Transporter/enzyme	Mean [µmol/L] [a]	GeoSD [b]	Expression profile [c]	Localization	Direction	Liver [h]	Intestine [h]
Transporters							
MATE1	0.13 [d] [115, 118]	1.53 [118]	Kidney only [119, 120]	Apical	Efflux	36	-
OATP1B1	1.00 [e] [116]	1.54 [117]	RT-PCR [121]	Basolateral	Influx	36	23
OATP1B3	1.00 [e] [116]	1.54 [117]	Array [122]	Basolateral	Influx		
OCT1	0.16 [f] [117, 123]	1.53 [123]	Array [122],<br>large intestinal mucosa → 0	Basolateral,<br>in enterocytes apical	Influx	36	23
OCT2	0.19 [d] [115, 118]	1.45 [118]	EST [124]	Basolateral	Influx	36	-
P-gp (efflux)	1.41 [18]	1.60 [117]	RT-PCR [121],<br>intestinal mucosa → factor 3.57 [18]	Apical	Efflux	36	23
PMAT	1.00 [e] [116]	1.40 [g]	RT-PCR [121],<br>large intestinal mucosa → 0	Basolateral,<br>in enterocytes apical	Influx	36	23
Enzymes							
AADAC	1.00 [e] [116]	1.40 [g]	RT-PCR [125]	Intracellular	-	36	23
CYP2C8	2.56 [126]	2.05 [12]	RT-PCR [127]	Intracellular	-	23	23
CYP2C8*1	1.28	2.05	RT-PCR [127]	Intracellular	-	23	23
CYP2C8*3	1.28	2.05	RT-PCR [127]	Intracellular	-	23	23
CYP3A4	4.32 [126]	1.18 liver [12],<br>1.46 intestine [12]	RT-PCR [127]	Intracellular	-	36 [128]	23 [129]
Processes							
Hepatic metabolic clearance	-	1.40 [g]	-	-	-	-	

[a] µmol/L in the tissue of highest expression, [b] geometric standard deviation of the reference concentration, [c] relative expression in the different organs (PK-Sim expression database profile), [d] calculated from transporter per mg membrane protein x 26.2 mg human kidney microsomal protein per g kidney [115], [e] if no information was available, the mean reference concentration was set to 1.0 µmol/L and the transport or catalytic rate constant (k_{cat}) was optimized according to [116], [f] calculated from transporter per mg membrane protein x 37.0 mg membrane protein per g liver [117], [g] if no information was available, a moderate variability of 35% CV was assumed (≙ 1.40 GeoSD). *AADAC* arylacetamide deacetylase, *Array* ArrayExpress measured expression profile, *CYP* cytochrome P450, *EST* expressed sequence tag measured expression profile, *MATE* multidrug and toxin extrusion protein, *OATP* organic-anion-transporting polypeptide, *OCT* organic cation transporter, *P-gp* P-glycoprotein, *PMAT*, plasma membrane monoamine transporter, *RT-PCR* reverse transcription-polymerase chain reaction measured expression profile.

List of Tables

S1 Clinical studies of trimethoprim 14
S2 Drug-dependent parameters of the final trimethoprim PBPK model 16
S3 MRD values of trimethoprim plasma (or whole blood) concentration predictions . 41
S4 Predicted and observed trimethoprim fractions excreted unchanged in urine 44
S5 Predicted and observed trimethoprim AUC_{last} and C_{max} values 46
S6 Parameters evaluated during trimethoprim sensitivity analysis 48

S7 Clinical studies investigating the trimethoprim-metformin DDI and DDGI 51
S8 Drug-dependent parameters of the metformin PBPK model [6] 52
S9 Predicted and observed trimethoprim-metformin DDI and DDGI AUC_{last} and C_{max} ratios 56

S10 Clinical studies investigating the trimethoprim-repaglinide DDI 58
S11 Drug-dependent parameters of the repaglinide PBPK model [17] 59
S12 Predicted and observed trimethoprim-repaglinide DDI AUC_{last} and C_{max} ratios . . 62

S13 Clinical studies investigating the trimethoprim-pioglitazone DDI and DDGI 64
S14 Drug-dependent parameters of the pioglitazone PBPK model [17] 65
S15 Predicted and observed trimethoprim-pioglitazone DDI and DDGI AUC_{last} and C_{max} ratios 69

S16 Clinical studies investigating the rifampicin-trimethoprim DDI 71
S17 Drug-dependent parameters of the rifampicin PBPK model [18] 72
S18 Predicted and observed rifampicin-trimethoprim DDI AUC_{last} and C_{max} ratios . . 76

S19 System-dependent parameters 77

List of Figures

S1 Trimethoprim DDI network. 9

S2 Comparison of trimethoprim administered alone or together with sulfamethoxazole as "cotrimoxazole" 12
S3 Schematic illustration of the trimethoprim ADME processes in the model 13
S4 Trimethoprim plasma concentration-time profiles (semilogarithmic) 17
S5 Trimethoprim plasma concentration-time profiles after "cotrimoxazole" administration (semilogarithmic) 20
S6 Trimethoprim plasma concentration-time profiles (linear) 26
S7 Trimethoprim plasma concentration-time profiles after "cotrimoxazole" administration (linear) 29
S8 Trimethoprim fraction excreted unchanged in urine profiles 35
S9 Trimethoprim fraction excreted unchanged in urine profiles after "cotrimoxazole" administration 37
S10 Trimethoprim predicted compared to observed plasma concentration values 40
S11 Trimethoprim predicted compared to observed fractions excreted unchanged in urine 43
S12 Trimethoprim predicted compared to observed AUC_{last} and C_{max} values 45
S13 Trimethoprim sensitivity analysis 49

S14 Trimethoprim-metformin DDI model processes 50
S15 Metformin plasma concentration-time profiles before and during trimethoprim DDI and DDGI (semilogarithmic) 53
S16 Metformin plasma concentration-time profiles before and during trimethoprim DDI and DDGI (linear) 54
S17 Metformin predicted compared to observed DDI and DDGI AUC_{last} and C_{max} ratios 55

S18 Trimethoprim-repaglinide DDI model processes 57
S19 Repaglinide plasma concentration-time profiles before and during trimethoprim DDI 60
S20 Repaglinide predicted compared to observed DDI AUC_{last} and C_{max} ratios 61

S21 Trimethoprim-pioglitazone DDI model processes 63
S22 Pioglitazone plasma concentration-time profiles before and during trimethoprim DDI and DDGI (semilogarithmic) 66
S23 Pioglitazone plasma concentration-time profiles before and during trimethoprim DDI and DDGI (linear) 67
S24 Pioglitazone predicted compared to observed DDI and DDGI AUC_{last} and C_{max} ratios 68

S25 Rifampicin-trimethoprim DDI model processes 70
S26 Trimethoprim and rifampicin plasma concentration-time profiles of the rifampicin DDI (semilogarithmic) 73
S27 Trimethoprim and rifampicin plasma concentration-time profiles of the rifampicin DDI (linear) 74
S28 Trimethoprim predicted compared to observed DDI AUC_{last} and C_{max} ratios . . . 75

Abbreviations

AADAC Arylacetamide deacetylase

ADME Absorption, distribution, metabolism and excretion

AUC Area under the concentration-time curve

AUC_{0-12} Area under the concentration-time curve from 0 to 12 h

AUC_{last} AUC values calculated from the time of drug administration to the time of the last concentration measurement

$AUC_{0-\infty}$ Area under the concentration-time curve from 0 extrapolated to infinity

bid Twice daily

BMI Body mass index

B/P ratio Blood/plasma concentration ratio

caps Capsule

CHO Chinese hamster ovary cell line

CL_{hep} Hepatic metabolic clearance

C_{max} Maximum plasma concentration

CYP Cytochrome P450

D Day of administration

DDI Drug-drug interaction

DDGI Drug-drug-gene interaction

DGI Drug-gene interaction

EC50 Concentration for half maximal induction in vivo

EHC Enterohepatic circulation

E_{max} Maximal induction effect in vivo

EST Expressed sequence tag

fe Fration excreted unchanged

fu Fraction unbound in plasma

fu_{inc} Fraction unbound in the incubation

GFR Glomerular filtration rate

GMFE Geometric mean fold error

HEK 293 Human embryonic kidney 293 cell line

$\mathbf{IC_{50}}$	Half maximal inhibitory concentration
**ICRP**	International Commission on Radiological Protection
**iv**	Intravenous
$\mathbf{k_{cat}}$	Transport or catalytic rate constant
$\mathbf{k_{deg}}$	Degradation rate constant
$\mathbf{K_i}$	Dissociation constant of the inhibitor-transporter/ -enzyme complex
$\mathbf{K_M}$	Michaelis-Menten constant
$\mathbf{K_{M,app}}$	Michaelis-Menten constant in the presence of inhibitor
**logP**	Lipophilicity
**MATE**	Multidrug and toxin extrusion protein
**MRD**	Mean relative deviation
**MW**	Molecular weight
**n**	Number of individuals studied
**NHANES**	National Health and Nutrition Examination Survey
**OATP**	Organic-anion-transporting polypeptide
**obs**	Observed
**OCT**	Organic cation transporter
**P-gp**	P-glycoprotein
**PBPK**	Physiologically-based pharmacokinetic
**pKa**	Acid dissociation constant
**PMAT**	Plasma membrane monoamine transporter
**po**	Oral
**pred**	Predicted
**PXR**	Pregnane X receptor
**qid**	Four times daily
$\mathbf{R_{syn}}$	Rate of transporter or enzyme synthesis
$\mathbf{R_{syn,app}}$	Rate of transporter or enzyme synthesis in the presence of inducer
**RT-PCR**	Reverse transcription-polymerase chain reaction
**sd**	Single dose
**SLC**	Solute carrier family member

**SLCO**	Solute carrier organic antion transporter family member
**SNP**	Single nucleotide polymorphism
**susp**	Oral solution
**tab**	Tablet
**tid**	Three times daily
$\mathbf{t_{last}}$	Time of the last concentration measurement
$\mathbf{t_{max}}$	Time to maximum plasma concentration
**v**	Reaction velocity
$\mathbf{v_{max}}$	Maximum reaction velocity
**WB**	Whole blood

References

[1] O. Zolk, T. F. Solbach, H. König, and M. F. Fromm. Functional characterization of the human organic cation transporter 2 variant p.270Ala>Ser. *Drug Metabolism and Disposition*, 37(6): 1312–8, 2009.

[2] B. Grün, M. K. Kiessling, J. Burhenne, K. D. Riedel, J. Weiss, G. Rauch, W. E. Haefeli, and D. Czock. Trimethoprim-metformin interaction and its genetic modulation by OCT2 and MATE1 transporters. *British Journal of Clinical Pharmacology*, 76(5):787–96, 2013.

[3] Z. J. Wang, O. Q. P. Yin, B. Tomlinson, and M. S. S. Chow. OCT2 polymorphisms and in-vivo renal functional consequence: studies with metformin and cimetidine. *Pharmacogenetics and Genomics*, 18(7):637–45, 2008.

[4] Y. Chen, S. Li, C. Brown, S. Cheatham, R. A. Castro, M. K. Leabman, T. J. Urban, L. Chen, S. W. Yee, J. H. Choi, Y. Huang, C. M. Brett, E. G. Burchard, and K. M. Giacomini. Effect of genetic variation in the organic cation transporter 2 on the renal elimination of metformin. *Pharmacogenetics and Genomics*, 19(7):497–504, 2009.

[5] M. M. H. Christensen, R. S. Pedersen, T. B. Stage, C. Brasch-Andersen, F. Nielsen, P. Damkier, H. Beck-Nielsen, and K. Brøsen. A gene-gene interaction between polymorphisms in the OCT2 and MATE1 genes influences the renal clearance of metformin. *Pharmacogenetics and Genomics*, 23(10):526–34, 2013.

[6] N. Hanke, D. Türk, D. Selzer, N. Ishiguro, T. Ebner, S. Wiebe, F. Müller, P. Stopfer, V. Nock, and T. Lehr. A comprehensive whole-body physiologically based pharmacokinetic drug-drug-gene interaction model of metformin and cimetidine in healthy adults and renally impaired individuals. *Clinical Pharmacokinetics*, 2020.

[7] D. Dai, D. C. Zeldin, J. A. Blaisdell, B. Chanas, S. J. Coulter, B. I. Ghanayem, and J. A. Goldstein. Polymorphisms in human CYP2C8 decrease metabolism of the anticancer drug paclitaxel and arachidonic acid. *Pharmacogenetics*, 11(7):597–607, 2001.

[8] R. A. Totah and A. E. Rettie. Cytochrome P450 2C8: substrates, inhibitors, pharmacogenetics, and clinical relevance. *Clinical Pharmacology and Therapeutics*, 77(5):341–52, 2005.

[9] C. L. Aquilante, L. A. Kosmiski, D. W. A. Bourne, L. R. Bushman, E. B. Daily, K. P. Hammond, C. W. Hopley, R. S. Kadam, A. T. Kanack, U. B. Kompella, M. Le, J. A. Predhomme, J. E. Rower, and M. S. Sidhom. Impact of the CYP2C8 *3 polymorphism on the drug-drug interaction between gemfibrozil and pioglitazone. *British Journal of Clinical Pharmacology*, 75 (1):217–26, 2013.

[10] A. Tornio, M. Niemi, P. J. Neuvonen, and J. T. Backman. Trimethoprim and the CYP2C8*3 allele have opposite effects on the pharmacokinetics of pioglitazone. *Drug Metabolism and Disposition*, 36(1):73–80, 2008.

[11] E. Muschler, J. Lal, A. Jetter, A. Rattay, U. Zanger, G. Zadoyan, U. Fuhr, and J. Kirchheiner. The role of human CYP2C8 and CYP2C9 variants in pioglitazone metabolism in vitro. *Basic & Clinical Pharmacology & Toxicology*, 105(6):374–9, 2009.

[12] Open Systems Pharmacology Suite Community. PK-Sim® Ontogeny Database Documentation, Version 7.3, 2018. URL `https://github.com/Open-Systems-Pharmacology/OSPSuite.Documentation/blob/master/PK-Sim%20Ontogeny%20Database%20Version%207.3.pdf`. (accessed on Aug 24, 2020).

[13] J. Valentin. Basic anatomical and physiological data for use in radiological protection: reference values: ICRP Publication 89. *Annals of the ICRP*, 32(3):1–277, 2002.

[14] National Center for Health Statistics. Third National Health and Nutrition Examination Survey (NHANES III). *Tech. rep., Hyattsville, MD 20782*, 1997.

[15] G. Tanaka and H. Kawamura. Anatomical and physiological characteristics for Asian reference man: male and female of different ages: Tanaka model., 1996.

[16] Open Systems Pharmacology Suite Community. Open Systems Pharmacology Suite Manual, Version 7.4, 2018. URL `https://github.com/Open-Systems-Pharmacology/OSPSuite.Documentation/blob/master/OpenSystemsPharmacologySuite.pdf`. (accessed on Aug 24, 2020).

[17] D. Türk, N. Hanke, S. Wolf, S. Frechen, T. Eissing, T. Wendl, M. Schwab, and T. Lehr. Physiologically based pharmacokinetic models for prediction of complex CYP2C8 and OATP1B1 (SLCO1B1) drug-drug-gene interactions: a modeling network of gemfibrozil, repaglinide, pioglitazone, rifampicin, clarithromycin and itraconazole. *Clinical Pharmacokinetics*, 58(12): 1595–1607, 2019.

[18] N. Hanke, S. Frechen, D. Moj, H. Britz, T. Eissing, T. Wendl, and T. Lehr. PBPK models for CYP3A4 and P-gp DDI prediction: a modeling network of rifampicin, itraconazole, clarithromycin, midazolam, alfentanil, and digoxin. *CPT: pharmacometrics & systems pharmacology*, 7(10):647–59, 2018.

[19] T. P. Van Boeckel, S. Gandra, A. Ashok, Q. Caudron, B. T. Grenfell, S. A. Levin, and R. Laxminarayan. Global antibiotic consumption 2000 to 2010: an analysis of national pharmaceutical sales data. *The Lancet. Infectious Diseases*, 14(8):742–50, 2014.

[20] U.S. Food and Drug Administration. Drug development and drug interactions: table of substrates, inhibitors and inducers, 2017. URL `https://www.fda.gov/drugs/drug-interactions-labeling/drug-development-and-drug-interactions-table-substrates-inhibitors-and-inducers`. (accessed on Aug 24, 2020).

[21] R. Elsby, S. Chidlaw, S. Outteridge, S. Pickering, A. Radcliffe, R. Sullivan, H. Jones, and P. Butler. Mechanistic in vitro studies confirm that inhibition of the renal apical efflux transporter multidrug and toxin extrusion (MATE) 1, and not altered absorption, underlies the increased metformin exposure observed in clinical interactions with cimetidine, trimethoprim or pyrimethamine. *Pharmacology Research & Perspectives*, 5(5):1–13, 2017.

[22] F. Müller, C. A. Pontones, B. Renner, M. Mieth, E. Hoier, D. Auge, R. Maas, O. Zolk, and M. F. Fromm. N(1)-methylnicotinamide as an endogenous probe for drug interactions by renal cation transporters: studies on the metformin-trimethoprim interaction. *European Journal of Clinical Pharmacology*, 71(1):85–94, 2015.

[23] H. Nolte and H. Büttner. Pharmacokinetics of trimethoprim and its combination with sulfamethoxazole in man after single and chronic oral administration. *Chemotherapy*, 18(5):274–84, 1973.

[24] S. A. Kaplan, R. E. Weinfeld, C. W. Abruzzo, K. McFaden, M. Lewis Jack, and L. Weissman. Pharmacokinetic profile of trimethoprim-sulfamethoxazole in man. *Journal of Infectious Diseases*, 128(Supplement 3):S547–55, 1973.

[25] R. E. Weinfeld and T. C. Macasieb. Determination of trimethoprim in biological fluids by high-performance liquid chromatography. *Journal of Chromatography B*, 164(1):73–84, 1979.

[26] R. L. Guptat, R. Kumar, and A. K. Singla. Enhanced dissolution and absorption of trimethoprim from coprecipitates with polyethylene glycols and polyvinylpyrrolidone. *Drug Development and Industrial Pharmacy*, 17(3):463–68, 1991.

[27] Hoffmann-La Roche Inc. BACTRIM™ sulfamethoxazole and trimethoprim DS (double strength) tablets and tablets USP. 2013.

[28] Ratiopharm GmbH. Fachinformation Cotrim-ratiopharm® 480 mg Tabletten, Cotrim forte-ratiopharm® 960 mg Tabletten (Bioavailability Study 1987 and 1991), 2017.

[29] M. C. Bach, O. Gold, and M. Finland. Absorption and urinary execretion of trimethoprim, sulfamethoxazole, and trimethoprim-sulfamethoxazole: results with single doses in normal young adults and preliminary observations during therapy with trimethoprim-sulfamethoxazole. *The Journal of Infectious Diseases*, 128(Suppl):584–99, 1973.

[30] K. Hoppu, J. Tuomisto, O. Koskimies, and O. Simell. Food and guar decrease absorption of trimethoprim. *European Journal of Clinical Pharmacology*, 32(4):427–9, 1987.

[31] A. Klimowicz, A. Nowak, and M. Kadyków. Plasma and skin blister fluid concentrations of trimethoprim following its oral administration. *European Journal of Clinical Pharmacology*, 34 (4):377–80, 1988.

[32] M. Niemi, J. T. Backman, and P. J. Neuvonen. Effects of trimethoprim and rifampin on the pharmacokinetics of the cytochrome P450 2C8 substrate rosiglitazone. *Clinical Pharmacology and Therapeutics*, 76(3):239–49, 2004.

[33] R. M. Hutabarat, J. D. Unadkat, C. Sahajwalla, S. McNamara, B. Ramsey, and A. L. Smith. Disposition of drugs in cystic fibrosis. I. Sulfamethoxazole and trimethoprim. *Clinical Pharmacology and Therapeutics*, 49(4):402–9, 1991.

[34] P. T. Männistö, R. Mäntylä, J. Mattila, S. Nykänen, and U. Lamminsivu. Comparison of pharmacokinetics of sulphadiazine and sulphamethoxazole after intravenous infusion. *The Journal of Antimicrobial Chemotherapy*, 9(6):461–70, 1982.

[35] J. Spicehandler, A. A. Pollock, M. S. Simberkoff, and J. J. Rahal. Intravenous pharmacokinetics and in vitro bactericidal activity of trimethoprim-sulfamethoxazole. *Reviews of Infectious Diseases*, 4(2):562–5, 1982.

[36] Ratiopharm GmbH. Fachinformation Cotrim K-ratiopharm® 240 mg / 5 ml Saft, Cotrim E-ratiopharm® 480 mg / 5 ml Saft (Bioavailability Study 1988), 2017.

[37] MEDA Pharma GmbH & Co KG. Fachinformation Cotrim-Diolan® Suspension zum Einnehmen, 2013.

[38] D. V. DeAngelis, J. L. Woolley, and C. W. Sigel. High-performance liquid chromatographic assay for the simultaneous measurement of trimethoprim and sulfamethoxazole in plasma or urine. *Therapeutic Drug Monitoring*, 12(4):382–92, 1990.

[39] D.C.G. Bedor, T.M. Gonçalves, M.L.L. Ferreira, C.E.M. de Sousa, A.L. Menezes, E.J. Oliveira, and D.P. de Santana. Simultaneous determination of sulfamethoxazole and trimethoprim in biological fluids for high-throughput analysis: Comparison of HPLC with ultraviolet and tandem mass spectrometric detection. *Journal of Chromatography B*, 863(1):46–54, 2008.

[40] H. Amini and A. Ahmadiani. Rapid and simultaneous determination of sulfamethoxazole and trimethoprim in human plasma by high-performance liquid chromatography. *Journal of Pharmaceutical and Biomedical Analysis*, 43(3):1146–50, 2007.

[41] F. J. Flores-Murrieta, G. Castañeda-Hernández, J. C. Menéndez, F. Chávez, J. E. Herrera, and E. Hong. Pharmacokinetics of sulfamethoxazole and trimethoprim in Mexicans: bioequivalence of two oral formulations (URO-TS D® and Bactrim F®). *Biopharmaceutics & Drug Disposition*, 11(9):765–72, 1990.

[42] R. Gochin, I. Kanfer, and J. M. Haigh. Simultaneous determination of trimethoprim, sulphamethoxazole and N4-acetylsulphamethoxazole in serum and urine by high-performance liquid chromatography. *Journal of Chromatography*, 223(1):139–45, 1981.

[43] H. N. Mistri, A. G. Jangid, A. Pudage, A. Shah, and P. S. Shrivastav. Simultaneous determination of sulfamethoxazole and trimethoprim in microgram quantities from low plasma volume by liquid chromatography–tandem mass spectrometry. *Microchemical Journal*, 94(2):130–8, 2010.

[44] B. Örtengren, L. Magni, and T. Bergan. Development of sulphonamide-trimethoprim combinations for urinary tract infections. Part 3: Pharmacokinetic characterization of sulphadiazine and sulphamethoxazole given with trimethoprim. *Infection*, 7(Suppl 4):S371–81, 1979.

[45] O. Varoquaux, D. Lajoie, C. Gobert, P. Cordonnier, C. Ducreuzet, M. Pays, and C. Advenier. Pharmacokinetics of the trimethoprim-sulphamethoxazole combination in the elderly. *British Journal of Clinical Pharmacology*, 20(6):575–81, 1985.

[46] I. D. Watson, H. N. Cohen, M. J. Stewart, S. J. McIntosh, A. Shenkin, and J. A. Thomson. Comparative pharmacokinetics of co-trifamole and co-trimoxazole to 'steady state' in normal subjects. *British Journal of Clinical Pharmacology*, 14(3):437–43, 1982.

[47] P. G. Welling, W. A. Craig, G. L. Amidon, and C. M. Kunin. Pharmacokinetics of trimethoprim and sulfamethoxazole in normal subjects and in patients with renal failure. *The Journal of Infectious Diseases*, 128(Suppl):556–66, 1973.

[48] J. N. Bruun, N. Ostby, J. E. Bredesen, P. Kierulf, and P. K. Lunde. Sulfonamide and trimethoprim concentrations in human serum and skin blister fluid. *Antimicrobial Agents and Chemotherapy*, 19(1):82–5, 1981.

[49] A. Królicki, A. Klimowicz, S. Bielecka-Grzela, A. Nowak, and R. Maleszka. Penetration of cotrimoxazole components into skin after a single oral dose. Theoretical versus experimental approach. *Polish Journal of Pharmacology*, 56(2):257–63, 2004.

[50] F. B. Eatman, A. C. Maggio, R. Pocelinko, H. G. Boxenbaum, K. A. Geitner, W. Glover, T. Macasieb, A. Holazo, R. E. Weinfeld, and S. A. Kaplan. Blood and salivary concentrations of sulfamethoxazole and trimethoprim in man. *Journal of Pharmacokinetics and Biopharmaceutics*, 5(6):615–24, 1977.

[51] T. T. Yoshikawa and L. B. Guze. Concentrations of trimethoprim-sulfamethoxazole in blood after a single, large oral Dose. *Antimicrobial Agents and Chemotherapy*, 10(3):462–3, 1976.

[52] R. J. Fass, R. B. Prior, and R. L. Perkins. Pharmacokinetics and tolerance of a single twelve-tablet dose of trimethoprim (960 mg)-sulfamethoxazole (4,800 mg). *Antimicrobial Agents and Chemotherapy*, 12(1):102–6, 1977.

[53] D. S. Reeves and P. J. Wilkinson. The pharmacokinetics of trimethoprim and trimethoprim/sulphonamide combinations, including penetration into body tissues. *Infection*, 7(Suppl 4):S330–41, 1979.

[54] R. C. Stevens, S. C. Laizure, P. L. Sanders, and D. S. Stein. Multiple-dose pharmacokinetics of 12 milligrams of trimethoprim and 60 milligrams of sulfamethoxazole per kilogram of body weight per day in healthy volunteers. *Antimicrobial Agents and Chemotherapy*, 37(3):448–52, 1993.

[55] R C Stevens, S. C. Laizure, C. L. Williams, and D. S. Stein. Pharmacokinetics and adverse effects of 20-mg/kg/day trimethoprim and 100-mg/kg/day sulfamethoxazole in healthy adult subjects. *Antimicrobial Agents and Chemotherapy*, 35(9):1884–90, 1991.

[56] Y. Cheng and W. H. Prusoff. Relationship between the inhibition constant (K1) and the concentration of inhibitor which causes 50 per cent inhibition (I50) of an enzymatic reaction. *Biochemical Pharmacology*, 22(23):3099–3108, 1973.

[57] D. S. Wishart, C. Knox, A. C. Guo, S. Shrivastava, M. Hassanali, P. Stothard, Z. Chang, and J. Woolsey. DrugBank: a comprehensive resource for in silico drug discovery and exploration. *Nucleic Acids Research*, 34(Database issue):D668–72, 2006.

[58] M. J. O'Neil, P. E. Heckelman, C. B. Koch, K. J. Roman, C. M. Kenny, and M. R. D'Arecca. *The Merck Index: An Encyclopedia of Chemicals, Drugs and Biologicals*. 14th edition edition, 2006.

[59] S. Kim, J. Chen, T. Cheng, A. Gindulyte, J. He, S. He, Q. Li, B. A. Shoemaker, P. A. Thiessen, B. Yu, L. Zaslavsky, J. Zhang, and E. E. Bolton. PubChem 2019 update: improved access to chemical data. *Nucleic Acids Research*, 47(D1):D1102–9, 2019.

[60] M. Fresta, P. M. Furneri, E. Mezzasalma, V. M. Nicolosi, and G. Puglisi. Correlation of trimethoprim and brodimoprim physicochemical and lipid membrane interaction properties with their accumulation in human neutrophils. *Antimicrobial Agents and Chemotherapy*, 40 (12):2865–73, 1996.

[61] N. A. Kasim, M. Whitehouse, C. Ramachandran, M. Bermejo, H. Lennernäs, A. Hussain, H. E. Junginger, S. A. Stavchansky, K. K. Midha, V. P. Shah, and G. L. Amidon. Molecular properties of WHO essential drugs and provisional biopharmaceutical classification. *Molecular Pharmaceutics*, 1(1):85–96, 2004.

[62] A. Wijkström and D. Westerlund. Plasma protein binding of sulphadiazine, sulphamethoxazole and trimethoprim determined by ultrafiltration. *Journal of Pharmaceutical and Biomedical Analysis*, 1(3):293–9, 1983.

[63] E. Singlas, J. N. Colin, J. Rottembourg, J. P. Meessen, A. de Martin, M. Legrain, and P. Simon. Pharmacokinetics of sulfamethoxazole–trimethoprim combination during chronic peritoneal dialysis: effect of peritonitis. *European Journal of Clinical Pharmacology*, 21(5):409–15, 1982.

[64] D. Gonzalez, S. Schmidt, and H. Derendorf. Importance of relating efficacy measures to unbound drug concentrations for anti-infective agents. *Clinical Microbiology Reviews*, 26(2):274–88, 2013.

[65] E. I. Lepist, X. Zhang, J. Hao, J. Huang, A. Kosaka, G. Birkus, B. P. Murray, R. Bannister, T. Cihlar, Y. Huang, and A. S. Ray. Contribution of the organic anion transporter OAT2 to

the renal active tubular secretion of creatinine and mechanism for serum creatinine elevations caused by cobicistat. *Kidney International*, 86(2):350–7, 2014.

[66] F. Müller, J. König, H. Glaeser, I. Schmidt, O. Zolk, M. F. Fromm, and R. Maas. Molecular Mechanism of Renal Tubular Secretion of the Antimalarial Drug Chloroquine. *Antimicrobial Agents and Chemotherapy*, 55(7):3091–8, 2011.

[67] C. Lechner, N. Ishiguro, A. Fukuhara, H. Shimizu, N. Ohtsu, M. Takatani, K. Nishiyama, I. Washio, N. Yamamura, and H. Kusuhara. Impact of experimental conditions on the evaluation of interactions between multidrug and toxin extrusion proteins and candidate drugs. *Drug Metabolism and Disposition*, 44(8):1381–9, 2016.

[68] F. Müller, J. König, E. Hoier, K. Mandery, and M. F. Fromm. Role of organic cation transporter OCT2 and multidrug and toxin extrusion proteins MATE1 and MATE2-K for transport and drug interactions of the antiviral lamivudine. *Biochemical Pharmacology*, 86(6):808–15, 2013.

[69] X. Chu, K. Bleasby, G. H. Chan, I. Nunes, and R. Evers. The complexities of interpreting reversible elevated serum creatinine levels in drug development: does a correlation with inhibition of renal transporters exist? *Drug Metabolism and Disposition*, 44(9):1498–1509, 2016.

[70] J. Dinger, M. R. Meyer, and H. H. Maurer. Development of an in vitro cytochrome P450 cocktail inhibition assay for assessing the inhibition risk of drugs of abuse. *Toxicology Letters*, 230(1):28–35, 2014.

[71] L. M. Berezhkovskiy. Volume of distribution at steady state for a linear pharmacokinetic system with peripheral elimination. *Journal of Pharmaceutical Sciences*, 93(6):1628–40, 2004.

[72] L.C. Block, L. O. Schemling, A. G. Couto, S. C. Mourão, and T. M. B. Bresolin. Pharmaceutical equivalence of metformin tablets with various binders. *Revista de Ciências Farmacêuticas Básica e Aplicada*, 29(1):29–35, 2008.

[73] D. Desai, B. Wong, Y. Huang, Q. Ye, D. Tang, H. Guo, M. Huang, and P. Timmins. Surfactant-mediated dissolution of metformin hydrochloride tablets: wetting effects versus ion pairs diffusivity. *Journal of Pharmaceutical Sciences*, 103(3):920–6, 2014.

[74] G. G. Graham, J. Punt, M. Arora, R. O. Day, M. P. Doogue, J. K. Duong, T. J. Furlong, J. R. Greenfield, L. C. Greenup, C. M. Kirkpatrick, J. E. Ray, P. Timmins, and K. M. Williams. Clinical pharmacokinetics of metformin. *Clinical Pharmacokinetics*, 50(2):81–98, 2011.

[75] G. T. Tucker, C. Casey, P. J. Phillips, H. Connor, J. D. Ward, and H. F. Woods. Metformin kinetics in healthy subjects and in patients with diabetes mellitus. *British Journal of Clinical Pharmacology*, 12(2):235–46, 1981.

[76] P. J. Pentikäinen, P. J. Neuvonen, and A. Penttilä. Pharmacokinetics of metformin after intravenous and oral administration to man. *European Journal of Clinical Pharmacology*, 16 (3):195–202, 1979.

[77] C. R. Sirtori, G. Franceschini, M. Galli-Kienle, G. Cighetti, G. Galli, A. Bondioli, and F. Conti. Disposition of metformin (N,N-dimethylbiguanide) in man. *Clinical Pharmacology and Therapeutics*, 24(6):683–93, 1978.

[78] J. Yin, H. Duan, and J. Wang. Impact of Substrate-Dependent Inhibition on Renal Organic Cation Transporters hOCT2 and hMATE1/2-K-Mediated Drug Transport and Intracellular Accumulation. *The Journal of Pharmacology and Experimental Therapeutics*, 359(3):401–10, 2016.

[79] Mingyan Zhou, L. Xia, and J. Wang. Metformin transport by a newly cloned proton-stimulated organic cation transporter (plasma membrane monoamine transporter) expressed in human intestine. *Drug Metabolism and Disposition*, 35(10):1956–62, 2007.

[80] S. Willmann, J. Lippert, and W. Schmitt. From physicochemistry to absorption and distribution: predictive mechanistic modelling and computational tools. *Expert Opinion on Drug Metabolism & Toxicology*, 1(1):159–68, 2005.

[81] E. J. Guest, L. Aarons, J. B. Houston, A. Rostami-Hodjegan, and A. Galetin. Critique of the two-fold measure of prediction success for ratios: application for the assessment of drug-drug interactions. *Drug Metabolism and Disposition*, 39(2):170–3, 2011.

[82] R. P. Austin, P. Barton, S. L. Cockroft, M. C. Wenlock, and R. J. Riley. The influence of nonspecific microsomal binding on apparent intrinsic clearance, and its prediction from physicochemical properties. *Drug Metabolism and Disposition*, 30(12):1497–1503, 2002.

[83] M. Niemi, L. I. Kajosaari, M. Neuvonen, J. T. Backman, and P. J. Neuvonen. The CYP2C8 inhibitor trimethoprim increases the plasma concentrations of repaglinide in healthy subjects. *British Journal of Clinical Pharmacology*, 57(4):441–7, 2004.

[84] Z. Zhu, T. Yang, Y. Zhao, N. Gao, D. Leng, and P. Ding. A simple method to improve the dissolution of repaglinide and exploration of its mechanism. *Asian Journal of Pharmaceutical Sciences*, 9(4):218–25, 2014.

[85] M. V. S. Varma, Y. Lai, E. Kimoto, T. C. Goosen, A. F. El-Kattan, and V. Kumar. Mechanistic modeling to predict the transporter- and enzyme-mediated drug-drug interactions of repaglinide. *Pharmaceutical Research*, 30(4):1188–99, 2013.

[86] Z. Mandić and V. Gabelica. Ionization, lipophilicity and solubility properties of repaglinide. *Journal of Pharmaceutical and Biomedical Analysis*, 41(3):866–71, 2006.

[87] N. Seedher and M. Kanojia. Co-solvent solubilization of some poorly-soluble antidiabetic drugs. *Pharmaceutical Development and Technology*, 14(2):185–92, 2009.

[88] T. C. Marbury, J. L. Ruckle, V. Hatorp, M. P. Andersen, K. K. Nielsen, W. C. Huang, and P. Strange. Pharmacokinetics of repaglinide in subjects with renal impairment. *Clinical Pharmacology and Therapeutics*, 67(1):7–15, 2000.

[89] V. Hatorp, K. H. Walther, M. S. Christensen, and G. Haug-Pihale. Single-dose pharmacokinetics of repaglinide in subjects with chronic liver disease. *Journal of Clinical Pharmacology*, 40(2):142–52, 2000.

[90] A. Plum, L. K. Müller, and J. A. Jansen. The effects of selected drugs on the in vitro protein binding of repaglinide in human plasma. *Methods and Findings in Experimental and Clinical Pharmacology*, 22(3):139–43, 2000.

[91] K. Ménochet, K. E. Kenworthy, J. B. Houston, and A. Galetin. Use of mechanistic modeling to assess interindividual variability and interspecies differences in active uptake in human and rat hepatocytes. *Drug Metabolism and Disposition*, 40(9):1744–56, 2012.

[92] L. I. Kajosaari, J. Laitila, P. J. Neuvonen, and J. T. Backman. Metabolism of repaglinide by CYP2C8 and CYP3A4 in vitro: effect of fibrates and rifampicin. *Basic & Clinical Pharmacology & Toxicology*, 97(4):249–56, 2005.

[93] W Schmitt. General approach for the calculation of tissue to plasma partition coefficients. *Toxicology in Vitro*, 22(2):457–67, 2008.

[94] S. K. Saha, A.K. A. Chowdhury, S. C. Bachar, S. C. Das, R. H. Kuddus, and M. A. Uddin. Comparative in vitro-in vivo correlation analysis with pioglitazone tablets. *Asian Pacific Journal of Tropical Disease*, 3(6):487–91, 2013.

[95] Takeda Canada Inc. ACTOS® (pioglitazone hydrochloride) 15, 30, 45 mg Tablets USP - Product Monograph, 2018.

[96] Y. Tsume, G. L. Amidon, and T. Susumu. Dissolution Effect of Gastric and Intestinal pH fora BCS class II drug, Pioglitazone: New in vitro Dissolution System to Predict in vivo Dissolution. *Journal of Bioequivalence & Bioavailability*, 5(6):224–7, 2013.

[97] C. Giaginis, S. Theocharis, and A. Tsantili-Kakoulidou. Investigation of the lipophilic behaviour of some thiazolidinediones. Relationships with PPAR-γ activity. *Journal of Chromatography. B*, 857(2):181–7, 2007.

[98] R. Kawai, M. Lemaire, J. L. Steimer, A. Bruelisauer, W. Niederberger, and M. Rowland. Physiologically based pharmacokinetic study on a cyclosporin derivative, SDZ IMM 125. *Journal of Pharmacokinetics and Biopharmaceutics*, 22(5):327–65, 1994.

[99] B. Greiner, M. Eichelbaum, P. Fritz, H. P. Kreichgauer, O. von Richter, J. Zundler, and H. K. Kroemer. The role of intestinal P-glycoprotein in the interaction of digoxin and rifampin. *The Journal of Clinical Investigation*, 104(2):147–53, 1999.

[100] A. M. Emmerson, R. N. Grüneberg, and E. S. Johnson. The phamacokinetics in man of a combination of rifampicin and trimethoprim. *Journal of Antimicrobial Chemotherapy*, 4(6): 523–31, 1978.

[101] G. Baneyx, N. Parrott, C. Meille, A. Iliadis, and T. Lavé. Physiologically based pharmacokinetic modeling of CYP3A4 induction by rifampicin in human: influence of time between substrate and inducer administration. *European Journal of Pharmaceutical Sciences*, 56:1–15, 2014.

[102] R. Panchagnula, I. Gulati, M. V. S. Varma, and Y. Ashok Raj. Dissolution Methodology for Evaluation of Rifampicin-Containing Fixed-Dose Combinations Using Biopharmaceutic Classification System Based Approach. *Clinical Research and Regulatory Affairs*, 24(2-4):61–76, 2007.

[103] S. Agrawal and R. Panchagnula. Implication of biopharmaceutics and pharmacokinetics of rifampicin in variable bioavailability from solid oral dosage forms. *Biopharmaceutics & Drug Disposition*, 26(8):321–34, 2005.

[104] G. Boman and V. A. Ringberger. Binding of rifampicin by human plasma proteins. *European Journal of Clinical Pharmacology*, 7(5):369–73, 1974.

[105] I. E. Templeton, J. B. Houston, and A. Galetin. Predictive utility of in vitro rifampin induction data generated in fresh and cryopreserved human hepatocytes, Fa2N-4, and HepaRG cells. *Drug Metabolism and Disposition*, 39(10):1921–9, 2011.

[106] M. Shou, M. Hayashi, Y. Pan, Y. Xu, K. Morrissey, L. Xu, and G. L. Skiles. Modeling, prediction, and in vitro in vivo correlation of CYP3A4 induction. *Drug Metabolism and Disposition*, 36(11):2355–70, 2008.

[107] U. Loos, E. Musch, J. C. Jensen, G. Mikus, H. K. Schwabe, and M. Eichelbaum. Pharmacokinetics of oral and intravenous rifampicin during chronic administration. *Klinische Wochenschrift*, 63(23):1205–11, 1985.

[108] R. G. Tirona, B. F. Leake, A. W. Wolkoff, and R. B. Kim. Human organic anion transporting polypeptide-C (SLC21A6) is a major determinant of rifampin-mediated pregnane X receptor activation. *The Journal of Pharmacology and Experimental Therapeutics*, 304(1):223–8, 2003.

[109] A. Collett, J. Tanianis-Hughes, D. Hallifax, and G. Warhurst. Predicting P-glycoprotein effects on oral absorption: correlation of transport in Caco-2 with drug pharmacokinetics in wild-type and mdr1a(-/-) mice in vivo. *Pharmaceutical Research*, 21(5):819–26, 2004.

[110] A. Nakajima, T. Fukami, Y. Kobayashi, A. Watanabe, M. Nakajima, and T. Yokoi. Human arylacetamide deacetylase is responsible for deacetylation of rifamycins: rifampicin, rifabutin, and rifapentine. *Biochemical Pharmacology*, 82(11):1747–56, 2011.

[111] M. Hirano, K. Maeda, Y. Shitara, and Y. Sugiyama. Drug-drug interaction between pitavastatin and various drugs via OATP1B1. *Drug Metabolism and Disposition*, 34(7):1229–36, 2006.

[112] M. L. Reitman, X. Chu, X. Cai, J. Yabut, R. Venkatasubramanian, S. Zajic, J. A. Stone, Y. Ding, R. Witter, C. Gibson, K. Roupe, R. Evers, J. A. Wagner, and A. Stoch. Rifampin's acute inhibitory and chronic inductive drug interactions: experimental and model-based approaches to drug-drug interaction trial design. *Clinical Pharmacology and Therapeutics*, 89(2): 234–42, 2011.

[113] T. Rodgers, D. Leahy, and M. Rowland. Physiologically based pharmacokinetic modeling 1: predicting the tissue distribution of moderate-to-strong bases. *Journal of Pharmaceutical Sciences*, 94(6):1259–76, 2005.

[114] M. J. Taylor, S. Tanna, and T. Sahota. In vivo study of a polymeric glucose-sensitive insulin delivery system using a rat model. *Journal of Pharmaceutical Sciences*, 99(10):4215–27, 2010.

[115] D. Scotcher, S. Billington, J. Brown, C. R. Jones, C. D. A. Brown, A. Rostami-Hodjegan, and A. Galetin. Microsomal and cytosolic scaling factors in dog and human kidney cortex and application for in vitro-in vivo extrapolation of renal metabolic clearance. *Drug Metabolism and Disposition*, 45(5):556–68, 2017.

[116] M. Meyer, S. Schneckener, B. Ludewig, L. Kuepfer, and J. Lippert. Using expression data for quantification of active processes in physiologically based pharmacokinetic modeling. *Drug Metabolism and Disposition*, 40(5):892–901, 2012.

[117] B. Prasad, R. Evers, A. Gupta, C. E. C. A. Hop, L. Salphati, S. Shukla, S. V. Ambudkar, and J. D. Unadkat. Interindividual variability in hepatic organic anion-transporting polypeptides and P-glycoprotein (ABCB1) protein expression: quantification by liquid chromatography tandem mass spectroscopy and influence of genotype, age, and sex. *Drug Metabolism and Disposition*, 42(1):78–88, 2014.

[118] B. Prasad, K. Johnson, S. Billington, C. Lee, G. W. Chung, C. D. A. Brown, E. J. Kelly, J. Himmelfarb, and J. D. Unadkat. Abundance of drug transporters in thehuman kidney cortex as quantified by quantitative targeted proteomics. *Drug Metabolism and Disposition*, 44 (12):1920–4, 2016.

[119] M. Otsuka, T. Matsumoto, R. Morimoto, S. Arioka, H. Omote, and Y. Moriyama. A human transporter protein that mediates the final excretion step for toxic organic cations. *Proceedings of the National Academy of Sciences of the United States of America*, 102(50):17923–8, 2005.

[120] S. Masuda, T. Terada, A. Yonezawa, Y. Tanihara, K. Kishimoto, T. Katsura, O. Ogawa, and K. I. Inui. Identification and functional characterization of a new human kidney-specific H+/organic cation antiporter, kidney-specific multidrug and toxin extrusion 2. *Journal of the American Society of Nephrology*, 17(8):2127–35, 2006.

[121] M. Nishimura and S. Naito. Tissue-specific mRNA expression profiles of human ATP-binding cassette and solute carrier transporter superfamilies. *Drug Metabolism and Pharmacokinetics*, 20(6):452–77, 2005.

[122] N. Kolesnikov, E. Hastings, M. Keays, O. Melnichuk, Y. A. Tang, E. Williams, M. Dylag, N. Kurbatova, M. Brandizi, T. Burdett, K. Megy, E. Pilicheva, G. Rustici, A. Tikhonov, H. Parkinson, R. Petryszak, U. Sarkans, and A. Brazma. ArrayExpress update–simplifying data submissions. *Nucleic Acids Research*, 43(Database issue):D1113–6, 2015.

[123] L. Wang, B. Prasad, L. Salphati, X. Chu, A. Gupta, C. E. C. A. Hop, R. Evers, and J. D. Unadkat. Interspecies variability in expression of hepatobiliary transporters across human, dog, monkey, and rat as determined by quantitative proteomics. *Drug Metabolism and Disposition*, 43(3):367–74, 2015.

[124] National Center for Biotechnology Information (NCBI). Expressed Sequence Tags (EST) from UniGene, 2019.

[125] M. Nishimura and S. Naito. Tissue-specific mRNA expression profiles of human phase I metabolizing enzymes except for cytochrome P450 and phase II metabolizing enzymes. *Drug Metabolism and Pharmacokinetics*, 21(5):357–74, 2006.

[126] A. D. Rodrigues. Integrated cytochrome P450 reaction phenotyping: attempting to bridge the gap between cDNA-expressed cytochromes P450 and native human liver microsomes. *Biochemical Pharmacology*, 57(5):465–80, 1999.

[127] M. Nishimura, H. Yaguti, H. Yoshitsugu, S. Naito, and T. Satoh. Tissue distribution of mRNA expression of human cytochrome P450 isoforms assessed by high-sensitivity real-time reverse transcription PCR. *Yakugaku Zasshi: Journal of the Pharmaceutical Society of Japan*, 123(5): 369–75, 2003.

[128] K. Rowland Yeo, R. L. Walsky, M. Jamei, A. Rostami-Hodjegan, and G. T. Tucker. Prediction of time-dependent CYP3A4 drug-drug interactions by physiologically based pharmacokinetic modelling: impact of inactivation parameters and enzyme turnover. *European Journal of Pharmaceutical Sciences*, 43(3):160–73, 2011.

[129] D. J. Greenblatt, L. L. von Moltke, J. S. Harmatz, G. Chen, J. L. Weemhoff, C. Jen, C. J. Kelley, B. W. LeDuc, and M. A. Zinny. Time course of recovery of cytochrome p450 3A function after single doses of grapefruit juice. *Clinical Pharmacology and Therapeutics*, 74(2): 121–9, 2003.
